# Identifying clusters of multimorbid disease and differences by age, sex, and socioeconomic status: A systematic review

**DOI:** 10.1371/journal.pone.0329794

**Published:** 2025-08-22

**Authors:** Nataysia Mikula-Noble, Vicki Cormie, Rebecca Eilidh McCowan, Colin McCowan

**Affiliations:** 1 Division of Population and Behavioural Sciences, School of Medicine, University of St Andrews, St Andrews, United Kingdom; 2 School of Medicine, The Chancellor’s Building, University of Edinburgh, Edinburgh, United Kingdom; 3 Library, University of St Andrews, St Andrews, United Kingdom; 4 School of Medicine, University of Glasgow, Glasgow, United Kingdom; McMaster University, CANADA

## Abstract

**Background:**

The prevalence of multimorbidity has been growing due to the ageing population and increasingly unhealthy lifestyles. There is interest in identifying clusters of disease and how they are influenced.

**Aims:**

This systematic review aims to (i) investigate the most common clusters in the adult population with multimorbidity (ii) identify methods used to define clusters (iii) examine if clusters differ based on age, sex and socioeconomic status.

**Methods:**

We searched Medline, Embase, SCOPUS, Web of Science Core Collection, and CINAHL using concepts of multimorbidity and clustering techniques to identify relevant papers. Secondary data, including commonly reported clustering techniques, identified clusters, and other characteristics were extracted. All studies were quality assessed using the Newcastle-Ottawa Bias scale.

**Results:**

From a total of 24,231 papers, 125 were included in the review. There was a total of 918 different clusters identified, which were categorized into 59 broad groups. A cardiometabolic cluster appeared most frequently within the identified studies and across age strata. The most common clustering technique was Latent Class Analysis (n = 51). Disease cluster prevalence appeared to differ based on age, whereas no differences could be identified by sex.

**Conclusion:**

Across the 125 papers identified, irrespective of clustering method, a relatively common set of clusters of disease were found. The Cardiometabolic cluster was the most frequently identified cluster across all age groups. Studies that stratified participants by age or sex identified distinct clusters within each subgroup, which differed from those observed in clusters formed from the general adult population (18+).Latent class analysis was the most common clustering technique within this review, but it was not explored if different clustering methods led to different clusters. Further work is needed to distinguish the most prevalent clusters within specific stratified cohorts of different ages, sex, and socioeconomic status; nonetheless, data strongly suggests that there are different clusters that arise dependent on stratifications. With the expected increasing burden of multimorbidity, healthcare services may need to think about the most prevalent disease combinations within certain strata and how joint-specialist services can be tailored to treat those common conditions.

## Introduction

The burden multimorbidity places on healthcare services globally is increasing due in part to the ageing population, the increased incidence of chronic conditions, and less healthy lifestyles [1]. According to the World Health Organization (WHO), multimorbidity is defined as the co-occurrence of two or more long-term health conditions affecting an individual [1]. Patients affected by multimorbidity are subject to more complex management and treatment regimens, and more hospitalizations which can lead to fragmented care and miscommunication between the multidisciplinary teams [1].

A group of interrelated conditions that serve as a catalyst for developing multimorbidity is called a disease cluster [2]. The various long-term health conditions that comprise a multimorbid disease cluster will depend on how multimorbidity is primarily measured, which conditions are considered and possibly by the clustering technique employed. Understanding which conditions comprise common disease clusters provides insights upon common physiological pathways behind concordant clusters of diseases [3], and could direct research towards the optimisation of pharmacological treatment of concordant multimorbid disease clusters and efficient communication with the multi-disciplinary team’s (MDT’s) of different specialties.

Disease clusters are determined by a range of exploratory data clustering techniques which are used to identify patterns in data where there is “no defined dependant variable” [4–5]. Clustering techniques include: latent class analysis, k-means clustering, fuzzy-c means clustering, hierarchical cluster analysis, and association rule mining.

Multimorbidity is known to be more prevalent in older people [6], females [7], and people from low socioeconomic backgrounds [8]. However there is less known on whether these factors also influence types of disease cluster within a population with multimorbidity.

This systematic review aimed to determine what clusters of disease were found in multimorbid populations, what techniques were used to identify clusters and whether data were stratified by age, sex, and socioeconomic status. Specific research questions were:

- What were the most common clusters of disease in the overall population?
- What methods were used to identify clusters of disease in a multimorbid population of adults?
- Do clusters for a population of multimorbid individuals differ based on age, sex and socioeconomic status?

## Methods

This systematic review assessed the literature for studies conducted in human adults diagnosed with multimorbidity where a clustering technique was used to identify common clusters of disease. This review was registered with PROSPERO under the ID code: CRD4202337851 and followed the Preferred Reporting Items for Systematic Reviews and Meta-Analyses (see Appendix 13).

### Eligibility criteria

Inclusion and exclusion criteria for screening papers were defined prior to starting the screening process (see Table 1). Primary eligibility criteria were the reported use of a clustering algorithm to identify common clusters of diseases within a population of adults (15+ to 18 + years old) with multimorbidity. Studies were included if they reported on primary data (e.g., Interviews, surveys) and secondary data (e.g., healthcare records, Government reports) documented in cross-sectional and cohort studies. Systematic reviews were excluded as well as opinion pieces and editorials.

**Table 1:** Inclusion and exclusion criteria.

	Inclusion	Exclusion
**Study Design**	• Cross-sectional studies • Cohort studies (prospective and retrospective) • Longitudinal studies	• Expert opinions • Qualitative studies • Experimental studies (e.g.,: randomized control trials) • Critical reviews • Systematic reviews • Meta-analysis
**Methodology**	• Study defines multimorbidity as 2 + co-existing conditions • Study includes how many conditions were involved in determining whether a person was multimorbid or not • Studies must list either the specific diseases that compose of the disease clusters, or list specific disease cluster titles (e.g., Cardiometabolic) • Records specific clustering techniques (e.g., latent class analysis, k-means clustering)	• Study did not report on a statistical technique used to identify clusters • Study only describes the number of clusters present, and does not name the clusters they found • Study only investigated comorbidities related to a single condition of choice
**Population**	• 15 + − 18+ (whole population) • Population from public (e.g., census report) • Population from primary care service (e.g., general practitioner clinic) • Population from secondary care service (e.g., hospital) • Self-reported records • Clinical records	• Infants and Children • Animal Research • Population is based on a specific health condition • Population is based on a single body function/organ
**Publication**	• Peer-reviewed journal articles • Accessible in English • Full text available to access • All countries • Articles published until March 14, 2024	• Not peer-reviewed, • Not accessible in English • Full text not available • Grey literature

Other inclusion criteria were multimorbidity (defined as per the WHO guidelines as 2 + co-existing conditions), a stated number of diseases included in their measurement of multimorbidity, the use of a specified clustering technique, and a list of the commonly identified clusters.

Only studies that were written in the English language were included but there were no restrictions on the publication date.

### Search strategy

The search strategy for this review was developed in collaboration with a specialist medical librarian (Vicki Cormie) from the University of St Andrews. The electronic databases searched were OVID Medline, OVID Embase, SCOPUS, Web of Science Core Collection, and CINAHL with searches initially conducted during August 2022. The searches were repeated in March 2024 to identify new articles published when the review was in progress.

The search criteria were refined by implementing MeSH (Medical Subject Headings) terms, Boolean logic, and key subject descriptors.

The final search criteria highlighted a combination of two elements: the concept of multimorbidity and clusters of disease, and clustering techniques. All the final scope search terms are included in S4 Appendix.

### Screening and study selection

Duplicates from the original searches were removed and the remaining articles were compiled into *Endnote 20* [9] and then were imported to *Covidence* [10] for screening*.* All titles and abstracts were screened by two reviewers (NMN, CMC or RMC) with disagreements resolved by the third reviewer. Full papers were retrieved, read and screened independently by two reviewers (NMN, CMC). Any disagreement that arose was resolved through discussion between the initial reviewers, and involved a third reviewer’s opinion (RMC). Additional papers were identified through citation chaining [11–12]. Papers identified in the repeated search were screened by a single reviewer (NMN).

### Data extraction and synthesis

Two authors (NMN, CMC) extracted information from the first 10 papers (8%) of selected papers and met to ensure consistency of data retrieval. The remainder of the included articles were extracted by one author (*NMN*) into a predefined formatted spreadsheet.

Information extracted from each publication included: (1) Title, (2) Study design, (3) Country and Study Year, (4) Multimorbid Population, (5) Age group, (6) Number of Patients, (7) Diagnostic Classification Tool Used, (8) Disease Count Index, (9) Number of Diseases Included in Disease Count, (10) Cluster Technique(s), (11) Can each person be in only one cluster?, (12) Clusters Identified in General Population, (13) Number of Clusters Identified, (14) Is There a Priori Decision to Look for a Set Number of Clusters?, (15) Average/Most Common Age Group Per Cluster, (16) Clusters Stratified by Age, (17) Prevalent Sex per Cluster, (18) Clusters Stratified by Sex, (19) Prevalent Socioeconomic Status per Cluster, (20) Clusters Stratified by Socioeconomic Status.

We reported on the extracted characteristics giving both the number and the proportion of all identified papers. As some papers reported on multiple characteristics and some did not report on all categories, not all totals of the reported characteristics would equal the total number of papers identified. In regard to reporting disease clusters, we reported the top five clusters of disease per each paper, and within each disease cluster we reported up to the five most prevalent conditions in order to standardise the data between all papers.

### Methods of data reporting to determine the top 5 clusters

Each of the selected studies had the five most common clusters extracted based on the prevalence within the stated population (see S2 Table). There were no ties identified in any papers although some papers reported fewer than five clusters. Clusters for strata based on sex, age or socioeconomic status were similarly extracted. Each of the reported clusters were then placed into a broader classification to allow for easier comparison across papers (see S4 Table). Broader cluster classifications across all selected papers and for age, sex and socioeconomic strata were reported. Broader cluster classifications reported at least twice were shown in results Table 3.

**Table 3:** Reporting how often broader cluster groupings were identified across all papers – grouped by age and sex strata.

	
		Strata reported in papers							
Cluster grouping	18+ (All Adults)	45+ (All Adults)	50+ (All Adults)	60+	65+	Female 45–64	Male 45–64	Female 65+	Male 65+
**Cardiometabolic**	9	7	9	10	14	3	2	4	3
**Diabetes – Hypertension**	7	3	4		6	2	2		
**Musculoskeletal – Neurological – Mental**	5		6	2	6				2
**Cardiovascular**	7				5		3	5	5
**Musculoskeletal**	3		2	4	5	3	2	2	
**Mental Health**	7		2	2					
**Neuropsychiatric**				2	5				
**Respiratory – Asthma – COPD**	4	2	4	4					
**Age-Related – Degenerative**	4				3				
**Asthma – Respiratory – Degenerative**			2					2	2
**Dependence – Substance Misuse**	4						2		
**Cancer**	3	2		2					
**Asthma – Allergy – Hypersensitivity**		3	2						
**Degenerative – Frailty – Mental**								3	2
**Hypertension – Arthritis**					4				
**Neurological – Vascular**								2	2
**Endocrine – Metabolic**					3				
**Mental – GI**					3				
**Musculoskeletal – Degenerative – GI**		2		4					

*Footnote:* Footnote presents the Clusters of Disease reported for one stratum only or by a single paper. Clusters identified in each stratum are presented as a list separated by semi-colons.

*45+ = Neuroendocrine – Cardio (2 papers); Vascular (2 papers); Cancer – Cirrhosis – Diabetes; Angina – Asthma – Chronic lung disease – Arthritis – Depression; Cancer – Peripheral Vascular Disease – Heart Failure, Neuropsychiatric, respiratory*

*50+ = Cardiorespiratory (2 papers); Cancer; Hepatorenal; Vascular- Metabolic; GI-Arthritis; Cancer/Heart Disease/Stroke; Arthritis-Cataracts; Neurodegenerative; Cancer-GI-Neurologic, Neuropsychiatric, Age-Related – Degenerative, Cancer, Hepatorenal*

*60+ = Respiratory – Cancer (2 papers); Respiratory – GI (2 papers); CVS – Anemia – Dementia; Hepatic – Raspatory – GI; Hypertension – Peptic Acid Disease; Asthma/Arthritis/Rheumatism/Chronic Lung Disease; Cardiopulmonary/Mental/Degenerative; Cerebrovascular/Metabolic; Degenerative/Cancer; Dyslipidemia/Hypertension/Arthritis/ Rheumatism/MI; Endocrine – Renal; Metabolic and Sleep Disorders; MSK – Respiratory – GI; Vascular; Vision Problems/Hearing Problems/ Problems with Teeth and Gums, Hypertension – Arthritis, Hepatorenal, Age-Related – Degenerative, Cardiovascular, Diabetes – Hypertension*

*M65+ = Anaemia – Frailty; GI/Hearing loss/pain; Mechanical; Psychiatric disorders, Mental Health, Neuropsychiatric, Respiratory – Asthma – COPD, Dependence – Substance Abuse*

*F45-64 = Allergy-Hypersensitivity – Dermatitis and Eczema; Benign neoplasms, dermatitis, eczema; Cardiovascular – Mental Health; GI disorders; Metabolic/Mental/Substance abuse/Dorsopathies, Respiratory – Mental Health; Respiratory/GU/Ear disease; Respiratory/Oral cavity diseases/Dermatitis, Dependence – Substance Abuse, Asthma – Respiratory – Degenerative, Mental Health*

*Musculoskeletal – Neurological – Mental*

*M45-64 = Allergy-hypersensitivity – Eye disease; Ear, eye, and skin infections; GI/GU/Respiratory/Cancer, MSK/Neurological/Mental/CKD/Stroke/Cancer; Psychiatric/Respiratory/Oral cavity diseases; Respiratory/Mental Health/Liver; Soft tissue/GU/MSK, Respiratory – Asthma – COPD*

All articles were screened for bias using the Newcastle-Ottawa Bias scale [13] by one person (NMN) [14] (see S5 Table). This tool assessed the quality of each included study, regarding (i) paper selection (ii) comparability, and (iii) outcome. Each study was assigned a global risk of bias score: high-quality or low-quality, based on whether they accumulated a total of seven stars.

There was no public involvement in the development or conduct of the review and no ethics permissions were required. There was no sponsorship, in study design; in the collection, analysis, and interpretation of data; in the writing of the report; and in the decision to submit the paper for publication.

## Results

### Study selection

**Fig 1:**
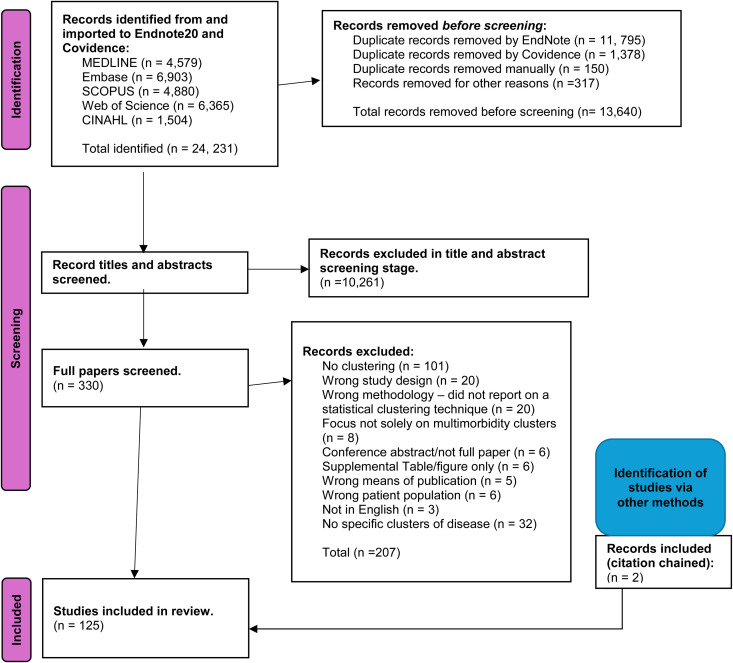
PRISMA flow diagram.

There were 24,231 articles identified from the bibliographic search. Firstly, 317 were primarily excluded prior to title and abstract screening since they were records returned from Web Of Science which were images of figures and tables reported in the Figshare database [15–16]. Secondly, the duplicates were excluded: 11,795 duplicates were identified and deleted by EndNote, 1378 duplicates were identified by Covidence, and 150 duplicates were identified manually. Following the removal of 10,261 studies due to title and abstract screening, and also the removal 207 papers due to full paper screening, 123 studies were identified for inclusion. Two extra studies were identified and included through citation chaining, and were added to the group of 123 studies, creating a grand total of 125 studies being included in this systematic review. Full tables revealing the full search strategy and a list of all the papers exported onto EndNote and reasons why they were included or excluded are listed in S4 Appendix and Fig 1 respectively. There were no missing data in this review.

### Study characteristics

S1 and S2 Tables include all the study characteristics, as well as the five most common clusters for each paper that was extracted from the 125 included studies. Table 2 summarises the study characteristics of the included studies. Of the 125 studies, 74 (59%) were cross-sectional and 51 (41%) were cohort studies. Population sizes ranged from 328 [17] to 4,280,985 [18] patients, with 10% having more than 500,000 patients. 61 papers (49%) used primary or secondary care electronic health records (EHR) to identify conditions and multimorbidity. The majority of studies were from high income countries although some involved data collected from multiple continents.

**Table 2:** Summary of study characteristics.

	
**Study Design**		n = 125
	Cohort	51 (41%)
	Cross-sectional	74 (59%)
**Population Size**		n = 125
	Small (328–9,999):	58 (46%)
	Medium (10,000–499,999):	55 (44%)
	Large (more than 500,000):	12 (10%)
**Age groupings of Population**		n = 125
	15 to 18 years +	43 (34%)
	45 years +	12 (10%)
	50 years +	22 (18%)
	60 years +	48 (38%)
**Continent**		n = 125
	North America	19 (15%)
	South America	6 (5%)
	Asia	30 (24%)
	Australasia	9 (7%)
	Europe	54 (43%)
	Africa	3 (2%)
	Multiple Continents	4 (3%)
**Setting: Data Collection method**		^*^ *some papers used multiple means of data collection*
	Primary Care Database	29
	Secondary Care Database	32
	Self-reported	63
	Interview (telephone or face-to-face)	11
	Other	8
**Diagnostic Classification Tools**		n = 125
	International Classification of Diseases 9/10	34 (27%)
	International Classification of Primary Care	7 (28%)
	Other Classification Tool	3 (12%)
	N/A	81 (65%)
**Disease Count Index**		^*^ *some papers used multiple disease counts*
	Self-Selected	48
	Salisbury et al. [19]	6
	World Health Organisation SAGE Study [20]	6
	Barnett [21]	4
	Calderon-Larranga et al. [22]	4
**’**	O’Halloran Criteria [23]	4
	Charlson [24]	4
	Diederichs et al.[25]	3
	Other Index	51
**Number of Conditions in Disease Count Strategy**		n = 125
	1–10	15 (12%)
	11–20	65 (52%)
	21–50	23 (18%)
	51+	22 (18%)
**Clustering Technique**		^*^ *some papers used multiple techniques*
	Latent Class Analysis	51
	Hierarchical cluster analysis	20
	Fuzzy c-means	3
	Association rule mining	8
	Multiple correspondence analysis	6
	Exploratory factor analysis	24
	Cluster analysis	6
	K-means clustering	9
	Network Analysis	3
	Factorial Analysis	5
	Multiple Trajectory Analysis	1
**Number of Clusters Identified**		^*^ *some papers used multiple strata*
	1–5	75
	6–10	29
	11–20	14
	21+	4
**Stratification Variables**		^*^ *some papers used multiple strata*
	Age	19
	Sex	24
	Socioeconomic Status	4
	No stratification	104

* *Footnote: In all cases this systematic review analyzed 125 papers. In some limited circumstances the identified papers utilized multiple clustering or stratification techniques and, as a result, where these instances occurred, the data will not necessarily summate to 125.*

### Measures of multimorbidity

Multimorbidity was measured using different strategies across the 125 articles, as per the findings summarised in Table 2. There were 51 different disease count indexes used across the papers with some using multiple indexes. The most common disease count indexes used were Salisbury (n = 6, 5%) [19] and WHO (n = 6, 5%) [20]. There were differences in how many diseases were examined in the papers, with just over half (65, 52%) using 11–20 diseases. The most common disease classification system used by the electronic health records within the studies were ICD9/ICD10 codes (n = 34, 27%); however, over half of the papers did not report on any system used (n = 81, 65%).

### Clustering methods

Latent Class Analysis was the most commonly occurring technique used to identify clusters in the study populations (n = 51, 41%), followed by Exploratory Factor Analysis (n = 24, 19%) and Hierarchical Cluster Analysis (n = 20, 16%). All other clustering techniques are listed on Table 2.

There were 24 papers (19%) that stratified data by sex before deploying their clustering techniques to determine the most prevalent clusters. Papers were also stratified by age (n = 19) and socioeconomic status (n = 4).

### Identified clusters of disease

There were a total of 949 clusters identified across the 125 papers. These were classified into broad cluster groupings with 59 different categories identified (see S4 Table). The most commonly reported clusters across all populations being “cardiometabolic”, “diabetes-hypertension” and “musculoskeletal-neurological-mental” (see Table 3).

Data from the papers were extracted to identify clusters that were reported after stratification by age and/ or sex. There were five different age strata reported and four strata by age-and-sex. The cardiometabolic cluster was present across all strata. There were differences for the most commonly reported clusters by ages with some clusters only featuring in younger or older strata, i.e., Dependence – Substance Misuse versus Neurological – Vascular. Table 3 reports groupings of disease clusters that are reported in more than two studies or appear in at least two strata within two separate studies. The footnote below Table 3 lists the groupings of disease clusters that were reported for one stratum only or by a single paper.

Looking at disease clusters by age and sex most were present within the same age group for both men and women. However, men aged 65 + were reported as having a Musculoskeletal – Neurological – Mental cluster that was not present in women, who had a musculoskeletal only cluster not found in men. Men aged 45–64 had disease clusters for Cardiovascular disease and Dependence- Substance misuse that was not seen in women (see Table 3).

Diabetes- Hypertension was identified as a cluster for both male and female groups aged 45–64 but not in the older age category for both sexes. Conversely Asthma – Respiratory – Degenerative, Degenerative – Frailty – Mental and Neurological – Vascular clusters were identified in the older age group for both sexes but not the younger groups.

Latent Class Analysis was the most common clustering technique observed with 11 different clustering techniques identified within the 125 papers reviewed. There were three key considerations within the clustering techniques i) was a decision made prior to undertaking the clustering analysis regarding the number of clusters to include, and ii) whether each person in the cohort could only belong to one cluster (but the same disease could belong to multiple clusters), or iii) whether there is no overlap of diseases within each clusters, and each disease belongs to one and only one cluster. Eight papers reported using multiple clustering techniques within the same dataset, either combining techniques or comparing two or more techniques against the same cohort. This allowed these papers to compare the differences in clusters identified between the techniques.

### Bias

The Newcastle-Ottawa Bias scale [13] was used to assess bias (see S5 Table). Five papers were categorized as high-quality (4%), and the rest of the papers were low-quality (n = 120). Low-quality papers arose because most papers did not involve long-term follow-ups, and therefore no stars would be allocated to Section 3.3 of the scale. For the small amount of papers that did involve follow-ups, many of the papers had more than 20% loss to follow-up, which is considered a serious threat to validity [26], and therefore stars could not be allocated for those papers in section 3.3 of the scale.

## Discussion

This review found the most frequently reported cluster in adults was a cardiometabolic disease cluster with other common clusters including diabetes-hypertension, musculoskeletal-neurological-mental, and cardiovascular. There were differences in the identified clusters when populations were stratified by age prior to clustering, although there were less obvious differences by sex. The cardiometabolic cluster was more frequently reported in studies with older strata of patients. Cardiovascular, asthma-respiratory-degenerative, degenerative-frailty-mental, and neurological-vascular were other disease clusters more frequently reported by studies n older patients.

There were differences in clusters identified by age and sex, especially when reporting on mental health related conditions. There was insufficient data to make any observations relating to socio-economic strata due to the lack of common definitions and measurements of socio-economic class. Similarly. we did not explore differences in identified clusters by clustering technique.

### Strengths

The strengths of this review were as follows: (i) it consisted of a comprehensive search, using five different databases, with data collected without geographical restriction, including multiple jurisdictions, ethnicities and race; (ii) a systematic approach was used to determine papers including; the use of Boolean and MeSH search terms in the search algorithm, screening and study selection; (iii) the involvement of two main authors as data extractors (and the involvement of a third author when conflicts arose).

### Limitations

There was heterogeneity in the identified studies which made it difficult to determine the most common clusters. This related to the clusters identified in the original reports and also a large variation in how they were reported which meant further classification into groups was needed which is always a subjective process. Differences in approach of the studies included but were not limited to: (i) diagnostic classification tools, (ii) disease count index (iii) conditions within disease count strategy (iv) different clustering techniques, and (v) identifying a different number of clusters within their population with or without a priori number of initial clusters.

There were also issues relating to the secondary data collected within the underlying papers including (i) wide age bands such as 18 + may simply reflect the clusters of disease that exist in the older patients within the study population (ii) clusters that are seen between primary and secondary care data may differ because conditions treated within primary and secondary settings will differ. A previous study has noted that using the diagnostic codes available in primary vs secondary care leads to different diseases being identified within the population [27].

It was not possible to compare the effect of clustering techniques on the same population strata and same disease count within the scope of this review. However, there were a number of papers identified within this study that used different techniques within the same datasets and it may be that applying different clustering techniques within the same dataset is a better way to explore how this may affect the reported clusters.

### Previous literature

The most recent study that investigated trends of multimorbidity within social determinants of health including sex, age, and socioeconomic status was published by Alvarez-Galvez et al. in March 2023 and consisted of 97 articles [28]. This study differentiates from the Alvarez-Galvez et al. study because this current study investigates which specific age/sex/socioeconomic status strata exist in the literature, and which clusters are identified within strata with a common denominator; whereas as the Alvarez-Galvez study only provided general commentary based on the prevalence of multimorbidity associated with the specific social determinants. Identifying clusters within common age groupings (such as 45–65, or 50+) allows for a more accurate comparison of clusters between studies. There was an overlap of 25 studies between this paper and the previous study. 100 more papers were identified that involved clusters of disease and clustering techniques in this paper. The slim overlap of selected articles between the two papers is most probably explained by the differences between the search strategies such as: (i) Alvarez-Galvez et al. required the involvement the concept of multimorbidity as well as commentary on one or multiple social determinants of health; (ii) the Alvarez-Galvez et al. paper examines comorbidity profiles based on a specific condition (e.g., Cardiovascular disease) or a specific group of people (e.g., Homeless cohort); whereas this paper investigates clusters within a general population; (iii) Alvarez-Galvez et al. papers did not require the listing of specific clusters of disease for inclusion, compared to this study, which only included papers that explicitly stated the most common clusters of disease within the population.

A table summarizing all the past systematic reviews that investigated disease clusters within populations coming from both primary and secondary care is in S6 Table [28–33]. Four out of the six past systematic reviews also have the Cardiometabolic cluster as the most commonly identified cluster [28,30–32]. However, the most commonly reported clustering technique stated within the past reviews is exploratory factor analysis, which is documented in three out of the five papers that investigate clustering techniques [29–31]*.*

### Implications for practice, policy, and further research

A previous review had highlighted that age stratification when detailing profiles of multimorbidity would be useful to allow for better understanding differences between different studies [29]. This review similarly found it difficult to compare findings due to the variety of age ranges within studies and different groupings within that range. A more standardized grouping of age in reporting would allow for easier investigation of clusters of disease across studies. Within the papers included in this study there was a lack of clustering focussed solely on younger age populations. There is a well-established problem with variation in measurement of multimorbidity within the literature which makes undertaking comparisons across reported results as in this paper difficult [34]. Individual papers will always tend to use age groupings and markers of socioeconomic status that are readily available to them and pertinent to their study aims but a more standardised approach to using categories would improve future comparability.

This review included studies that reported on the co-existence of conditions to identify disease clusters but did not explore the accumulations of these conditions over time. There are now a number of papers that have reported on the development of disease trajectories including subsequent impact on health outcomes [35–39]. However, a recent review of methods to identify disease trajectories concluded that approaches using common data on similar populations is needed so that comparisons across studies are easier to conduct [40].

With the expected increasing burden of multimorbidity on healthcare services, it may be important for health services to consider the combination of diseases that are most common within certain age and sex groups, and how services can be tailored to treat those common conditions. This report highlights that clusters involving mental health are more common in younger age groups. Services that are tailored to specific population strata and the most prevalent clusters of disease improve care through i) earlier identification of common clusters of disease ii) increased efficacy of assessment and treatment, and iii) a holistic treatment process for patients that will encompass their multiple conditions all at once and therefore allow for less healthcare visits, shorter waiting times, and increase their quality of life. Health providers have started to address the challenge moving away from single condition pathways of care but despite this being a long standing issue there is still much work to be done to remove the problem of disjointed care for an individual patient between specialist teams [41].

## Conclusion

Across 125 papers there was relative consistency of clusters identified, despite the use of different clustering techniques with a cardiometabolic cluster being the most frequently identified. However, this was dependent on the individual conditions that were studied in each of the papers and the age and sex characteristics of participants.. Studies that stratified participants by age or sex identified distinct clusters within each subgroup, which differed from those observed in clusters formed from the general adult population (18+). There were a variety of different clustering techniques used but latent class analysis was the most common. Further work is needed to distinguish the most prevalent clusters within specific stratified cohorts of different ages, sex, and socioeconomic status; nonetheless, data strongly suggests that there are different clusters that arise dependent on stratifications.

Having a better understanding of which conditions exist in tandem may allow services to efficiently and holistically manage patients which makes the quality of life easier for patients and hopefully improves care.

## Supporting information

S1 Appendix PROSPERO registration. (DOCX)

S2 Appendix PRISMA 2020 main checklist. (DOCX)

S3 Appendix PRISMA abstract checklist. (DOCX)

S4 Appendix Database search terms and results. (DOCX)

S1 Table Data extraction – study characteristics. (DOCX)

S2 Table Data extraction – clusters of disease. (DOCX)

S3 Table List of included and excluded studies. (XLSX)

S4 Table Broad cluster groupings identified across papers and stratified by age and sex. (DOCX)

S5 Table Bias screening. (DOCX)

S6 Table Previous systematic reviews. (DOCX)

## References

[1] Mercer S. Multimorbidity: technical series on safer primary care. Geneva: World Health Organization; 2016. https://iris.who.int/bitstream/handle/10665/252275/9789241511650-eng.pdf?sequence=1

[2] MacMahon S. Sciences AoM multimorbidity: a priority for global health research. MacMahon S, ed. London: Creative Commons Attribution 4.0 International; 2018.

[3] Aga F, Dunbar SB, Kebede T, Gary RA. The role of concordant and discordant comorbidities on performance of self-care behaviors in adults with type 2 diabetes: a systematic review. Diabetes Metab Syndr Obes. 2019;12:333–56. doi: 10.2147/DMSO.S186758 31114271 PMC6497834

[4] Bharathwaj M. Clustering techniques: towards data science. 2020.

[5] Nylund-Gibson K, Choi AY. Ten frequently asked questions about latent class analysis. Transl Issues Psychol Sci. 2018;4(4):440–61.

[6] Makovski TT, Schmitz S, Zeegers MP, Stranges S, van den Akker M. Multimorbidity and quality of life: systematic literature review and meta-analysis. Ageing Res Rev. 2019;53:100903. doi: 10.1016/j.arr.2019.04.005 31048032

[7] Bezerra de Souza DL, Oliveras-Fabregas A, Espelt A, Bosque-Prous M, de Camargo Cancela M, Teixidó-Compañó E, et al. Multimorbidity and its associated factors among adults aged 50 and over: a cross-sectional study in 17 European countries. PLoS One. 2021;16(2):e0246623. doi: 10.1371/journal.pone.0246623 33571285 PMC7877625

[8] Nguyen H, Manolova G, Daskalopoulou C, Vitoratou S, Prince M, Prina AM. Prevalence of multimorbidity in community settings: a systematic review and meta-analysis of observational studies. J Comorb. 2019;9. doi: 10.1177/2235042X19870934 31489279 PMC6710708

[9] Team TE. EndNote. Philadelphia, PA: Clarivate; 2013.

[10] Innovation VH. Covidence systematic review software. Melbourne, Australia: Veritas Health Innovation; 2021.

[11] Bisquera A, Gulliford M, Dodhia H, Ledwaba-Chapman L, Durbaba S, Soley-Bori M, et al. Identifying longitudinal clusters of multimorbidity in an urban setting: a population-based cross-sectional study. Lancet Reg Health Eur. 2021;3:100047. doi: 10.1016/j.lanepe.2021.100047 34557797 PMC8454750

[12] Garin N, Koyanagi A, Chatterji S, Tyrovolas S, Olaya B, Leonardi M, et al. Global multimorbidity patterns: a cross-sectional, population-based, multi-country study. J Gerontol A Biol Sci Med Sci. 2016;71(2):205–14. doi: 10.1093/gerona/glv128 26419978 PMC5864156

[13] Wells GA, Wells G, Shea B, Shea B, O’Connell D, Peterson J. The Newcastle-Ottawa Scale (NOS) for assessing the quality of nonrandomised studies in meta-analyses. 2014.

[14] Thomas BH, Ciliska D, Dobbins M, Micucci S. A process for systematically reviewing the literature: providing the research evidence for public health nursing interventions. Worldviews Evid Based Nurs. 2004;1(3):176–84. doi: 10.1111/j.1524-475X.2004.04006.x 17163895

[15] Gross B, Pritchett R. Web of science workshop. 2020.

[16] Fagbamigbe AF, Agrawal U, Azcoaga-Lorenzo A, MacKerron B, Özyiğit EB, Alexander DC, et al. Clustering long-term health conditions among 67728 people with multimorbidity using electronic health records in Scotland. PLoS One. 2023;18(11):e0294666. doi: 10.1371/journal.pone.0294666 38019832 PMC10686427

[17] Formiga F, Ferrer A, Sanz H, Marengoni A, Alburquerque J, Pujol R, et al. Patterns of comorbidity and multimorbidity in the oldest old: the Octabaix study. Eur J Intern Med. 2013;24(1):40–4. doi: 10.1016/j.ejim.2012.11.003 23186603

[18] Fränti P, Sieranoja S, Wikström K, Laatikainen T. Clustering diagnoses from 58 million patient visits in Finland between 2015 and 2018. JMIR Med Inform. 2022;10(5):e35422. doi: 10.2196/35422 35507390 PMC9118010

[19] Salisbury C, Johnson L, Purdy S, Valderas JM, Montgomery AA. Epidemiology and impact of multimorbidity in primary care: a retrospective cohort study. Br J Gen Pract. 2011;61(582):e12-21. doi: 10.3399/bjgp11X548929 21401985 PMC3020068

[20] Kowal P, Chatterji S, Naidoo N, Biritwum R, Fan W, Lopez Ridaura R, et al. Data resource profile: the World Health Organization study on global AGEing and adult health (SAGE). Int J Epidemiol. 2012;41(6):1639–49. doi: 10.1093/ije/dys210 23283715 PMC3535754

[21] Barnett K, Mercer SW, Norbury M, Watt G, Wyke S, Guthrie B. Epidemiology of multimorbidity and implications for health care, research, and medical education: a cross-sectional study. Lancet. 2012;380(9836):37–43. doi: 10.1016/S0140-6736(12)60240-2 22579043

[22] Calderon-Larranaga A, Vetrano DL, Onder G, Gimeno-Feliu LA, Coscollar-Santaliestra C, Carfi A. Assessing and measuring chronic multimorbidity in the older population: a proposal for its operationalization. J Gerontol Ser A-Biol Sci Med Sci. 2017;72(10):1417–23. 28003375 10.1093/gerona/glw233 PMC5861938

[23] O’Halloran J, Miller GC, Britt H. Defining chronic conditions for primary care with ICPC-2. Fam Pract. 2004;21(4):381–6. doi: 10.1093/fampra/cmh407 15249526

[24] Charlson M, Szatrowski TP, Peterson J, Gold J. Validation of a combined comorbidity index. J Clin Epidemiol. 1994;47(11):1245–51. doi: 10.1016/0895-4356(94)90129-5 7722560

[25] Diederichs C, Berger K, Bartels DB. The measurement of multiple chronic diseases--a systematic review on existing multimorbidity indices. J Gerontol A Biol Sci Med Sci. 2011;66(3):301–11. doi: 10.1093/gerona/glq208 21112963

[26] Dettori JR. Loss to follow-up. Evid Based Spine Care J. 2011;2(1):7–10. doi: 10.1055/s-0030-1267080 22956930 PMC3427970

[27] MacRae C, Morales D, Mercer SW, Lone N, Lawson A, Jefferson E, et al. Impact of data source choice on multimorbidity measurement: a comparison study of 2.3 million individuals in the Welsh National Health Service. BMC Med. 2023;21(1):309. doi: 10.1186/s12916-023-02970-z 37582755 PMC10426056

[28] Álvarez-Gálvez J, Ortega-Martín E, Carretero-Bravo J, Pérez-Muñoz C, Suárez-Lledó V, Ramos-Fiol B. Social determinants of multimorbidity patterns: a systematic review. Front Public Health. 2023;11:1081518. doi: 10.3389/fpubh.2023.1081518 37050950 PMC10084932

[29] Busija L, Lim K, Szoeke C, Sanders KM, McCabe MP. Do replicable profiles of multimorbidity exist? Systematic review and synthesis. Eur J Epidemiol. 2019;34(11):1025–53. doi: 10.1007/s10654-019-00568-5 31624969

[30] Ng SK, Tawiah R, Sawyer M, Scuffham P. Patterns of multimorbid health conditions: a systematic review of analytical methods and comparison analysis. Int J Epidemiol. 2018;47(5):1687–704. doi: 10.1093/ije/dyy134 30016472

[31] Rajoo SS, Wee ZJ, Lee PSS, Wong FY, Lee ES. A systematic review of the patterns of associative multimorbidity in Asia. Biomed Res Int. 2021;2021:6621785. doi: 10.1155/2021/6621785 34327233 PMC8277911

[32] Prados-Torres A, Calderón-Larrañaga A, Hancco-Saavedra J, Poblador-Plou B, van den Akker M. Multimorbidity patterns: a systematic review. J Clin Epidemiol. 2014;67(3):254–66. doi: 10.1016/j.jclinepi.2013.09.021 24472295

[33] Kanesarajah J, Waller M, Whitty JA, Mishra GD. Multimorbidity and quality of life at mid-life: a systematic review of general population studies. Maturitas. 2018;109:53–62. 29452782 10.1016/j.maturitas.2017.12.004

[34] Ho IS-S, Azcoaga-Lorenzo A, Akbari A, Black C, Davies J, Hodgins P, et al. Examining variation in the measurement of multimorbidity in research: a systematic review of 566 studies. Lancet Public Health. 2021;6(8):e587–97. doi: 10.1016/S2468-2667(21)00107-9 34166630

[35] Ioakeim-Skoufa I, González-Rubio F, Aza-Pascual-Salcedo M, Laguna-Berna C, Poblador-Plou B, Vicente-Romero J, et al. Multimorbidity patterns and trajectories in young and middle-aged adults: a large-scale population-based cohort study. Front Public Health. 2024;12:1349723. doi: 10.3389/fpubh.2024.1349723 38818448 PMC11137269

[36] Owen RK, Lyons J, Akbari A, Guthrie B, Agrawal U, Alexander DC, et al. Effect on life expectancy of temporal sequence in a multimorbidity cluster of psychosis, diabetes, and congestive heart failure among 1·7 million individuals in Wales with 20-year follow-up: a retrospective cohort study using linked data. Lancet Public Health. 2023;8(7):e535–45. doi: 10.1016/S2468-2667(23)00098-1 37393092

[37] Cezard G, Sullivan F, Keenan K. Understanding multimorbidity trajectories in Scotland using sequence analysis. Sci Rep. 2022;12(1):16485. doi: 10.1038/s41598-022-20546-4 36182953 PMC9526700

[38] Carrasco-Ribelles LA, Cabrera-Bean M, Danés-Castells M, Zabaleta-Del-Olmo E, Roso-Llorach A, Violán C. Contribution of frailty to multimorbidity patterns and trajectories: longitudinal dynamic cohort study of aging people. JMIR Public Health Surveill. 2023;9:e45848. doi: 10.2196/45848 37368462 PMC10365626

[39] Lleal M, Baré M, Herranz S, Orús J, Comet R, Jordana R, et al. Trajectories of chronic multimorbidity patterns in older patients: MTOP study. BMC Geriatr. 2024;24(1):475. doi: 10.1186/s12877-024-04925-2 38816787 PMC11137950

[40] Cezard G, McHale CT, Sullivan F, Bowles JKF, Keenan K. Studying trajectories of multimorbidity: a systematic scoping review of longitudinal approaches and evidence. BMJ Open. 2021;11(11):e048485. doi: 10.1136/bmjopen-2020-048485 34810182 PMC8609933

[41] Coulter A, Kramer G, Warren T, Salisbury C. Building the House of Care for people with long-term conditions: the foundation of the House of Care framework. Br J Gen Pract. 2016;66(645):e288-90. doi: 10.3399/bjgp16X684745 27033503 PMC4809714

