# Supplementary material for: Identifying clusters of multimorbid disease and differences by age, sex, and socioeconomic status: A systematic review
Source: PLoS One. 2025 Aug 22;20(8):e0329794. doi: 10.1371/journal.pone.0329794 (PMC12373218; doi:10.1371/journal.pone.0329794)
Supplement: S1 Appendix — (DOCX) [file pone.0329794.s001.docx]

### **Appendix 1: Prospero Document.**


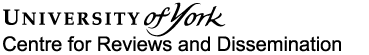


Systematic review

A list of fields that can be edited in an update can be found here

1. * Review title.

Give the title of the review in English

Identifying clusters of multimorbid disease and differences by age, sex, and socioeconomic status: a systematic review

1. Original language title.

For reviews in languages other than English, give the title in the original language. This will be displayed with the English language title.

1. * Anticipated or actual start date.

Give the date the systematic review started or is expected to start.

13/12/2022

1. * Anticipated completion date.

Give the date by which the review is expected to be completed.

30/06/2023

1. * Stage of review at time of this submission.

**This field uses answers to initial screening questions. It cannot be edited until after registration.**

Tick the boxes to show which review tasks have been started and which have been completed.

Update this field each time any amendments are made to a published record.

The review has not yet started: Yes

| **Review stage** | **Started** | **Completed** |
| --- | --- | --- |
| Preliminary searches | No | No |
| Piloting of the study selection process | No | No |
| Formal screening of search results against eligibility criteria | No | No |
| Data extraction | No | No |
| Risk of bias (quality) assessment | No | No |
| Data analysis | No | No |

Provide any other relevant information about the stage of the review here.

1. * Named contact.

The named contact is the guarantor for the accuracy of the information in the register record. This may be any member of the review team.

Nataysia Mikula-Noble Email salutation (e.“. "Dr Sm”th" “r "Joa”ne") for correspondence:

Ms Mikula-Noble

1. * Named contact email.

Give the electronic email address of the named contact.

nmikulanoble13@gmail.com

1. Named contact address

Give the full institutional/organisational postal address for the named contact.

School of Medicine

University of St Andrews

North Haugh

St Andrews

KY16 9TF

1. Named contact phone number.

Give the telephone number for the named contact, including international dialling code.

+44 (0)7534 843667

1. * Organisational affiliation of the review.

Full title of the organisational affiliations for this review and website address if available. This field may be completed ‘s 'N’ne' if the review is not affiliated to any organisation.

Population & Behavioural Science Research Division, School of Medicine, University of St Andrews, UK

Organisation web address:

[https://medic](file:///Users/nataysiamikula-noble/Downloads/ https:/medic)ine.st-andrews.ac.uk/pbs/

1. * Review team members and their organisational affiliations.

Give the personal details and the organisational affiliations of each member of the review team. Affiliation refers to groups or organisations to which review team members belong. **NOTE: email and country now MUST be entered for each person, unless you are amending a published record.**

Ms Nataysia Mikula-Noble. University of St Andrews

Professor Colin McCowan. University of St Andrews

Dr Adeniyi Fagbamigbe. University of St Andrews Ms Rebecca McCowan. University of Glasgow

1. * Funding sources/sponsors.

Details of the individuals, organizations, groups, companies or other legal entities who have funded or sponsored the review.

None

Grant number(s)

State the funder, grant or award number and the date of award

1. * Conflicts of interest.

List actual or perceived conflicts of interest (financial or academic).

None

1. Collaborators.

Give the name and affiliation of any individuals or organisations who are working on the review but who are not listed as review team members. **NOTE: email and country must be completed for each person, unless you are amending a published record.**

Ms Vicki Cormie. University of St Andrews

1. * Review question.

State the review question(s) clearly and precisely. It may be appropriate to break very broad questions down into a series of related more specific questions. Questions may be framed or refined using®(E)COS or similar where relevant.

- What are the most common clusters of disease in the reported populations What methods are used to identify clusters of disease using data from a multimorbid population of adults?
- Do the identified clusters from a population of multimorbid individuals differ based on age?
- What does the literature tell us about clusters of disease based on other characteristics such as sex and socioeconomic cl

1. 16. * Searches.

State the sources that will be searched (e.g. Medline). Give the search dates, and any restrictions (e.g. language or publication date). Do NOT enter the full search strategy (it may be provided as a link or attachment below.)

The following searches will be carried out:

- Embase 1974 to 2022 August Ovid M®INE(R) ALL 1946 to August 18, 202218
- Scopus
- Web of–Science - CINAHL.

Inclusion:• Cross-sectional studies

- Cohort studies (prospective and retrospective)
- Longitudinal studies

Exclusion:

- Expert opinions
- Qualitative studies
- Experimental studies (e.g.: randomized control trials)
- Critical reviews
- Systematic reviews
- Meta-analysis.

17 [1 change]. URL to search strategy.

Upload a file with your search strategy, or an example of a search strategy for a specific database, (including the keywords) in pdf or word format. In doing so you are consenting to the file being made publicly accessible. Or provide a URL or link to the strategy. Do NOT provide links to your search **resu


[lts](C:\\Users\\cm434\\Downloads\\lts.  ht)**[.](C:\\Users\\cm434\\Downloads\\lts.  ht)

[ht](C:\\Users\\cm434\\Downloads\\lts.  ht)tps://www.crd.york.ac.uk/PROSPEROFILES/357851_STRATEGY_20230115.pdf

Alternatively, upload your search strategy to CRD in pdf format. Please note that by doing so you are consenting to the file being made publicly accessible.

Do not make this file publicly available until the review is complete

1. * Condition or domain being studied.

Give a short description of the disease, condition or healthcare domain being studied in your systematic review.

Multimorbidity is when a person has two or more diseases at the same time. For example, the person could have problems with their heart, kidneys, and high blood pressure all at once. Patterns of diseases that are seen in multimorbid people tend to group into clusters. Currently, there are many ways to measure multimorbidity and this leads to debate about how clusters should be formed and what kinds of conditions should be included. This study will identify the most common clusters and clustering methods in a general population by looking at previous studies found online. It will also look at factors that may affect the clusters such as age, sex, and status of wealth.

1. * Participants/population.

Specify the participants or populations being studied in the review. The preferred format includes details of both inclusion and exclusion criteria.

Inclusion:• Adult humans

- 15+ - 18+ (whole population)
- Population from public (e.g. census report)
- Population from primary care service (e.g. general practitioner clinic)
- Population from secondary care service (e.g. hospital)
- Self-reported records
- Clinical records

Exclusion:

- Infants and children
- Animal research
- Population is based on a specific health condition• Population is based on a single body function.

1. 20. * Intervention(s), exposure(s).

Give full and clear descriptions or definitions of the interventions or the exposures to be reviewed. The preferred format includes details of both inclusion and exclusion criteria.

Incl• Study defines multimorbidity as 2+ conditions:

- Study includes how many conditions were involved in determining whether a person was multimorbid or not
- Records specific clustering techniques (e.g. latent class analysis, k-means clustering)

Exclusion:

- Study did not report on a statistical technique used to identify clusters
- Study only describes the number of clusters present, and does not name the clusters they found
- Study only investigated comorbidities related to a single condition of choice.

1. * Comparator(s)/control.

Where relevant, give details of the alternatives against which the intervention/exposure will be compared (e.g. another intervention or a non-exposed control group). The preferred format includes details of both inclusion and exclusion criteria.

None.

1. * Types of study to be included.

Give details of the study designs (e.g. RCT) that are eligible for inclusion in the review. The preferred format includes both inclusion and exclusion criteria. If there are no restrictions on the types of study, this should be stated.

Inclusion: • Cross-sectional studies

- Cohort studies (prospective and retrospective)
- Longitudinal studies

Exclusion:

- Expert opinions
- Qualitative studies
- Experimental studies (e.g.: randomized control trials)
- Critical reviews
- Systematic reviews
- Meta-analysis.

1. 23. Context.

Give summary details of the setting or other relevant characteristics, which help define the inclusion or exclusion criteria.

There will be no restrictions on study location or setting.

24 [1 change]. * Main outcome(s).

Give the pre-specified main (most important) outcomes of the review, including details of how the outcome is defined and measured and when these measurement are made, if these are part of the review inclusion criteria.

The primary outcome of this review is to establish which diseases most commonly co-occur in patients with multimorbidity. From identifying the most prevalent clusters, we will be able to outline future implications for multimorbid interventions on a public health scale, and propose several actions for improving the efficacy and treatment of multimorbidity on a GP-patient-cantered perspective ultimately decreasing the burden on healthcare services.

Measures of effect

Please specify the effect measure(s) for you main outcome(s) e.g. relative risks, odds ratios, risk difference‘ and/or 'number needed to treat.

This is a qualitative review so there are no measures for my outcomes.

25 [1 change]. * Additional outcome(s).

List the pre-specified additional outcomes of the review, with a similar level of detail to that required for main outcomes. Where there are no additional outcomes please state ‘None’ or ‘Not applicable’ as appropriate to the review

As multimorbidity is a relatively novel area of study, this study also has additional outcomes, which will investigate the gaps in existing research, and provide specific suggestions on where further research should be conducted that can ultimately lead to more efficacious diagnoses and treatment for multimorbid patients.

The first additional outcome is determining the methods used to identify clustering of conditions in patients with multimorbidity. This information will identify inconsistencies of the clustering methods used in current studies, and where areas of future research should be directed.

The second additional outcome is collating the commentary provided in each study in regard to cluster variations based on age, sex, and socioeconomic status. This is the first paper that will collate and investigate the differences in multimorbidity between these three more specific stratifications, and will further help direct future research.

The other additional outcomes are revealing the inconsistencies between the disease count strategies used in each study, the most common methods of data collection, and the population size groupings of each paper.

Measures of effect

Please specify the effect measure(s) for you additional outcome(s) e.g. relative risks, odds ratios, risk difference‘ and/or 'number needed to treat.

This is a qualitative review so there are no measures for my outcomes.

26. * Data extraction (selection and coding).

Describe how studies will be selected for inclusion. State what data will be extracted or obtained. State how this will be done and recorded.

- The screening process will be conducted through the Covidence software. Two authors will screen through titles, abstracts, and full papers in accordance with the predetermined inclusion and exclusion criteria stated above. Any disagreements in the screening process will be resolved through a third ‘reviewer’s involvement to reach a consensus.
- Papers will be primarily screened and either included or excluded depending on whether they contain these basic factors: i) study looks at a multimorbid population and defines multimorbidity, ii) a specific clustering technique was defined, iii) study reports on the specific clusters of disease (does not just provide vague a count of how many clusters they have found)
- Raw data from the included articles will be extracted and organized by one person into a Microsoft Excel spreadsheet with the following headers:

1. Title of Article
2. The Type of Study (cross-sectional or cohort)
3. Multimorbid Population Investigated (paying extra attention to Country, and Means of Data Collection)
4. Number of Participants Involved in Each Study
5. Measurement Tool Used
6. Number of Diseases Used for the Disease Count
7. Cluster Technique
8. Most Prevalent Clusters Identified - Listed
9. Total Number of Clusters Identified
10. Commentary on the Clusters that Arise in Different Populations Based on Age, Sex, socioeconomic Class.
11. [1 change]. * Risk of bias (quality) assessment.

State which characteristics of the studies will be assessed and/or any formal risk of bias/quality assessment tools that will be used.

All included studies will be critically appraised using the relevant CASP (cohort) and AXIS (cross-sectional) checklists designed to assess the methodological quality of different observational studies by two reviewers.

Differences in the assessments will be resolved by discussion with a third reviewer to reach a consensus.

1. [1 change]. * Strategy for data synthesis.

Describe the methods you plan to use to synthesise data. This **must not be generic text** but should be **specific to your review** and describe how the proposed approach will be applied to your data. If meta analysis is planned, describe the models to be used, methods to explore statistical heterogeneity, and software package to be used.

Review findings will be synthesised narratively, as it is anticipated that there will be differences in population studied, conditions examined to identify multimorbidity and methods of clustering. Overall outcome measures will be presented in tabular form, accompanied by detailed descriptions of review characteristics and quality assessments. Up to five of the most common clusters identified in each of the selected articles will undergo a comparison analysis to determine the top 10 clusters overall.

Each cluster will be placed under broad cluster groupings in an MS Excel chart. Broad cluster groupings will be manually pre-determined by one person via a rough cluster count based on prevalence.

The broad cluster groups will be organized from largest to smallest, depending on how many articles included each cluster. This will allow the researcher to gain an appreciation of what clusters were most prevalent overall, and seek out the top 10 clusters.

Researchers and clinicians will be able to identify which clustering techniques are most suitable for use with different data types and in different population settings

1. * Analysis of subgroups or subsets.

State any planned investigation of ‘subgroups’. Be clear and specific about which type of study or participant will be included in each group or covariate investigated. State the planned analytic approach.

Other aspects of the guiding questions will be synthesized via the primary table.

1. * Type and method of review.

Select the type of review, review method and health area from the lists below.

Type of review

Cost effectiveness

No

Diagnostic

No

Epidemiologic

No

Individual patient data (IPD) meta-analysis

No

Intervention

No

Living systematic review

No

Meta-analysis

No

Methodology

No

Narrative synthesis

No

Network meta-analysis

No

Pre-clinical

No

Prevention

No

Prognostic

No

Prospective meta-analysis (PMA)

No

Review of reviews

No

Service delivery

No

Synthesis of qualitative studies

No

Systematic review

Yes

Other

No

Health area of the review

Alcohol/substance misuse/abuse

No

Blood and immune system

No

Cancer

No

Cardiovascular

No

Care of the elderly

No

Child health

No

Complementary therapies

No

COVID-19

No

Crime and justice

No

Dental

No

Digestive system

No

Ear, nose and throat

No

Education

No

Endocrine and metabolic disorders

No

Eye disorders

No

General interest

No

Genetics

No

Health inequalities/health equity

No

Infections and infestations

No

International development

No

Mental health and behavioural conditions

No

Musculoskeletal

No

Neurological

No

Nursing

No

Obstetrics and gynaecology

No

Oral health

No

Palliative care

No

Perioperative care

No

Physiotherapy

No

Pregnancy and childbirth

No

Public health (including social determinants of health)

Yes

Rehabilitation

No

Respiratory disorders

No

Service delivery

No

Skin disorders

No

Social care

No

Surgery

No

Tropical Medicine

No

Urological

No

Wounds, injuries and accidents

No

Violence and abuse

No

1. Language.

Select each language individually to add it to the list below, use the bin icon to remove any added in error.

English

There is not an English language summary

1. * Country.

Select the country in which the review is being carried out. For multi-national collaborations select all the countries involved.

Scotland

1. Other registration details.

Name any other organisation where the systematic review title or protocol is registered (e.g. Campbell, or The Joanna Briggs Institute) together with any unique identification number assigned by them. If extracted data will be stored and made available through a repository such as the Systematic Review Data Repository (SRDR), details and a link should be included here. If none, leave blank.

1. Reference and/or URL for published protocol.

If the protocol for this review is published provide details (authors, title and journal details, preferably in Vancouver format)

Add web link to the published protocol.

Or, upload your published protocol here in pdf format. Note that the upload will be publicly accessible.

No I do not make this file publicly available until the review is complete

Please note that the information required in the PROSPERO registration form must be completed in full even if access to a protocol is given.

1. Dissemination plans.

Do you intend to publish the review on completion?

Yes

Give brief details of plans for communicating review findings.?

It is anticipated that, upon completion, this systematic review will be published in a high-impact open access journal and presented at relevant conferences.

1. Keywords.

Give words or phrases that best describe the review. Separate keywords with a semicolon or new line. Keywords help PROSPERO users find your review (keywords do not appear in the public record but are included in searches). Be as specific and precise as possible. Avoid acronyms and abbreviations unless these are in wide use.

Multimorbidity; Clustering Techniques; Disease Clusters; Latent Class Analysis

1. Details of any existing review of the same topic by the same authors.

If you are registering an update of an existing review give details of the earlier versions and include a full bibliographic reference, if available.

1. * Current review status.

Update review status when the review is completed and when it is published. New registrations must be ongoing so this field is not editable for initial submission.

Please provide anticipated publication date

Review Ongoing

39 [1 change]. Any additional information.

Provide any other information relevant to the registration of this review.

Changes have been made upon request, and resubmitted.

1. 40. Details of final report/publication(s) or preprints if available.

Leave empty until publication details are available OR you have a link to a preprint (NOTE: this field is not editable for initial submission). List authors, title and journal details preferably in Vancouver format.

Give the link to the published review or preprint.
