# Supplementary material for: Identifying clusters of multimorbid disease and differences by age, sex, and socioeconomic status: A systematic review
Source: PLoS One. 2025 Aug 22;20(8):e0329794. doi: 10.1371/journal.pone.0329794 (PMC12373218; doi:10.1371/journal.pone.0329794)
Supplement: S4 Appendix — (DOCX) [file pone.0329794.s004.docx]

### **Appendix 4. Database Search Terms and Results.**

| **Database** | **Search Terms** | **Results** |
| --- | --- | --- |
| **Ovid MEDLINE(R) ALL <1946 to March 14, 2024>** | 1            multimorb*.mp.              2            Multimorbidity/              3            multi-morbid*.mp.         4            (multiple chronic adj3 (condition* or disease* or illness*)).mp.              5            (concurrent chronic adj3 (condition* or disease* or illness*)).mp.              6            multiple comorbidit*.mp.           7            exp cluster analysis/      8            exp Factor Analysis, Statistical/  9            Principal Component Analysis/  10          cluster*.mp.      11          ((factor or correspondence or multicorrespondence or "multi-correspondence" or "multiple correspondence" or "log linear" or "log linear" or loglinear or "latent class") adj1 analys*).mp.   12          ("multiway frequenc*" or "multi-way frequenc*" or "principal component*").mp 13          exp prevalence/              14          exp association/              15          ("observed/expected" or "O/E").mp.      16          prevalence.mp. 831681 17          1 or 2 or 3 or 4 or 5 or 6 15269 18          7 or 8 or 9 or 10 or 11 or 12 or 13 or 14 or 15 or 16           19          17 and 18 | 4579 |
| **Embase <1974 to 2024 March 14 >** | 1              multimorb*.mp.              2            multiple chronic conditions/       3            multi-morbid*.mp.         4            (multiple chronic adj3 (condition* or disease* or illness*)).mp.              5            (concurrent chronic adj3 (condition* or disease* or illness*)).mp.              6            multiple comorbidit*.mp.           7            cluster analysis/              8            latent class analysis/      9            exp factor analysis/        10          exp principal component analysis/          11          cluster*.mp.      12          ((factor or correspondence or multicorrespondence or "multi-correspondence" or "multiple correspondence" or "log linear" or "log linear" or loglinear or "latent class") adj1 analys*).mp.   13          ("multiway frequenc*" or "multi-way frequenc*" or "principal component*").mp.         14          exp prevalence/              15          exp association/              16          ("observed/expected" or "O/E").mp.      17          prevalence.mp. 18          1 or 2 or 3 or 4 or 5 or 6 19          7 or 8 or 9 or 10 or 11 or 12 or 13 or 14 or 15 or 16 or 17              20          18 and 19 | 6903 |
| **Scopus** | TITLE-ABS-KEY ( ( multimorb*  OR  "multi-morbid*"  OR  "multiple chronic condition*"  OR  "multiple chronic disease*"  OR  "multiple chronic illness*"  OR  "concurrent chronic condition*"  OR  "concurrent chronic disease*"  OR  "concurrent chronic illness*" )  AND  ( cluster*  OR  "factor analysis"  OR  "correspondence analysis"  OR  "component analysis"  OR  "multicorrespondence analysis"  OR  "multi-correspondence analysis"  OR  "multiple correspondence analysis"  OR  "log linear analysis"  OR  "loglinear analysis"  OR  "latent class analysis"  OR  "multiway frequenc*"  OR  "multi-way frequenc*"  OR  "principal component*"  OR  prevalence  OR  "observed/expected"  OR  "O/E" ) ) | 4880 |
| **Web of Science** | multimorb* OR "multi-morbid*" OR "multiple chronic condition*" OR "multiple chronic disease*" OR "multiple chronic illness*" OR "concurrent chronic condition*" OR "concurrent chronic disease*" OR "concurrent chronic illness*" (Topic) and cluster* OR "factor analysis" or "correspondence analysis" or "component analysis" OR "multicorrespondence analysis" or "multi-correspondence analysis" or "multiple correspondence analysis" or "log linear analysis" or "loglinear analysis" or "latent class analysis" or "multiway frequenc*" or "multi-way frequenc*" or "principal component*" or prevalence OR "observed/expected" or "O/E" (Topic) | 6365 |
| **CINAHL** | ( (multimorb* OR "multi-morbid*" OR "multiple chronic condition*" OR "multiple chronic disease*" OR "multiple chronic illness*" OR "concurrent chronic condition*" OR "concurrent chronic disease*" OR "concurrent chronic illness*") ) AND ( (cluster* OR "factor analysis" or "correspondence analysis" or "component analysis" OR "multicorrespondence analysis" or "multi-correspondence analysis" or "multiple correspondence analysis" or "log linear analysis" or "loglinear analysis" or "latent class analysis" or "multiway frequenc*" or "multi-way frequenc*" or "principal component*" or prevalence OR "observed/expected" or "O/E") ) | 1504 |
|  | Total: | 24231 |
|  | Total (excluding Figshare): | **23914** |
