# Supplementary material for: Identifying clusters of multimorbid disease and differences by age, sex, and socioeconomic status: A systematic review
Source: PLoS One. 2025 Aug 22;20(8):e0329794. doi: 10.1371/journal.pone.0329794 (PMC12373218; doi:10.1371/journal.pone.0329794)
Supplement: S1 Table — (DOCX) [file pone.0329794.s005.docx]

**Supplementary Table 1. Data Extraction –Study Characteristics.**

| **Paper #** | **Study** | **Study Type** | **Country**  **and year** | **Multi-morbid Population** | **Age Group** | **Number of People** | **Diagnostic Classification Tool     *(eg: ICD, ICPC codes)*** | **Disease Count Index    *(eg: Elixahauster, Barnett, Charlson’ O'Halleran)*** | **Number of Diseases in Disease Count Strategy** | **Cluster Technique** | **Can each person be in only one cluster?     *y/n*** | **# of Clusters Identified** | **Is there a priori decision to look for a set number of clusters?    *y/n*** |
| --- | --- | --- | --- | --- | --- | --- | --- | --- | --- | --- | --- | --- | --- |
| 1 | Forslund et al. 2021 [1] | Cohort | Sweden    2017 | General population | 18+ | 2323667 | ICD10    Database - Primary Care, Outpatients, Inpatients | Barnett | 40 | K-means clustering | Y | 7 | Y |
| 2 | Wang et al. 2020 [2] | Cohort | China    2011-2015 | Working & retired workers | 45+ | 2097150 | ICD10    Database - Outpatients, Inpatients | Self-selected | 13 | Association rule mining     Hierarchical cluster analysis | ARM: Y   HCA: N | N/A | ARM: N  HCA: N |
| 5 | Violan et al. 2019  [3] | Cross-Sectional | Spain  2012 | General population | 65-99 | 916619 | ICD10     Database - Primary Care | Caldaron-Laranaga | 60 | Fuzzy c-means | N | 8 | N |
| 6 | Bisquera et al. 2021 (daisy-chained) [4] | Cross-Sectional | UK  2005-2020 | General population | 18+ | 826926 | Read  SNOMED.    Database -  Primary care | Based on QOF but self selected | 32 | Multiple Correspondence Analysis | N | 5 | N |
| 9 | Moller et al. 2020 [5] | Cohort | Denmark  2017 | Random sample of population | 16+ | 470794 | ICD10, prescribing.    Database - Secondary, psychiatric care, prescribing | Self selected | 47 | Latent class analysis | Y | 7 for 65+, 7 for 45-64, 5 for 16-44 | N |
| 10 | Roso-Llorach et al. 2018 [6] | Cross-Sectional | Spain  2010 | General population | 45-64 | 408994 | ICD-10     Database - Primary Care | ICD-10 disease categories | 79-women, 73-men | Hierarchical cluster analysis   Exploratory factor analysis | HCA: N    EFA: Y | HCA 12 for women, 15 for men.      EFA 9 for women, 10 for  men | HCA: N    EFA: N |
| 11 | Violan et al. 2018 [7] | Cross-Sectional | Spain  2010 | General population | 45-64 | 523656 | ICD-10     Database - Primary Care | Calinski Criterion | 241 | Multiple correspondence analysis   K-means clustering | MCA; N  K-means: Y | 6 for women, 6 for men | MCA; N  K-means: N |
| 12 | Zhu et al. 2019 [8] | Cohort | England  2012 | General population CPRD | 18+ | 113211 | Read code     Database - Primary Care | Barnett | 38 | LCA | Y | 18-44 years: 5 clusters     45-64 years: 5 clusters     65-84 years: 6 clusters | N |
| 14 | Foguet-Boreu et al. 2019 [9] | Cross-sectional study | Spain   2010 | General Population | 64+ | 322328 | ICD10    Database - Primary Care SIDIAP | self selected | 263 | Hierarchical cluster analysis | N | Female 65-79: 42 clusters    Male 65-79: 67 clusters    Female 80+: 85 clusters    Male 80+: 58 clusters | N |
| 15 | Guisado-Clavero et al. 2018 [10] | Cohort study | Spain   2009 | General population | 65+ | 190108 | ICD-10     Database - Primary Care’ | O'Halloran Criteria | 129 | Multiple correspondence analysis   AND  K-means clustering  Together | MCA; Y  K-means: Y | 6 per group | Y |
| 16 | Zheng et al. 2021 [11] | Cross-sectional study   Longitudinal mortality follow up | USA  2002-2015 | General Population | 50+ | 166126 | N/A     Self-Reported Survey - non-institutionalized population | US Department of Health and Human Services (DHHS) | 13 | Latent class analysis | Y | 5 | N |
| 17 | Larsen et al. 2017 [12] | Cross-sectional study | Denmark  2013 | General population | 16+ | 162283 | N/A     Self-reported Survey - General population | Based on WHO but self-selected | 15 | Latent class analysis | Y | 7 | N |
| 18 | Schafer et al. 2010 [13] | Cross-sectional study | Germany   2006 | Statutory Insured Population | 65+ | 149280 | ICD-10    Insurance claims Database – People economically active currently insured during 2006 | ADT-Panel | 46 | Exploratory tetrachoric factor analysis | N | 3 for males    3 for females | N |
| 19 | Juul-Larsen et al. 2020 [14] | Longitudinal cohort study | Denmark 2011 | General population | 65+ | 129900 | ICD-10  ATC-codes    Database - Secondary care national registers | The Chronic Condition Measurement Guide | 22 | Latent class analysis | Y | 8 | N |
| 21 | Holden et al. 2011  [15] | Cross-sectional study | Australia   2004-2005 | Working population | 30+ | 78000 | N/A    Self-reported survey | Based on WHO Health and Productivity Questionnaire but self selected | 23 | Exploratory factor analysis | N | 6 | N |
| 22 | Mino-Leon et al. 2017 [16] | Cross-sectional study | Mexico  2013 | Public health institution users | 60+ | 77573 | ICD-10    Database - Primary care | Cumulative Illness Rating Sale (CIRS) | 11 | Hierarchical cluster analysis | N | 5 | N |
| 23 | Matesanz- [17] | Retrospective cohort study | Spain  2000- 2015 | General population | –0 - 72 | 74220 | ICD-9-CM      Database - Secondary care | IANUS   CIRS Scale | 32 | Multiple correspondence analysis | N | 5 | Y |
| 24 | Bayes-Marin et al. 2020 [18] | Cohort study | 17 international cohorts  Three population-based studies, self-reported   1. English Longitudinal Study of Ageing  2. Survey of Health, Ageing and Retirement in Europe Study  3. Study on Global Ageing and Adult Health | General population | 50+ | 72140 | N/A    Self-reported Surveys | Maelstrom Research | 8 | Latent class analysis | Y | 3 | N |
| 25 | Hernandez et al. 2021 [19] | Cross-sectional study | US Health and Retirement Study (n = 10,858)  Canadian Longitudinal Study on Ageing (n = 36,647)  English Longitudinal Study of Ageing (n = 7938)  Irish Longitudinal Study on Ageing (n = 6668)  2012    Self-reported | Retired population | 52+ | 62111 | N/A    Self-reported Surveys | Self-select–d - Selected the  self-reported conditions that were common across all four studies | 10 | Latent class analysis | Y | 5 | N |
| 26 | De Carvalho et al. 2018 [20] | Cross-sectional study | Brazil  Population-based survey   2013 National Health Survey | General population | Adults (18+) | 60202 | N/A    Self-reported Surveys | Self selected | 14 | Hierarchical cluster analysis | N | 4 | N |
| 27 | Garin 2016 (Daisy-Chained) [21] | Cross-sectional study | Collaborative Research on Ageing in Europe project (Finland, Poland, and Spain) and the World Health Organization’s Study on Global Ageing and Adult Health (China, Ghana, India, Mexico, Russia, and South Africa).    COURAGE and SAGE studies | Noninstitutionalized adults | 50+ | 41909 | N/A    Self-reported Surveys | Kaiser-Guttman et al. | 12 | Exploratory factor analysis | Y | 6 | N |
| 28 | Lai et al. 2021 [22] | Cross-sectional study | Zurich and Hong Kong    2010-2013 (HK)  2009-2017 (Zurich) | randomly selected discharged multimorbid inpatients | 45+ | 20000 | ICD-9  ICD-10    Database - Secondary care | Jaccard index | 30 | Hierarchical clustering analysis | Y | 18 (9 clusters per country) | N |
| 29 | Yao et al. 2020 [23] | Cohort study | China  2011-2015 | General population | 50+ | 19841 | N/A    Self-reported survey | Self selected | 14 | Hierarchical cluster analysis | N | 4 | N |
| 30 | Quinones et al. 2021 [24] | Prospective cohort study | USA    19–8 - 2014 | noninstitutionalized older adults | 51+ | 17297 | N/A    Self-reported survey | Cigolle et al. | 8 | Latent class analysis | Y | 3 for 1988, 3 for 2014 | N |
| 32 | Ronaldson et al. [25] | Cohort study | England Longitudinal Study of Aging (ELSA) 2002 (ongoing) | General older population | 50+ | 11391 | N/A    Self-reported survey | Self selected | 14 | Latent class analysis | Y | 4 | N |
| 33 | Khorrami et al. 2019 [26] | Cross-sectional study | Iran    2011 | General population | 20-70 | 10069 | N/A    Self-reported survey | Based on WHO  STEPS Program but self selected | 12 | Latent class analysis | Y | 4 for males, 4 for females | N |
| 35 | Wang et al. 2017 [27] | Cross-sectional study | Australia     2007 | General population | Adults (16+) | 8841 | N/A    Self-reported survey | Based on ABS National Survey of Mental Health and Wellbeing and AIHW National Health Priority Areas but self selected | 11 | Hierarchical cluster analysis | N | 2 | N |
| 36 | Park et a. 2019 [28] | Cross-sectional study | Korea    2013–2015 | Noninstitutionalized adults | 50+ | 8370 | N/A    Self-reported survey | Based on KNHANES but self selected | 10 | Latent class analysis | Y | 3 | Y (up to 6) |
| 37 | Lu et al. 2021[29] | Cross-sectional study | China    2019 | Patients without cognitive impairment living in the selected residential building/villagers’ groups | 60+ | 7480 | N/A    Self-reported survey via face to face interview | Chronic diseases of health statistics yearbook in China 2019 | 17 | Latent class analysis | Y | 3 | N |
| 38 | Nguen et al. 2019 [30] | Cohort study | Boston, USA    2011 | Medicare beneficiaries | 65+ | 7197 | N/A    Self-reported survey | Self-selected | 10 | Latent class analysis | Y | 5 | N |
| 39 | Liu et al. 2021[31] | Cohort study | China     2011-2013-2015-2018 | General population | 60+ | 6634 | N/A    Self-reported survey | Self-selected | 14 | Latent class analysis | Y | 5 | N |
| 40 | Hernandez et al. 2019 [32] | Cross-sectional study | Ireland    Population based study | General population | 50+ | 6101 | N/A    Self-reported survey | Self-selected | 31 | Association rules | Y | 3 for males    2 for females | N |
| 41 | Olaya et al. 2017 [33] | Prospective cohort study | Spain     2011-2012 | non-institutionalized adults | 50+ | 3541 | N/A    Face-to-face interviews, self-reported | Based on WHO SAGE protocols but self selected | 11 | Latent class analysis | Y | 3 | N |
| 45 | Marengioni et al., 2013 [34] | Cross-sectional study | Italy     2008-2010 | General population | 65+ | 2791 | ICD-9    Database - Secondary care | Self selected (only selected diseases with a prevalence of > 5%) | 19 | Cluster analysis | N | 14 (7 in each wave) | N |
| 46 | Craig et al. 2021 [35] | Cross-sectional study | Jamaica    2007-2008 | General population | Adults (15+) | 2551 | N/A    Self-reported survey | Based on Diederichs et al. but self selected | 11 | Latent class analysis | Y | 4 | N |
| 47 | Gu et al. 2017 [36] | Cross-sectional study | Nanjing, China     2013 | General population | 60+ | 2452 | N/A    Face-to-face interviews, self-reported | Self-selected | 13 | Factor Analysis | Y | 3 | N |
| 49 | Prazeres et al. 2015 [37] | Cross-sectional, analytical study | Portugal    2013-2014 | General population | 18+ | 1993 S | ICPC-2     Primary care’ | O'Halloran Criteria | 147 | Cluster analysis | N | 6 | N |
| 50 | Chidumwa et al. 2021 [38] | Cross-sectional study | South Africa    20–4 - 2015   wave 2 | General population | 45+ | 1967 | N/A     Database - Primary care   And   Self-reported data | Self-selected | 7 | Latent class analysis | Y | 3 | N |
| 51 | Tan et al. 2015 [39] | Cross-sectional study | China (rural)    2010-2011 | General population | 60+ | 1480 | N/A     Database - Primary care   And   Self-reported data and Lab tests | Based on Marengoni et al. but self-selected | 16 | Exploratory factor analysis | Y | 2 | N |
| 52 | Held et al. 2016  [40] | Population-based cross-sectional study | Sydney, Australia | Community-dwelling   Male population only | 70+ | 1464 | N/A    Self-reported survey | Based on the Functional Comorbidity Index and Diedrichs et al. but self-selected | 17 | Association Rules Analysis | N | 5 | N |
| 53 | Costa et al. 2018 [41] | Cross-sectional population-based study | Brazil    2014 | General population | 60+ | 1451 | N/A    Self-reported survey | Self-selected | 29 | Factorial analysis | N | 3 | N |
| 54 | Marengoni et al. 2009 [42] | Community-based prospective cohort | Sweden    1987-1993 | General population | 15 | 1099 | ICD-9    Dataset - Physical examination | Based on Timmreck et al. but self-selected | 15 | Cluster analysis | N | 5 | N |
| 55 | Filipcic et al. 2018 [43] | Cross-sectional study | Zagreb, Croatia    2013-2015 | General population | Adults (18+) | 837 | N/A    Self-reported survey | EUROSTAT Methodological manual | 15 | Latent class analysis | Y | 4 | N |
| 56 | Bare et al. 2021 [44] | Multicentre, prospective cohort study | Spain    2016 -  2018 | Internal medicine or geriatric wards | 65+ | 740 | N/A    Dataset - Hospitalised patients with exacerbation - internal medicine or geriatric wards | Based on Salisbury 64 conditions  (includes  Charlson indices 19 conditions) but self-selected | 64 | fuzzy c-means | N | 4 | N |
| 58 | Juul-Larsen et al. 2020 [45] | Longitudinal prospective cohort study | Denmark    2012-2013 | Patients acutely admitted to the medical section of the Emergency Department | 65+ | 369 | N/A    Dataset - Secondary care | Juul-Larsen et al. | 22 | Latent class analysis | Y | 4 | N |
| 60 | Han et al. 2022 [46] | Cohort study | China     2018 | General older population | 65+ | 15275 | N/A    Face-to-face interviews, primary care | Previous CLHLS survey | 18 | Association Rules Mining | N | 10 | N |
| 61 | Gonsoulin et al. 2017 [47] | Cohort study | USA 2018 | Senior-aged women Veterans | 65+ | 38597 | N/A Database - Secondary care - Outpatient | Frayne et al. 2014 | 20 | Latent class analysis | Y | 6 | N |
| 62 | Roh et al. 2022 [48] | Cross-sectional study | Korea     2016 | General population | 19+ | 226709 | N/A    Self-reported survey | Based on Korean Community Health Survey 2016 | 7 | Association Rules Analysis | N | 2 in young adult, 18 in middle aged group, 30 in senior group | Y |
| 63 | Zhou et al. 2022 [49] | Cohort | China     2012 | General population | 45+ | 13613 | N/A    Database from nationally representative study | N/A | 16 | Latent class analysis | Y | 4 | N |
| 65 | Mucherino et al. 2021 [50] | Cross-sectional study | Spain     2011-1025 | General population | <65 | 1E+06 | ICPC and ICD-9    Database - Primary and Secondary care records | Based on Salisbury et al. and ACG syst–m - Johns Hopkins | 113 | Exploratory factor analysis | Y | 7 | N |
| 66 | Zhu et al. 2019 [51] | Cross-sectional study | UK    2012 | General population | 18+ | 391669 | N/A     Database - Primary and Secondary care | Barnett et al. and CPRD | 38 | Latent class analysis | Y | 20 across four age strata | N |
| 67 | Klinedinst et al. 2022 [52] | Cohort | USA    2011-205 | General older population | 65+ | 6179 | N/A    Self-reported survey | Self-selected | 11 | Latent class analysis | Y | 6 | N |
| 68 | Zheng et al. [53] | Cross-sectional study | USA    2010-2015 | General population | 18+ | 86745 | ICD-9    Self-reported survey | Hwang et al. | 23 | Latent class analysis | Y | 5 | N |
| 69 | Sibley et al. 2014 [54] | Cross-sectional study | Canada    2008-2009 | General older population | 65+ | 16357 | N/A    Self-reported survey | Self-selected | 8 | Hierarchical cluster analysis | N | 7 | N |
| 70 | Hajat et al. 2020 [55] | Cross-sectional study | USA    2015 | Population provided with Insurance | 18+ | 2E+06 | ICD-9    Database - Primary and Secondary care - claims-based | Based on AHRQ list | 69 | k-means clustering | Y | 10 | Y |
| 71 | Franti et al. 2022 [56] | Cohort | Finland     2015-2018 | General population | 18+ | 4E+06 | ICD-9 CM and ICPC-2    Database - Primary and Secondary care - claims-based | Marengoni et al. Wartelle et al. | 110 | k-means clustering | Y | 15 | Y |
| 72 | Garcia-Olmos et al. 2012 [57] | Cross-sectional study | Spain    2007 | General population | Adults (14+) | 198670 | N/A     Database - Primary care | Based o’ O'Halloran et al. but self-selected | 26 | Multiple Correspondence Analysis | Y | 4 | N |
| 73 | Collerton et al. 2016 [58] | Cohort | Uk    2006-2007 | General older population | 75+ | 710 | N/A    Database - Primary Care | Diedrichs et al. | 20 | Hierarchical cluster analysis | N | 5 | N |
| 75 | Dorenkamp et al. 2016 [59] | Cohort | Netherlands     2003 | General population | 55+ | 3386 | N/A    Self-reported survey | Based on Dutch Association of General Practitioners (LHV) | 15 | Agglomerative Hierarchical cluster analysis | N | 3 | N |
| 78 | Aoki et al. 2021 [60] | Cohort | Japan    2016-2017 | General population | 50+ | 1211 | N/A    Self-reported survey | Self-selected | 15 | Exploratory factor analysis | Y | 5 | N |
| 79 | Pati et al. 2022 [61] | Cross-sectional study | India    2015 | General population | 16-64 | 2912 | N/A    Self-reported survey | Self-selected | 18 | Latent class analysis | Y | 2 | N |
| 80 | Rzewuska et al. 2017 (PNS 2013) [62] | Cross-sectional study | Brazil    2013 | General population | 18+ | 60202 | N/A    Self-reported survey | Malta et al. | 14 | Exploratory tetrachoric factor analysis | Y | 3 | N |
| 81 | Carretero-Bravo et al. 2022 [63] | Cross-sectional study | Spain    2022 | General population | 50+ | 1592 | N/A    Self-reported survey | Based on European Health Survey | 32 | Latent class analysis | Y | 5 | N |
| 87 | Batista et al. 2022 [64] | Cross-sectional study | Brazil    2013 | General older population | 60+ | 11177 | N/A    Self-reported survey | Self-selected | 16 | Network analysis | N | 4 | N |
| 88 | Puri et al. 2022 [65] | Cross-sectional study | India    2017-2018 | General older population | 45+ | 58975 | N/A    Self-reported survey | Self-selected | 16 | Latent class analysis | Y | 6 | N |
| 89 | Whiston et al. 2016 [66] | Retrospective Cohort | USA     1999-2007 | Medicare beneficiaries | 65+ | 14052 | N/A    Self-reported survey, inpatients, emergency department | Self-selected | 13 | Latent class analysis | Y | 6 | N |
| 90 | Cigolle et al. 2012 [67] | Cohort | Spain    2011-2012 | non-institutionalized adults | 50+ | 3541 | N/A    Self-reported survey | Based on WHO-SAGE protocol | 11 | Latent class analysis | Y | 3 | N |
| 91 | Jackson et al. 2016 [68] | Cohort | Australia     2016 | Female Medicare beneficiaries only | 45+ | 4896 | N/A    Self-reported survey | Self-selected | 18 | Exploratory factor analysis | Y | 5 | N |
| 92 | Eyowas et al. 2022 [69] | Cross-sectional study | Ethiopia     2020 | General population | 40+ | 1440 | N/A    Database and self-reported survey- Secondary care, Outpatient medical care | Based on their own previous study | 8 | Latent class analysis | Y | 4 | N |
| 93 | Lind et al. 2020 [70] | Cohort | Australia    2014-2017 | General older population | 65+ | 9436 | N/A    Database - Residential aged care facility | Self-selected | 60 | Latent class analysis | Y | 7 | N |
| 97 | Islam et al. 2014 [71] | Cross-sectional study | Australia     2009 | General older population | 50+ | 10000 | N/A    Self-reported survey | Based on Australian Bureau of Statistics 2009,  and McRae et al. | 11 | Hierarchical cluster analysis     Latent Class Analysis | HCA  N    LCA  Y | 3 | HCA  N    LCA  N |
| 98 | Marventano et al. 2014 [72] | Cross-sectional study | Spain     2018 | General older population | 65+ | 2818 | N/A    Face-to-face interviews | Barthel Index | 11 | Exploratory factor analysis | N | 4 | N |
| 99 | Machon et al. 2020 [73] | Cross-sectional study | Spain     2016-2018 | General population | 70+ | 813 | ICD-9 and ICD-10    Database - Primary care | Calderon-Larranga et al. | 60 | Multiple Correspondence Analysis, K-means and Hierarchical Cluster Analysis | N | 3 robust clusters, 4 frail clusters | N |
| 100 | Gu et al. 2018 [74] | Cohort | China     2017 | General older population | 60+ | 411 | N/A    Telephone  interviews | Gu et al. | 13 | Exploratory factor analysis | Y | 3 | N |
| 101 | Deruaz-Luyet et al. 2017  [75] | Cross-sectional study | Switzerland     2015 | General population | 18+ | 888 | ICPC-2    Database - Primary care | Self-selected | 75 | Hierarchical cluster analysis | N | 4 | N |
| 102 | Wang et al. 2017 [76] | Cross-sectional study | China     2017 | General population | 60+ | 2705 | N/A    Self-reported survey | Self-selected | 17 | Exploratory factor analysis | Y | 5 | N |
| 103 | Wartelle et al. 2022 [77] | Cohort | France     2018-2019 | General population | 18+ | 120722 | ICD-10    Database - Secondary care - Emergency Department | Self-selected | 2890 | Hierarchical cluster analysis | N | 16 | N |
| 106 | Kuwornu et al. 2014 [78] | Cross-sectional study | Canada     2005 | General population | 18+ | 3284 | N/A    Self-reported survey | Based on statistics Canada but Self-selected | 15 | Latent class analysis | Y | N/A | N |
| 108 | Nunes et al. 2016 [79] | Cross-sectional study | Brazil     2012 | General population | 20+ | 2927 | N/A    Face-to-face interviews | Based on Oliveira et al. but self-selected | 11 | Exploratory factor analysis | Y | 2 | N |
| 110 | Ruiz et al. 2015 [80] | Cohort | UK    2012-2013 | General older population | 65+ | 3E+06 | ICD-10    Database - Secondary care | Charlson comorbidity score | 20 | Exploratory factor analysis | Y | 3 | N |
| 111 | Guo et al. 2021 [81] | Cohort | China     2015 | General older population | 60+ | 9786 | N/A    Self-reported survey | Self-selected | 14 | Association Rules Analysis | N | 3 | N |
| 112 | Yao et al. 2022 [82] | Cohort | China     2002-2018 | general population | 65+ | 13144 | N/A    Self-reported survey | Self-selected | 14 | Exploratory factor analysis | Y | 4 | N |
| 113 | Marengoni et al. 2021 [83] | Cohort | Sweden     2001-2004 | General population | 60+ | 2571 | ICD-10    Self-reported survey | Calderon-Larranga et al. | 60 | Fuzzy c-means | N | 6 | N |
| 114 | Zhang et al. 2022 [84] | Cohort | China     2018 | General older population | 60+ | 10479 | N/A    Self-reported survey | Based on Zhang et al. and Yao et al. but self-selected | 14 | Latent class analysis | Y | 5 | N |
| 115 | Fan et al. 2022 [85] | Cohort | China     2004-2008 | General Population | 30-79 | 512723 | N/A    Self-reported survey | Self-selected | 15 | Hierarchical cluster analysis | N | 4 | N |
| 116 | Rodreigues et al. 2022 [86] | Cohort | Brazil     2015-2016 | General older population | 50+ | 8807 | N/A    Self-reported survey, secondary care | Based on Nunes et al. but self-selected | 19 | Network Analysis | N | 5 | N |
| 118 | Liu et al. 2022 [87] | Cross-sectional study | China     2018 | General population | 60+ | 2604 | N/A    Database - primary care and Face-to-face interviews | Self-selected (based on prevalence) | 10 | Latent class analysis | Y | 3 | N |
| 119 | Zacarias-Pons et al. 2021 [88] | Cohort | Europe (Nation-wide)    2013, 2015, 2017 | General population | 50+ | 25931 | N/A    Self-reported survey | Self-selected | 15 | Latent class analysis | Y | 4 for males, 4 for females | N |
| 120 | Jackson et al. 2015  [89] | Cohort | Australia     2002-2011 | Female older population | 45+ | 7270 | N/A    Self-reported survey | Self-selected (based on prevalence) | 31 | Exploratory factor analysis | Y | 3 for women | N |
| 121 | Garin et al. 2014 [90] | Cross-sectional study | Spain     2011-2012 | General population | 50+ | 3625 | N/A    Face-to-face interviews | Based on WHO's Sage study and National Collaborating Centre for Chronic Conditions | 9 | Exploratory factor analysis | Y | 3 | N |
| 122 | Buja et al. 2018 [91] | Cohort | Italy     2012 | General older population | 65+ | 2691 | N/A    Database - ACG | Barnett et al. | 15 | Latent class analysis | Y | 5 | N |
| 123 | Clerencia-Sierra et al. 2015  [92] | Cross-sectional study | Spain     2011 | General older population | 65+ | 924 | N/A    Database - Secondary care | Based on Salisbury et al. | 115 | Exploratory factor analysis | Y | 4 total | N |
| 124 | Aoki et al. 2018 [93] | Cross-sectional study | Japan    2016 | General population | 18-84 | 3307 | N/A    Self-reported survey | Based on Prados-Torres and Calderon-Larranga et al. | 17 | Factor analysis | Y | 5 | N |
| 125 | Ioakeim-Skoufa et al. 2020 [94] | Cross-sectional study | Spain    2011 | General population | 18+ | 1E+06 | ICPC and ICD-9    Database - Primary and Secondary care | Based on Johns Hopkins Adjusted Clinical Groups and Salisbury et al. | 114 | Exploratory factor analysis | Y | 12 in men, 12 in women | N |
| 126 | Dong et al. 2013 [95] | Cross-sectional study | Sweden    2007-2008 | General older  population | 85+ | 496 | ICD-10    Database - Primary and Secondary care and self-reported survey | Self-selected | 13 | Hierarchical cluster analysis | N | 5 for men, 5 for women | N |
| 127 | Zador et al. 2019 [96] | Cohort | US     2001-2012 | General population | Adults (16+) | 36390 | ICD-9 and ICD-10    Database - Secondary care - Intensive care | Elixhauser | 30 | Latent class analysis | Y | 6 | N |
| 129 | Simones et al. 2016  [97] | Cross-sectional study | Portugal     2005-2006 | General population | 18+ | 23754 | N/A    Self-reported survey | Based on OASH Criteria - Goodman et al. but self-selected | 11 | Latent class analysis | Y | 4 | N |
| 130 | John et al. 2003  [98] | Cross-sectional study | US    2003 | American Indian Elders | 60+ | 1039 | N/A    Self-reported survey | Self-selected | 11 | Cluster analysis | Y | 4 | N |
| 131 | Zheng et al. 2019 [99] | Cross-sectional study | US    2002-2014 | Noninstitutionalized adults | 18+ | 387780 | N/A    Face-to-face interviews | Based on Goodman et al. but self-selected | 13 | Latent class analysis | Y | 4 | N |
| 132 | Formiga et al. 2013 [100] | Cross-sectional study | Spain    2015 | General older population | 85+ | 328 | N/A    Face-to-face interviews | Based on Charlson Index but Self-selected | 16 | Hierarchical cluster analysis | N | 4 | N |
| 133 | Teh et al. 2018  [101] | Cohort | New Zealand     2010 | General older population - Māori and non-Māori | 80+ | 937 | N/A    Database - Primary and Secondary care and self-reported survey | Based on Tet et al. but self-selected | 14 | Hierarchical clustering analysis | N | 6 for Māori and 6 for non-Māori | N |
| 135 | Piotrowicz et al. 2021 [102] | Cross-sectional study | Poland    2007-2011 | General older  population | 55+ | 4588 | N/A    Self-reported survey | Self-selected | 17 | Factor analysis | Y | 4 for 55-59 years, 5 for 65-79 years, 5 for 80+ years | N |
| 137 | Juul-Larsen et al. 2018 [103] | Cohort | Denmark    2011 | General population | 65+ | 129900 | ICD-10 and ATC codes    Database - Secondary care | Chronic Condition Measurement Guide | 22 | Latent class analysis | Y | 8 for Males, 8 for Females | N |
| 138 | Kirchberger et al. 2012 [104] | Cross-sectional study | Germany    19984-2001 | General older  population | 65+ | 4127 | N/A    Face-to-face interviews and self-reported survey | Charlson Comorbidity Index | 13 | Exploratory tetrachoric factor analysis | N | 4 | N |
| 142 | Tan et al. 2020 [105] | Cross-sectional study | Singapore    2015-2016 | General population | 18+ | 437849 | ICD-10    Database - Primary care | Self-selected | 93 | Exploratory factor analysis | Y | 7 | N |
| 145 | Kshatri et al. 2020 [106] | Cross-sectional study | India     2011 | General older  population | 60+ | 725 | N/A    Database - Census data | Self-selected | 18 | k-means clustering | Y | 3 | Y |
| 146 | Hunter et al. 2021 [107] | Cross-sectional study | Australia     2010-2015 | General population | 59+ | 5029 | N/A    Self-reported survey and on-site clinical assessments | Self-selected | 21 | Latent class analysis | Y | 4 | N |
| 147 | Lin et al. 2022 [108] | Cross-sectional study | China    2020 | General older  population | 65+ | 31708 | ICD codes    Database - Primary care | Self-selected | 7 | Association rule mining | Y | 4 | N |
| 148 | Drajovic et al. 2016 [109] | Cross-sectional study | Serbia     2013 | General population | 20+ | 13103 | N/A    Self-reported survey | Based on Holden et al. and Schafer et al. but self-selected | 12 | Exploratory factor analysis | Y | 6 in total | N |
| 149 | Craig et al. 2020 [110] | Cross-sectional study | Jamaica     2007-2008 | General population | Adults (15-74) | 2551 | N/A    Self-reported survey | Based on Diederichs et al. but self-reported | 11 | Latent class analysis  and   Exploratory factor analysis | Y for both | 4 for LCA,  3 for EFA | N for both |
| 152 | Poblador-Plou et al. 2014 [111] | Cohort | Spain and Netherlands    2008, 2010 | General population | Adults (14+) | 158035 | ICPC    Database - Primary care | Based on Salisbury et al. but self-selected | 260 | Exploratory factor analysis | Y | 5 overall | N |
| 153 | Ibarra-Castillo et al. 2018 [112] | Cohort | Spain    2009-2014 | General population | 65+ | 190108 | ICD-10 and ICPC-2     Database -Electronic Health Records | Based o’ O'Halloran Criteria but self-selected | 147 | Cluster analysis | Y | 7 | N |
| 154 | Tang et al. 2020 [113] | Cross-sectional study | Denmark    2015-2019 | General population | 18+ | 10781 | N/A    Self-reported survey | Based on Danish General Suburban Population Study and National Health Cohort in Denmark | 18 | Factor analysis | N | 2 | N |
| 156 | Hsu et al. 2015 [114] | Cohort | Taiwan    1993-2007 | General population | 60+ | 2584 | N/A    Face-to-face interviews | Self-selected | 6 | Multiple Trajectory Model | Y | 4 | N |
| 157 | Ho et al. 2022 [115] | Cohort | Taiwan    1996-2011 | General population | 50+ | 5130 | N/A    Self-reported survey | Self-selected | 12 | Latent class analysis | Y | 4 | N |
| 158 | Grant et al. 2020 [116] | Cohort | US    2018-2019 | General population | 18+ | 103869 | N/A     Database - Secondary care | Self-selected | 97 | Latent class analysis and K-means clustering | Y for both | 7 for LCA, 8 for K-means | N for both |
| 159 | Prenovost et al. 2018 [117] | Cohort | US    2014 | US  Veterans Health Administration Patients | 18+ | 68400 | ICD-9     Database - Secondary care - Inpatient, Outpatient, VHA and VA community care claims | Self-selected | 31 | Latent class analysis | Y | 6 | N |
| 160 | Zhong et al. 2023 [118] | longitudinal cross-sectional study | China     2018 | community | 45+ | 19599 | N/A    self-reported | self-selected | 14 | LCA | Y | 4 | N |
| 161 | Amirzada et al. 2023 [119] | cross-sectional study | Germany     2017 | community | 43-92 | 6554 | Self-selected, based on Federal Health Survey     self-reported and interview | Charlson et al. | 13 | LCA | Y | 5 | N |
| 162 | Roomaney et al. 2022 [120] | cross-sectional study | South Africa     2016 | Community | 15+ | 2368 | N/A    self-reported | self-selected | 9 | LCA | Y | 4 | N |
| 163 | Chen et al. 2023 [121] | cross-sectional study | China     2019 | Community | 60+ | 214 | N/A     secondary care (long-term care) database | self-selected | 9 | Network analysis and two-step cluster analysis | Y | 4 | N |
| 164 | Ioakeim-Skoufa et al. 2022 [122] | cohort | Spain    2010-2019 | community | 80+ | 91,442 | ICPC-1, ICD-9CM, CCS    primary care database | self-selected | 226 | K-means clustering | Y | N/A | Y |
| 165 | Zhong et al. 2022 [123] | longitudinal cross-sectional study | China  2018 | community | 45+ | 19599 | N/A    self-reported, national database | self-selected | 14 | LCA | Y | 4 | N |
| 166 | Zheng et al. 2020 [124] | cross-sectional study | USA   2010-2015 | community | 18+ | 86745 | ICD-9, CCS    national database, interviews | Goodman et al. | 23 | LCA | Y | 5 | N |
| 167 | Fagbamigbe et al. 2023 [125] | [cross-sectional study](http://0.0.0.16/) | UK  2000-2018 | community | 25+ | 318,235 | ICD-10    secondary care and outpatient database | Elixhauser Comorbidity Index | 31 | Market basket analysis | Y | 5 | N |

*Footnote: Clustering Technique Abbreviations*

*LCA - Latent class analysis; HCA - Hierarchical cluster analysis, MCA - Multiple correspondence analysis; EFA - Exploratory factor analysis*

**Supplementary Table Citations**

1. Forslund T, Carlsson AC, Ljunggren G, Arnlov J, Wachtler C. Patterns of multimorbidity and pharmacotherapy: a total population cross-sectional study. Fam Pract. 2021;38(2):132-40.

2. Wang X, Yao S, Wang M, Cao G, Chen Z, Huang Z, et al. Multimorbidity among Two Million Adults in China. Int J Environ Res Public Health. 2020;17(10):13.

3. Violan C, Foguet-Boreu Q, Fernandez-Bertolin S, Guisado-Clavero M, Cabrera-Bean M, Formiga F, et al. Soft clustering using real-world data for the identification of multimorbidity patterns in an elderly population: cross-sectional study in a Mediterranean population. BMJ Open. 2019;9(8):e029594.

4. Bisquera A, Gulliford M, Dodhia H, Ledwaba-Chapman L, Durbaba S, Soley-Bori M, et al. Identifying longitudinal clusters of multimorbidity in an urban setting: A population-based cross-sectional study. Lancet Reg Health Eur. 2021;3:100047.

5. Moller SP, Laursen B, Johannesen CK, Tolstrup JS, Schramm S. Patterns of multimorbidity and demographic profile of latent classes in a Danish population-A register-based study. PLoS ONE. 2020;15(8):e0237375.

6. Roso-Llorach A, Violan C, Foguet-Boreu Q, Rodriguez-Blanco T, Pons-Vigues M, Pujol-Ribera E, et al. Comparative analysis of methods for identifying multimorbidity patterns: a study of 'real-world' data. BMJ Open. 2018;8(3):e018986.

7. Violan C, Roso-Llorach A, Foguet-Boreu Q, Guisado-Clavero M, Pons-Vigues M, Pujol-Ribera E, et al. Multimorbidity patterns with K-means nonhierarchical cluster analysis. BMC Family Practice. 2018;19(1):108.

8. Zhu Y, Edwards D, Mant J, Payne RA, Kiddle S. Characteristics, service use and mortality of clusters of multimorbid patients in England: a population-based study. BMC Med. 2020;18(1):78.

9. Foguet-Boreu Q, Violan C, Rodriguez-Blanco T, Roso-Llorach A, Pons-Vigues M, Pujol-Ribera E, et al. Multimorbidity Patterns in Elderly Primary Health Care Patients in a South Mediterranean European Region: A Cluster Analysis. PLoS ONE. 2015;10(11):e0141155.

10. Guisado-Clavero M, Roso-Llorach A, Lopez-Jimenez T, Pons-Vigues M, Foguet-Boreu Q, Munoz MA, et al. Multimorbidity patterns in the elderly: a prospective cohort study with cluster analysis. BMC geriatr. 2018;18(1):16.

11. Diane Zheng D, Loewenstein DA, Christ SL, Feaster DJ, Lam BL, McCollister KE, et al. Multimorbidity patterns and their relationship to mortality in the US older adult population. PLoS ONE. 2021;16(1 January) (no pagination).

12. Larsen FB, Pedersen MH, Friis K, Glumer C, Lasgaard M. A Latent Class Analysis of Multimorbidity and the Relationship to Socio-Demographic Factors and Health-Related Quality of Life. A National Population-Based Study of 162,283 Danish Adults. PLoS ONE. 2017;12(1):e0169426.

13. Schafer I, von Leitner EC, Schon G, Koller D, Hansen H, Kolonko T, et al. Multimorbidity patterns in the elderly: a new approach of disease clustering identifies complex interrelations between chronic conditions. PLoS ONE. 2010;5(12):e15941.

14. Juul-Larsen HG, Andersen O, Bandholm T, Bodilsen AC, Kallemose T, Jorgensen LM, et al. Differences in function and recovery profiles between patterns of multimorbidity among older medical patients the first year after an acute admission-An exploratory latent class analysis. Arch Gerontol Geriatr. 2020;86:103956.

15. Holden L, Scuffham PA, Hilton MF, Muspratt A, Ng SK, Whiteford HA. Patterns of multimorbidity in working Australians. Popul Health Metr. 2011;9(1):15.

16. Mino-León D, Reyes-Morales H, Doubova SV, Pérez-Cuevas R, Giraldo-Rodríguez L, Agudelo-Botero M. Multimorbidity Patterns in Older Adults: An Approach to the Complex Interrelationships Among Chronic Diseases. Arch Med Res. 2017 Jan;48(1):121-127. doi: 10.1016/j.arcmed.2017.03.001. PMID: 28577866.

17. Matesanz-Fernandez M, Seoane-Pillado T, Iniguez-Vazquez I, Suarez-Gil R, Pertega-Diaz S, Casariego-Vales E. Description of multimorbidity clusters of admitted patients in medical departments of a general hospital. Postgraduate Medical Journal. 2022;98(1158):294-9.

18. Bayes-Marin I, Sanchez-Niubo A, Egea-Cortes L, Nguyen H, Prina M, Fernandez D, et al. Multimorbidity patterns in low-middle and high income regions: a multiregion latent class analysis using ATHLOS harmonised cohorts. BMJ Open. 2020;10(7):e034441.

19. Hernandez B, Voll S, Lewis NA, McCrory C, White A, Stirland L, et al. Comparisons of disease cluster patterns, prevalence and health factors in the USA, Canada, England and Ireland. BMC Public Health. 2021;21(1):1674.

20. de Carvalho JN, de Camargo Cancela M, de Souza DLB. Lifestyle factors and high body mass index are associated with different multimorbidity clusters in the Brazilian population. PLoS ONE. 2018;13(11):e0207649.

21. Garin N, Koyanagi A, Chatterji S, Tyrovolas S, Olaya B, Leonardi M, et al. Global Multimorbidity Patterns: A Cross-Sectional, Population-Based, Multi-Country Study. J Gerontol A Biol Sci Med Sci. 2016;71(2):205-14.

22. Lai FTT, Beeler PE, Yip BHK, Cheetham M, Chau PYK, Chung RY, et al. Comparing Multimorbidity Patterns Among Discharged Middle-Aged and Older Inpatients Between Hong Kong and Zurich: A Hierarchical Agglomerative Clustering Analysis of Routine Hospital Records. Front Med (Lausanne). 2021;8:651925.

23. Yao SS, Cao GY, Han L, Chen ZS, Huang ZT, Gong P, et al. Prevalence and Patterns of Multimorbidity in a Nationally Representative Sample of Older Chinese: Results From the China Health and Retirement Longitudinal Study. J Gerontol A Biol Sci Med Sci. 2020;75(10):1974-80.

24. Quinones AR, Newsom JT, Elman MR, Markwardt S, Nagel CL, Dorr DA, et al. Racial and Ethnic Differences in Multimorbidity Changes Over Time. Med Care. 2021;59(5):402-9.

25. Ronaldson A, Arias de la Torre J, Bendayan R, Yadegarfar ME, Rhead R, Douiri A, et al. Physical multimorbidity, depressive symptoms, and social participation in adults over 50 years of age: findings from the English Longitudinal Study of Ageing. Aging Ment Health. 2022:1-11.

26. Khorrami Z, Rezapour M, Etemad K, Yarahmadi S, Khodakarim S, Mahdavi Hezaveh A, et al. The patterns of Non-communicable disease Multimorbidity in Iran: A Multilevel Analysis. Sci. 2020;10(1):3034.

27. Wang L, Palmer AJ, Cocker F, Sanderson K. Multimorbidity and health-related quality of life (HRQoL) in a nationally representative population sample: implications of count versus cluster method for defining multimorbidity on HRQoL. Health Qual Life Outcomes. 2017;15(1):7.

28. Park B, Lee HA, Park H. Use of latent class analysis to identify multimorbidity patterns and associated factors in Korean adults aged 50 years and older. PLoS ONE. 2019;14(11):e0216259.

29. Lu J, Wang Y, Hou L, Zuo Z, Zhang N, Wei A. Multimorbidity patterns in old adults and their associated multi-layered factors: a cross-sectional study. BMC geriatr. 2021;21(1):372.

30. Nguyen QD, Wu C, Odden MC, Kim DH. Multimorbidity Patterns, Frailty, and Survival in Community-Dwelling Older Adults. J Gerontol A Biol Sci Med Sci. 2019;74(8):1265-70.

31. Liu H, Zhang X, Chen B, Fang B, Lou VWQ, Hu J. The Differential Impact of Multimorbidity Patterns and Subsequent Accumulation on Longitudinal Trajectories of Physical Function Decline in a Population-Based Cohort of Older People. J Gerontol A Biol Sci Med Sci. 2022;77(8):1629-36.

32. Hernandez B, Reilly RB, Kenny RA. Investigation of multimorbidity and prevalent disease combinations in older Irish adults using network analysis and association rules. Sci. 2019;9(1):14567.

33. Olaya B, Moneta MV, Caballero FF, Tyrovolas S, Bayes I, Ayuso-Mateos JL, et al. Latent class analysis of multimorbidity patterns and associated outcomes in Spanish older adults: a prospective cohort study. BMC geriatr. 2017;17(1):186.

34. Marengoni A, Nobili A, Pirali C, Tettamanti M, Pasina L, Salerno F, et al. Comparison of disease clusters in two elderly populations hospitalized in 2008 and 2010. Gerontology. 2013;59(4):307-15.

35. Craig LS, Cunningham-Myrie CA, Hotchkiss DR, Hernandez JH, Gustat J, Theall KP. Social determinants of multimorbidity in Jamaica: application of latent class analysis in a cross-sectional study. BMC Public Health. 2021;21(1):1197.

36. Gu J, Chao J, Chen W, Xu H, Wu Z, Chen H, et al. Multimorbidity in the community-dwelling elderly in urban China. Arch Gerontol Geriatr. 2017;68:62-7.

37. Prazeres F, Santiago L. Prevalence of multimorbidity in the adult population attending primary care in Portugal: a cross-sectional study. BMJ Open. 2015;5(9):e009287.

38. Chidumwa G, Maposa I, Corso B, Minicuci N, Kowal P, Micklesfield LK, et al. Identifying co-occurrence and clustering of chronic diseases using latent class analysis: cross-sectional findings from SAGE South Africa Wave 2. BMJ Open. 2021;11(1):e041604.

39. Wang R, Yan Z, Liang Y, Tan EC, Cai C, Jiang H, et al. Prevalence and Patterns of Chronic Disease Pairs and Multimorbidity among Older Chinese Adults Living in a Rural Area. PLoS ONE. 2015;10(9):e0138521.

40. Held FP, Blyth F, Gnjidic D, Hirani V, Naganathan V, Waite LM, et al. Association Rules Analysis of Comorbidity and Multimorbidity: The Concord Health and Aging in Men Project. J Gerontol A Biol Sci Med Sci. 2016;71(5):625-31.

41. Costa CDS, Flores TR, Wendt A, Neves RG, Tomasi E, Cesar JA, et al. Inequalities in multimorbidity among elderly: a population-based study in a city in Southern Brazil. Cad Saude Publica. 2018;34(11):e00040718.

42. Marengoni A, Rizzuto D, Wang H-X, Winblad B, Fratiglioni L. Patterns of Chronic Multimorbidity in the Elderly Population. J Am Geriatr Soc. 2009;57(2):225-30.

43. Filipcic I, Filipcic IS, Grosic V, Bakija I, Sago D, Benjak T, et al. Patterns of chronic physical multimorbidity in psychiatric and general population. J Psychosom Res. 2018;114:72-80.

44. Bare M, Herranz S, Roso-Llorach A, Jordana R, Violan C, Lleal M, et al. Multimorbidity patterns of chronic conditions and geriatric syndromes in older patients from the MoPIM multicentre cohort study. BMJ Open. 2021;11(11) (no pagination).

45. Juul-Larsen HG, Andersen O, Bandholm T, Bodilsen AC, Kallemose T, Jorgensen LM, et al. Differences in function and recovery profiles between patterns of multimorbidity among older medical patients the first year after an acute admission-An exploratory latent class analysis. Arch Gerontol Geriatr. 2020;86:103956.

46. Han S, Mo G, Gao T, Sun Q, Liu H, Zhang M. Age, sex, residence, and region-specific differences in prevalence and patterns of multimorbidity among older Chinese: evidence from Chinese Longitudinal Healthy Longevity Survey. BMC Public Health. 2022;22(1):1116.

47. Gonsoulin ME, Durazo-Arvizu RA, Goldstein KM, Cao G, Zhang Q, Ramanathan D, et al. A Health Profile of Senior-Aged Women Veterans: A Latent Class Analysis of Condition Clusters. Innov Aging. 2017;1(2):01.

48. Roh EH. Analysis of multiple chronic disease characteristics in South Koreans by age groups using association rules analysis. Health Inform J. 2022;28(1):14604582211070208.

49. Zhou J, Wei MY, Zhang J, Liu H, Wu C. Association of multimorbidity patterns with incident disability and recovery of independence among middle-aged and older adults. Age Ageing. 2022;51(8):02.

50. Mucherino S, Gimeno-Miguel A, Carmona-Pirez J, Gonzalez-Rubio F, Ioakeim-Skoufa I, Moreno-Juste A, et al. Changes in Multimorbidity and Polypharmacy Patterns in Young and Adult Population over a 4-Year Period: A 2011-2015 Comparison Using Real-World Data. Int J Environ Res Public Health. 2021;18(9):21.

51. Zhu Y, Edwards D, Mant J, Payne RA, Kiddle S. Characteristics, service use and mortality of clusters of multimorbid patients in England: a population-based study. BMC Med. 2020;18(1):78.

52. Klinedinst TC, Terhorst L, Rodakowski J. Chronic condition clusters and associated disability over time. J Multimorb Comorb. 2022;12:26335565221093569.

53. Zheng DD, McCollister KE, Christ SL, Lam BL, Feaster DJ, Lee DJ. Chronic condition patterns in the US population and their association with health related quality of life. Prev Med. 2020;136:106102.

54. Sibley KM, Voth J, Munce SE, Straus SE, Jaglal SB. Chronic disease and falls in community-dwelling Canadians over 65 years old: a population-based study exploring associations with number and pattern of chronic conditions. BMC geriatr. 2014;14:22.

55. Hajat C, Siegal Y, Adler-Waxman A. Clustering and Healthcare Costs With Multiple Chronic Conditions in a US Study. Front. 2020;8:607528.

56. Franti P, Sieranoja S, Wikstrom K, Laatikainen T. Clustering Diagnoses From 58 Million Patient Visits in Finland Between 2015 and 2018. JMIR Med Inform. 2022;10(5):e35422.

57. Garcia-Olmos L, Salvador CH, Alberquilla A, Lora D, Carmona M, Garcia-Sagredo P, et al. Comorbidity patterns in patients with chronic diseases in general practice. PLoS ONE. 2012;7(2):e32141.

58. Collerton J, Jagger C, Yadegarfar ME, Davies K, Parker SG, Robinson L, et al. Deconstructing Complex Multimorbidity in the Very Old: Findings from the Newcastle 85+ Study. Biomed Res Int. 2016;2016:8745670.

59. Dorenkamp S, Mesters I, Schepers J, Vos R, van den Akker M, Teijink J, et al. Disease Combinations Associated with Physical Activity Identified: The SMILE Cohort Study. Biomed Res Int. 2016;2016:9053578.

60. Aoki T, Fukuhara S, Fujinuma Y, Yamamoto Y. Effect of multimorbidity patterns on the decline in health-related quality of life: a nationwide prospective cohort study in Japan. BMJ Open. 2021;11(6):e047812.

61. Pati S, Puri P, Gupta P, Panda M, Mahapatra P. Emerging multimorbidity patterns and their links with selected health outcomes in a working-age population group. J. 2022;63(1):E152-E60.

62. Rzewuska M, de Azevedo-Marques JM, Coxon D, Zanetti ML, Zanetti AC, Franco LJ, et al. Epidemiology of multimorbidity within the Brazilian adult general population: Evidence from the 2013 National Health Survey (PNS 2013). PLoS ONE. 2017;12(2):e0171813.

63. Carretero-Bravo J, Ramos-Fiol B, Ortega-Martín E, Suárez-Lledó V, Salazar A, O'Ferrall-González C, et al. Multimorbidity Patterns and Their Association with Social Determinants, Mental and Physical Health during the COVID-19 Pandemic. Int J Environ Res Public Health. 2022;19(24).

64. Batista SR, Sousa ALL, Nunes BP, Silva RR, Jardim P, Brazilian Group of Studies on M. Identifying multimorbidity clusters among Brazilian older adults using network analysis: Findings and perspectives. PLoS ONE. 2022;17(7):e0271639.

65. Puri P, Singh SK, Pati S. Identifying non-communicable disease multimorbidity patterns and associated factors: a latent class analysis approach. BMJ Open. 2022;12(7):e053981.

66. Whitson HE, Johnson KS, Sloane R, Cigolle CT, Pieper CF, Landerman L, et al. Identifying Patterns of Multimorbidity in Older Americans: Application of Latent Class Analysis. J Am Geriatr Soc. 2016;64(8):1668-73.

67. Cigolle C, Liang J, Ha J, Min L, Gure T, Lee P, et al. Investigating older adult multimorbidity: A latent class factor model of chronic diseases and geriatric conditions. J Gen Intern Med. 2012;2):S233-S4.

68. Jackson CA, Dobson AJ, Tooth LR, Mishra GD. Lifestyle and Socioeconomic Determinants of Multimorbidity Patterns among Mid-Aged Women: A Longitudinal Study. PLoS ONE. 2016;11(6):e0156804.

69. Eyowas FA, Schneider M, Alemu S, Pati S, Getahun FA. Magnitude, pattern and correlates of multimorbidity among patients attending chronic outpatient medical care in Bahir Dar, northwest Ethiopia: The application of latent class analysis model. PLoS ONE. 2022;17(4):e0267208.

70. Lind KE, Raban MZ, Brett L, Jorgensen ML, Georgiou A, Westbrook JI. Measuring the prevalence of 60 health conditions in older Australians in residential aged care with electronic health records: a retrospective dynamic cohort study. Popul Health Metr. 2020;18(1):25.

71. Islam MM, Valderas JM, Yen L, Dawda P, Jowsey T, McRae IS. Multimorbidity and comorbidity of chronic diseases among the senior Australians: prevalence and patterns. PLoS ONE. 2014;9(1):e83783.

72. Marventano S, Ayala A, Gonzalez N, Rodriguez-Blazquez C, Garcia-Gutierrez S, Forjaz MJ. Multimorbidity and functional status in community-dwelling older adults. Eur. 2014;25(7):610-6.

73. Machon M, Mateo-Abad M, Clerencia-Sierra M, Guell C, Poblador-Pou B, Vrotsou K, et al. Multimorbidity and functional status in older people: a cluster analysis. Eur Geriatr Med. 2020;11(2):321-32.

74. Gu J, Chao J, Chen W, Xu H, Zhang R, He T, et al. Multimorbidity and health-related quality of life among the community-dwelling elderly: A longitudinal study. Arch Gerontol Geriatr. 2018;74:133-40.

75. Deruaz-Luyet A, N'Goran AA, Senn N, Bodenmann P, Pasquier J, Widmer D, et al. Multimorbidity and patterns of chronic conditions in a primary care population in Switzerland: a cross-sectional study. BMJ Open. 2017;7(6):e013664.

76. Wang XX, Lin WQ, Chen XJ, Lin YY, Huang LL, Zhang SC, et al. Multimorbidity associated with functional independence among community-dwelling older people: a cross-sectional study in Southern China. Health Qual Life Outcomes. 2017;15(1):73.

77. Wartelle A, Mourad-Chehade F, Yalaoui F, Questiaux H, Monneret T, Soliveau G, et al. Multimorbidity clustering of the emergency department patient flow: Impact analysis of new unscheduled care clinics. PLoS ONE. 2022;17(1):e0262914.

78. Kuwornu JP, Lix LM, Shooshtari S. Multimorbidity disease clusters in Aboriginal and non-Aboriginal Caucasian populations in Canada. Chronic Dis Inj Can. 2014;34(4):218-25.

79. Nunes BP, Camargo-Figuera FA, Guttier M, de Oliveira PD, Munhoz TN, Matijasevich A, et al. Multimorbidity in adults from a southern Brazilian city: occurrence and patterns. Int J Public Health. 2016;61(9):1013-20.

80. Ruiz M, Bottle A, Long S, Aylin P. Multi-Morbidity in Hospitalised Older Patients: Who Are the Complex Elderly? PLoS ONE. 2015;10(12):e0145372.

81. Guo X, Zhao B, Chen T, Hao B, Yang T, Xu H. Multimorbidity in the elderly in China based on the China Health and Retirement Longitudinal Study. PLoS ONE. 2021;16(8):e0255908.

82. Yao SS, Xu HW, Han L, Wang K, Cao GY, Li N, et al. Multimorbidity measures differentially predicted mortality among older Chinese adults. J Clin Epidemiol. 2022;146:97-105.

83. Marengoni A, Tazzeo C, Calderon-Larranaga A, Roso-Llorach A, Onder G, Zucchelli A, et al. Multimorbidity Patterns and 6-Year Risk of Institutionalization in Older Persons: The Role of Social Formal and Informal Care. J Am Med Dir Assoc. 2021;22(10):2184-9.e1.

84. Zhang Q, Han X, Zhao X, Wang Y. Multimorbidity patterns and associated factors in older Chinese: results from the China health and retirement longitudinal study. BMC geriatr. 2022;22(1):470.

85. Zhang Q, Han X, Zhao X, Wang Y. Multimorbidity patterns and associated factors in older Chinese: results from the China health and retirement longitudinal study. BMC geriatr. 2022;22(1):470.

86. Rodrigues LP, Vissoci JRN, Franca DG, Caruzzo NM, Batista SRR, de Oliveira C, et al. Multimorbidity patterns and hospitalisation occurrence in adults and older adults aged 50 years or over. Sci. 2022;12(1):11643.

87. Liu C, Shu R, Liang H, Liang Y. Multimorbidity Patterns and the Disablement Process among Public Long-Term Care Insurance Claimants in the City of Yiwu (Zhejiang Province, China). Int J Environ Res Public Health. 2022;19(2):06.

88. Zacarias-Pons L, Vilalta-Franch J, Turro-Garriga O, Saez M, Garre-Olmo J. Multimorbidity patterns and their related characteristics in European older adults: A longitudinal perspective. Arch Gerontol Geriatr. 2021;95:104428.

89. Jackson CA, Jones M, Tooth L, Mishra GD, Byles J, Dobson A. Multimorbidity patterns are differentially associated with functional ability and decline in a longitudinal cohort of older women. Age Ageing. 2015;44(5):810-6.

90. Garin N, Olaya B, Perales J, Moneta MV, Miret M, Ayuso-Mateos JL, et al. Multimorbidity patterns in a national representative sample of the Spanish adult population. PLoS ONE. 2014;9(1):e84794.

91. Buja A, Claus M, Perin L, Rivera M, Corti MC, Avossa F, et al. Multimorbidity patterns in high-need, high-cost elderly patients. PLoS ONE. 2018;13(12):e0208875.

92. Clerencia-Sierra M, Calderon-Larranaga A, Martinez-Velilla N, Vergara-Mitxeltorena I, Aldaz-Herce P, Poblador-Plou B, et al. Multimorbidity Patterns in Hospitalized Older Patients: Associations among Chronic Diseases and Geriatric Syndromes. PLoS ONE. 2015;10(7):e0132909.

93. Aoki T, Yamamoto Y, Ikenoue T, Onishi Y, Fukuhara S. Multimorbidity patterns in relation to polypharmacy and dosage frequency: a nationwide, cross-sectional study in a Japanese population. Sci. 2018;8(1):3806.

94. Ioakeim-Skoufa I, Poblador-Plou B, Carmona-Pirez J, Diez-Manglano J, Navickas R, Gimeno-Feliu LA, et al. Multimorbidity Patterns in the General Population: Results from the EpiChron Cohort Study. Int J Environ Res Public Health. 2020;17(12):14.

95. Dong HJ, Wressle E, Marcusson J. Multimorbidity patterns of and use of health services by Swedish 85-year-olds: an exploratory study. BMC geriatr. 2013;13:120.

96. Zador Z, Landry A, Cusimano MD, Geifman N. Multimorbidity states associated with higher mortality rates in organ dysfunction and sepsis: a data-driven analysis in critical care. Crit Care. 2019;23(1):247.

97. Simoes D, Araujo FA, Severo M, Monjardino T, Cruz I, Carmona L, et al. Patterns and Consequences of Multimorbidity in the General Population: There is No Chronic Disease Management Without Rheumatic Disease Management. Arthritis Care Res (Hoboken). 2017;69(1):12-20.

98. John R, Kerby DS, Hennessy CH. Patterns and impact of comorbidity and multimorbidity among community-resident American Indian elders. Gerontologist. 2003;43(5):649-60.

99. Zheng DD, Christ SL, Lam BL, Feaster DJ, McCollister K, Lee DJ. Patterns of chronic conditions and their association with visual impairment. Investigative Ophthalmology and Visual Science Conference. 2019;60(9).

100. Formiga F, Ferrer A, Sanz H, Marengoni A, Alburquerque J, Pujol R, et al. Patterns of comorbidity and multimorbidity in the oldest old: the Octabaix study. Eur. 2013;24(1):40-4.

101. Teh RO, Menzies OH, Connolly MJ, Doughty RN, Wilkinson TJ, Pillai A, et al. Patterns of multi-morbidity and prediction of hospitalisation and all-cause mortality in advanced age. Age Ageing. 2018;47(2):261-8.

102. Piotrowicz K, Pac A, Skalska A, Mossakowska M, Chudek J, Zdrojewski T, et al. Patterns of multimorbidity in 4588 older adults. Implications for non-geriatrician specialist. Polish archives of internal medicine. 2021;22.

103. Juul-Larsen HG, Andersen O, Bodilsen AC, Jorgensen LM, Bandholm TQ, Petersen J. Patterns of multimorbidity in older medical patients (>= 65 years): And how they relate to mobility the first year after an acute admission. Eur Geriatr Med. 2018;9(Supplement 1):S279-S80.

104. Kirchberger I, Meisinger C, Heier M, Zimmermann A-K, Thorand B, Autenrieth CS, et al. Patterns of Multimorbidity in the Aged Population. Results from the KORA-Age Study. PLoS ONE. 2012;7(1).

105. Tan XW, Xie Y, Lew JK, Lee PSS, Lee ES. Patterns of patients with multiple chronic conditions in primary care: A cross-sectional study. PLoS ONE. 2020;15(8):e0238353.

106. Kshatri JS, Palo SK, Bhoi T, Barik SR, Pati S. Prevalence and Patterns of Multimorbidity Among Rural Elderly: Findings of the AHSETS Study. Front. 2020;8:582663.

107. Hunter ML, Knuiman MW, Musk BAW, Hui J, Murray K, Beilby JP, et al. Prevalence and patterns of multimorbidity in Australian baby boomers: the Busselton healthy ageing study. BMC Public Health. 2021;21(1):1539.

108. Lin WQ, Yuan LX, Sun MY, Wang C, Liang EM, Li YH, et al. Prevalence and patterns of multimorbidity in chronic diseases in Guangzhou, China: a data mining study in the residents' health records system among 31 708 community-dwelling elderly people. BMJ Open. 2022;12(5):e056135.

109. Jovic D, Vukovic D, Marinkovic J. Prevalence and Patterns of Multi-Morbidity in Serbian Adults: A Cross-Sectional Study. PLoS ONE. 2016;11(2):e0148646.

110. Craig LS, Hotchkiss DR, Theall KP, Cunningham-Myrie C, Hernandez JH, Gustat J. Prevalence and patterns of multimorbidity in the Jamaican population: A comparative analysis of latent variable models. PLoS ONE. 2020;15(7):e0236034.

111. Poblador-Plou B, van den Akker M, Vos R, Calderon-Larranaga A, Metsemakers J, Prados-Torres A. Similar multimorbidity patterns in primary care patients from two European regions: results of a factor analysis. PLoS ONE. 2014;9(6):e100375.

112. Ibarra-Castillo C, Guisado-Clavero M, Violan-Fors C, Pons-Vigues M, Lopez-Jimenez T, Roso-Llorach A, et al. Survival in relation to multimorbidity patterns in older adults in primary care in Barcelona, Spain (2010-2014): a longitudinal study based on electronic health records. J Epidemiol Community Health. 2018;72(3):185-92.

113. Tang LH, Thygesen LC, Willadsen TG, Jepsen R, la Cour K, Frolich A, et al. The association between clusters of chronic conditions and psychological well-being in younger and older people-A cross-sectional, population-based study from the Lolland-Falster Health Study, Denmark. J. 2020;10:2235042X20981185.

114. Hsu H-C. Trajectories of multimorbidity and impacts on successful aging. Exp Gerontol. 2015;66:32-8.

115. Ho HE, Yeh CJ, Wei JC, Chu WM, Lee MC. Trends of Multimorbidity Patterns over 16 Years in Older Taiwanese People and Their Relationship to Mortality. Int J Environ Res Public Health. 2022;19(6):11.

116. Grant RW, McCloskey J, Hatfield M, Uratsu C, Ralston JD, Bayliss E, et al. Use of Latent Class Analysis and k-Means Clustering to Identify Complex Patient Profiles. JAMA netw. 2020;3(12):e2029068.

117. Prenovost KM, Fihn SD, Maciejewski ML, Nelson K, Vijan S, Rosland A-M. Using item response theory with health system data to identify latent groups of patients with multiple health conditions. PLoS ONE. 2018;13(11).

118. Zhong Y, Qin G, Xi H, Cai D, Wang Y, Wang T, et al. Prevalence, patterns of multimorbidity and associations with health care utilization among middle-aged and older people in China. BMC Public Health. 2023;23(1):537.

119. Amirzada M, Buczak-Stec E, König HH, Hajek A. Multimorbidity patterns in the German general population aged 40 years and over. Arch Gerontol Geriatr. 2023;114:105067.

120. Roomaney RA, van Wyk B, Cois A, Pillay van-Wyk V. Multimorbidity patterns in South Africa: A latent class analysis. Front Public Health. 2022;10:1082587.

121. Chen H-L, Yu X-H, Yin Y-H, Shan E-F, Xing Y, Min M, et al. Multimorbidity patterns and the association with health status of the oldest-old in long-term care facilities in China: a two-step analysis. BMC geriatr. 2023;23(1):851.

122. Ioakeim-Skoufa I, Clerencia-Sierra M, Moreno-Juste A, Elías de Molins Peña C, Poblador-Plou B, Aza-Pascual-Salcedo M, et al. Multimorbidity Clusters in the Oldest Old: Results from the EpiChron Cohort. Int J Environ Res Public Health. 2022;19(16).

123. Zhong Y, Xi H, Guo X, Wang T, Wang Y, Wang J. Gender and Socioeconomic Differences in the Prevalence and Patterns of Multimorbidity among Middle-Aged and Older Adults in China. Int J Environ Res Public Health. 2022;19(24).

124. Zheng DD, McCollister KE, Christ SL, Lam BL, Feaster DJ, Lee DJ. Chronic condition patterns in the US population and their association with health related quality of life. Prev Med. 2020;136:106102.

125. Fagbamigbe AF, Agrawal U, Azcoaga-Lorenzo A, MacKerron B, Özyiğit EB, Alexander DC, et al. Clustering long-term health conditions among 67728 people with multimorbidity using electronic health records in Scotland. PLoS ONE. 2023;18(11):e0294666.

|  |
| --- |
