# Supplementary material for: Identifying clusters of multimorbid disease and differences by age, sex, and socioeconomic status: A systematic review
Source: PLoS One. 2025 Aug 22;20(8):e0329794. doi: 10.1371/journal.pone.0329794 (PMC12373218; doi:10.1371/journal.pone.0329794)
Supplement: S2 Table — (DOCX) [file pone.0329794.s006.docx]

**Supplementary Table 2: Data Extraction – Clusters of disease.**

|  | | | |
| --- | --- | --- | --- |
| **Paper #** | **Title** | **Stratification Variable (s)** | **Top 5 Clusters Identified** |
| 65 | Changes in Multimorbidity and Polypharmacy Patterns in Young and Adult Population over a 4-Year Period: A 2011-2015 Comparison Using Real-World Data | *Age*  *<65* | i) Mental Health iv) Respiratory iii) Allergic iv) Mechanical Pain v) Cardiometabolic |
| 149 | Prevalence and patterns of multimorbidity in the Jamaican population: A comparative analysis of latent variable models | *age*  15-74 | LCA: i) Relatively Healthy ii) Metabolic iii) Vascular-Inflammatory iv) Respiratory   EFA: i) Vascular - Hypertension, Obesity, Hypertension, Stroke ii) Respiratory iii) Cardiovascular, Mental and Articular |
| 9 | Patterns of multimorbidity and demographic profile of latent classes in a Danish population-A register-based stud | age  16-44 45-64 65+ | 16-44 years i) No or few diseases  ii) Bone and Joint diseases  iii) Mental illness, Epilepsy  iv) Asthma, Allergy  v) Diabetes, Heart diseases   45-64 years i) No or few diseases  ii) Diabetes, Cholesterol  iii) Bone, Joint diseases  iv) Mental illness, Epilepsy  v) Heart diseases   65+ years i) No or few diseases  ii) Diabetes, High cholesterol  iii) Heart disease  iv) Back disease, Asthma, Allergy  v) Many diseases |
| 79 | Emerging multimorbidity patterns and their links with selected health outcomes in a working-age population group | *Age*  16-64 | i) Low Co-Morbidity ii) Hypertension, Diabetes, Arthritis |
| 12 | Characteristics, service use and mortality of clusters of multimorbid patients in England: A population-based study | age  18-44 45-64 65-84 85+ | 18-44 years i) Depression, Anxiety, Pain ii) Pain, Hearing loss, Hypertension  iii) Asthma, IBS, Depression  45-64 years i) Hypertension, Diabetes, Pain ii) IBS, Hearing loss, Pain iii) Depression, Pain, Anxiety   65-84 years i) Hypertension, Diabetes, Pain ii) Hearing loss, Prostate disorder, IBS iii) Depression, Pain, Anxiety   85+ years i) Hypertension, Hearing loss, Diabetes ii) Pain, Depression, Constipation iii) CHD, Atrial fibrillation, Heart failure |
| 66 | Characteristics, service use and mortality of clusters of multimorbid patients in England: a population-based study | age  18-44 45-64 65-84 85+ | 18-44 years: i) Depression, Anxiety, Pain ii) Pain, Hearing Loss, Hypertension iii) Asthma, IBS, Depression iv) IBS, Depression, Hearing Loss v) Substance Misuse, Alcohol, Depression  45-64 years: i) Hypertension, Diabetes, Pain ii) IBS, Hearing Loss, Pain iii) Depression, Pain, Anxiety iv) Asthma, Pain, COPD v) Alcohol, Substance Misuse, Pain   65-84 years: i) Hypertension, Diabetes, Pain ii) Hearing Loss, Prostate Disorder, IBS iii) Depression, Pain, Anxiety iv) Coronary Heart Disease, Diabetes, Atrial Fibrillation v) COPD, Asthma, Pain  85+ years:  i) Hypertension, Hearing Loss, Diabetes ii) Pain, Depression, Constipation iii) Coronary Heart Disease, AF, Heart Failure iv) Asthma, COPD, Pain |
| 124 | Multimorbidity patterns in relation to polypharmacy and dosage frequency: a nationwide, cross-sectional study in a Japanese population | *age*  18-84 | i) Cardiovascular, Renal Metabolic ii) Neuropsychiatric  iii) Skeletal, Articular, Digestive iv) Respiratory, Dermal v) Malignant, Digestive, Urologic |
| 62 | Analysis of multiple chronic disease characteristics in South Koreans by age groups using association rules analysis | age  19-44 45-64 65+ | 19--44 years: i) Diabetes, Hypertension ii) Diabetes, Dyslipidemia   45-64 years: i) Stroke, Hypertension ii) Diabetes, Hypertension iii) Heart Disease, Hypertension iv) Dyslipidemia, Hypertension v) Arthritis, Hypertension  >65 years:  i) Stroke, Hypertension ii) Dyslipidemia, Hypertension iii) Diabetes, Hypertension iv) Heart Disease, Hypertension v) Arthritis, Hypertension |
| 148 | Prevalence and Patterns of Multi-Morbidity in Serbian Adults: A Cross-Sectional Study | age and sex   20-44 45-64 65+ | 20-44 years: FEMALE: i) Non-Communicable - MI, Malignancy, Stroke  ii) Cardiometabolic  iii) Respiratory - Asthma, COPD, Bronchitis, Emphysema, and CKD  MALE: i) Non-communicable - Stroke, MI, Malignancy, Chronic Bronchitis/COPD  ii) Cardiometabolic - Hypertension, Hyperlipidemia, Obesity   45 - 64 years:  FEMALE: i) Cardiometabolic ii) Respiratory and Depression iii) Cardiovascular and Depression   MALE: i) Respiratory - Asthma, COPD, Emphysema ii) Cardiometabolic - Hypertension, Obesity, Diabetes, Hyperlipidemia  iii) Aggregate - Joint disease, Depression, CKD, Stroke, Malignancy  iv) Cardiovascular - MI, Coronary Heart Disease, Hyperlipidemia   65+ years:  FEMALE: i) Respiratory ii) Cardiometabolic  iii) MI, Coronary Heart Disease, Stroke, CKD iv) Mechanical, Mental, Metabolic  MALE:  i) Respiratory - Chronic Bronchitis, COPD, Emphysema, Asthma ii) Cardiometabolic and Coronary Heart Disease  iii) Cardiovascular - Hyperlipidemia, MI, Coronary Heart Disease, Malignancy, Stroke iv) Mechanical, Mental and Metabolic |
| 115 | Multimorbidity patterns and association with mortality in 0.5 million Chinese adults | *Age*  30-79 | i) Cardiometabolic ii) GI and Hepatorenal  iii) Respiratory iv) Mental and Arthritis |
| 21 | Patterns of multimorbidity in working Australians | *Age*  30+ | i) Arthritis, Osteoporosis, Chronic pain, Bladder problems, Irritable bowel ii) Asthma, COPD, Allergies iii) Back/neck pain, Migraine, Other chronic pain, Arthritis iv) Hypertension, High cholesterol, Obesity, Diabetes, Fatigue v) Cardiovascular disease, Diabetes, Fatigue, Hypertension, Hypercholesterolemia   ** does not show prevalence ** |
| 92 | Magnitude, pattern and correlates of multimorbidity among patients attending chronic outpatient medical care in Bahir Dar, northwest Ethiopia: The application of latent class analysis model | *Age*  40+ | i) Cardiovascular - Heart Disease, Hypertension ii) Metabolic - Diabetes and Hypertension iii) Cardio-Mental - Heart Disease, Depression iv) Respiratory - Asthma and Stroke |
| 11 | Multimorbidity patterns with K-means nonhierarchical cluster analysis | *Age* 45-64 | i) Metabolic disorders, Hypertensive diseases, Mental/behavioural disorders (psychoactive substance use), Dorsopathies, Soft tissue disorder ii) GI conditions, Musculoskeletal conditions iii) connective tissue diseases iv) Cardiometabolic pattern - obesity, hypertension and diabetes v) Infections and Injuries |
| 28 | Comparing Multimorbidity Patterns Among Discharged Middle-Aged and Older Inpatients Between Hong Kong and Zurich: A Hierarchical Agglomerative Clustering Analysis of Routine Hospital Records | *Age*  45+ | *Stratification by Country*   Zurich: i) Diabetes, Parkinson’s, Heart Failure . ii) Cancer, Peripheral Vascular Disease, Heart Failure  iii) Atrial Fibrillation, Chronic Kidney Disease, Chronic Pain iv) Lymphoma, Metastatic Cancer, Cirrhosis  v) Hyperthyroidism, Epilepsy, Hypertension   Hong Kong:  i) Cancer, Cirrhosis, Diabetes  ii) Chronic pain, Heart Failure, Hypertension iii) Atrial Fibrillation, Chronic Kidney Disease, MI iv) Heart Failure, Hypertension, Chronic Pain  v) Chronic Pulmonary Disease, IBS, Peripheral Vascular Disease |
| 50 | Identifying co-occurrence and clustering of chronic diseases using latent class analysis: Cross-sectional findings from SAGE South Africa Wave 2 | *Age*  45+ | i) Minimal MM risk  ii) Concordant - Hypertension, Diabetes iii) Discordant - Angina, Asthma, Chronic lung disease, Arthritis, Depression |
| 63 | Association of multimorbidity patterns with incident disability and recovery of independence among middle-aged and older adults | *Age*  45+ | i) Low Morbidity ii) Pulmonary, Digestive, Rheumatic iii) Cardiovascular, Endocrine, Neuro iv) High Morbidity |
| 91 | Lifestyle and Socioeconomic Determinants of Multimorbidity Patterns among Mid-Aged Women: A Longitudinal Study | *Age*  45+ | i) Psychosomatic - Anxiety, Depression, Somatic symptoms ii) Musculoskeletal - Arthritis, Joint pain, Back pain iii) Cardiometabolic - CVS disease, Diabetes, Impaired Glucose Tolerance iv) Cancer v) Respiratory - Asthma, Bronchitis, Emphysema, Breathing Difficulties |
| 88 | Identifying non-communicable disease multimorbidity patterns and associated factors: a latent class analysis approach | *Age*  45+ | i) Relatively Healthy  ii) Hypertension iii) GI Disorders, Hypertension, Musculoskeletal Disorders  iv) Metabolic Disorders - Hypertension, Diabetes, Hypertension v) Complex Cardiometabolic Disorders |
| 23 | Description of multimorbidity clusters of admitted patients in medical departments of a general hospital | *Age*  50-72 | i) Hypertension/hypertensive heart disease, Type 2 diabetes mellitus, Ischaemic cardiomyopathy, Dyslipidemia ii) Atrial fibrillation/flutter, Cardiac failure, Chronic kidney failure, Heart valve disease iii) Malnutrition, Parkinson disease, Dementia, Mental health conditions  iv) Large intestine, Prostate, Breast neoplasms, Lymphoma and Myeloma v) Alcoholic liver disease, Alcoholic dependency syndrome, Lung, Digestive tract malignant neoplasms |
| 24 | Multimorbidity patterns in low-middle and high income regions: a multiregion latent class analysis using ATHLOS harmonised cohorts | *Age*  50+ | i) Healthy  ii) Cardio-metabolic  iii) Respiratory-mental-articular |
| 27 | Global Multimorbidity Patterns: A Cross-Sectional, Population-Based, Multi-Country Study (WC) | *Age*  50+ | i) Cardio-respiratory (7 countries) - Angina, Asthma, COPD  ii) Metabolic (8 countries) - Diabetes, Obesity, Hypertension iii) Mental-articular (3 countries) - Arthritis, Depression iv) Respiratory (2 countries) - Asthma, COPD |
| 29 | Prevalence and Patterns of Multimorbidity in a Nationally Representative Sample of Older Chinese: Results From the China Health and Retirement Longitudinal Study | *Age*  50+ | HCA:  i) Vascular-metabolic - Hypertension, Dyslipidemia, Diabetes, Stroke ii) Stomach-arthritis - Stomach, Digestive disease, Arthritis iii) Cognitive-emotional - Memory-related disease, Emotional, Nervous or psychiatric problem iv) Hepatorenal - Liver disease, kidney disease |
| 32 | Physical multimorbidity, depressive symptoms, and social participation in adults over 50 years of age: findings from the English Longitudinal Study of Ageing | *Age*  50+ | i) Healthy  ii) Hypertension and diabetes  iii) Respiratory  iv) Complex and multisystem |
| 36 | Use of latent class analysis to identify multimorbidity patterns and associated factors in Korean adults aged 50 years and older | *Age*  50+ | i) Relatively healthy  ii) Cardiometabolic conditions  iii) Arthritis, Asthma, Allergic rhinitis, Depression, Thyroid |
| 41 | Latent class analysis of multimorbidity patterns and associated outcomes in Spanish older adults: a prospective cohort study | *Age*  50+ | i) Minimum disease  ii) Metabolic, Stroke, Obesity, Diabetes, Hypertension  iii) Cardiorespiratory, Mental, Arthritis |
| 78 | Effect of multimorbidity patterns on the decline in health-related quality of life: a nationwide prospective cohort study in Japan | *Age*  50+ | i) Cardiovascular, Renal, Metabolic ii) Neuro, Psychiatric iii) Respiratory and Dermal iv) Skeletal, Articular, and Digestive v) Malignant, Digestive, Urologic |
| 81 | Multimorbidity Patterns and Their Association with Social Determinants, Mental and Physical Health during the COVID-19 Pandemic | *Age*  50+ | i) Relatively Healthy ii) Cardiometabolic iii) Musculoskeletal iv) Musculoskeletal and Mental v) Complex Multimorbidity |
| 90 | Investigating older adult multimorbidity: A latent class factor model of chronic diseases and geriatric conditions | *Age*  50+ | i) Healthy  ii) Metabolic, Stroke iiii) Cardiorespiratory, Mental, Arthritis |
| 97 | Multimorbidity and comorbidity of chronic diseases among the senior Australians: prevalence and patterns | *Age*  50+ | HCA i) Asthma, Bronchitis, Depression, Arthritis, Osteoporosis ii) Heart Disease, Stroke, Hypertension, Diabetes iii) Cancer   LCA i) Asthma, Bronchitis, Arthritis, Osteoporosis, Depression  ii) Hypertension, Diabetes iii) Cancer, Heart Disease, Stroke |
| 116 | Multimorbidity patterns and hospitalisation occurrence in adults and older adults aged 50 years or over | *Age*  50+ | i) Cardiometabolic Diseases, Cancer, and Others  ii) Respiratory - Asthma and COPD iii) Diabetes, Glaucoma, Retinopathy and Macular Degeneration iv) Neurodegenerative - Parkinson’s and Alzheimer v) Musculoskeletal - Spinal problems, Arthritis, Rheumatism, Osteoporosis |
| 121 | Multimorbidity patterns in a national representative sample of the Spanish adult population | *Age*  50+ | i) Cardiorespiratory - Angina, Asthma, Chronic Lung Disease ii) Arthritis, Anxiety, Depression  iii) Hypertension, Angina, Stroke, Diabetes, Cataracts |
| 157 | Trends of Multimorbidity Patterns over 16 Years in Older Taiwanese People and Their Relationship to Mortality | *Age*  50+ | i) Relatively Healthy  ii) Cardiometabolic iii) Arthritis-Cataract iv) 'Multimorbidity' Group |
| 30 | Racial and Ethnic Differences in Multimorbidity Changes over Time | *Age*  51+ | 1998:  i) Minimal disease  ii) Cardiovascular-musculoskeletal  iii) Cardiovascular-musculoskeletal-mental   2014: i) Multisystem multimorbidity ii) Cardiovascular-musculoskeletal  iii) Cardiovascular-musculoskeletal-metabolic |
| 25 | Comparisons of disease cluster patterns, prevalence and health factors in the USA, Canada, England and Ireland | *Age*  52+ | i) High probability of disease ii) Metabolic, Cardiovascular, Arthritis, Cancer  iii) Osteoporosis, Arthritis iv) Metabolic, Arthritis, Psychological  v) Low probability of disease  ** Prevalence different depending on country ** |
| 135 | Patterns of multimorbidity in 4588 older adults. Implications for non-geriatrician specialist | age  55-59 65-79 80+ | 55-59 years:  i) Cardiovascular ii) Cognitive Impairment and Parkinson's, epilepsy iii) CKD, Hypertension, Eye Disorders, Cognitive Impairment iv) Osteoporosis, Psychiatric, Thyroid, Metabolic Diseases  65-79 years i) Cardiovascular ii) Cognitive Impairment, Parkinsons, Epilepsy, Psychiatric, Stroke iii) Cardiometabolic iv) Renal v) Osteoporosis, thyroid, Eye Disorders  80+ years i) Cardiovascular ii) Metabolic, Parkinsons, Epilepsy iii) CKD, Anemia, Cancer iv) Osteoporosis, Eye Disorders v) Cognitive Impairment |
| 75 | Disease Combinations Associated with Physical Activity Identified: The SMILE Cohort Study | *Age*  55+ | i) Bowel Disease, Kidney Disease, Malignancy/Cancer  ii) Liver Disease, Cirrhosis, Back problems, RA iii) MI, Diabetes, Chronic Bronchitis, Emphysema, Asthma |
| 146 | Prevalence and patterns of multimorbidity in Australian baby boomers: the Busselton healthy ageing study | *Age*  59+ | i) Healthy  ii) Non-Cardiometabolic iii) Respiratory and Atopy iv) Cardiometabolic |
| 22 | Multimorbidity Patterns in Older Adults: An Approach to the Complex Interrelationships Among Chronic Diseases | *Age*  60+ | i) Cardiac, Respiratory, Hypertension  ii) Vascular, Upper gastrointestinal, Musculoskeletal  iii) Endocrine, Renal  iv) Psychological, Neurological  v) Neoplasia |
| 37 | Multimorbidity patterns in old adults and their associated multi-layered factors: a cross-sectional study | *Age*  60+ | i) Digestive diseases, Arthritis, Hearing loss, Osteoporosis ii) Cardiovascular diseases, Hypertension, Coronary heart disease  iii) Metabolic diseases - Diabetes, Hypertension |
| 39 | The Differential Impact of Multimorbidity Patterns and Subsequent Accumulation on Longitudinal Trajectories of Physical Function Decline in a Population-based Cohort of Older People | *Age*  60+ | i) Hypertension, Arthritis  ii) Lung and Asthma  iii) Hepatorenal/ Multisystem - Cancer, Liver disease, Kidney disease iv) Cardiometabolic  v) Stomach, Arthritis |
| 47 | Multimorbidity in the community-dwelling elderly in urban China | *Age*  60+ | i) Cardiovascular diseases, dyslipidemia, hypertension, diabetes, Kidney disease  ii) Degenerative diseases, hearing disorder, cataract, joint disease, Cancer iii) Liver disease, Lung disease, Gastrointestinal disease |
| 51 | Prevalence and patterns of chronic disease pairs and multimorbidity among older Chinese adults living in a rural area | *Age*  60+ | i) Cerebrovascular and Metabolic - Stroke, hypertension, diabetes, dyslipidemia, and obesity ii) Cardiopulmonary disorders, Depression, Degenerative disorders |
| 53 | Inequalities in multimorbidity among elderly: a population-based study in a city in Southern Brazil | *Age*  60+ | i) Musculoskeletal, Mental, Functional disorders ii) Cardiometabolic - Hypertension, Diabetes, Cardiovascular, Stroke iii) Respiratory factors - Asthma, Bronchitis, Emphysema, Rhinitis |
| 87 | Identifying multimorbidity clusters among Brazilian older adults using network analysis: Findings and perspectives | *Age*  60+ | i) Cardiometabolic - Hypertension, Diabetes, Heart Disease, Stroke, Kidney Disease ii) Respiratory and Cancer - COPD, Asthma, Cancer iii) Musculoskeletal - Low Back Pain, Arthritis, Rheumatism, Work Related MSK diseases iv) Mixed Mental Illness + Other - Depression, Mental Illness, Other Chronic Diseases |
| 100 | Multimorbidity and health-related quality of life among the community-dwelling elderly: A longitudinal study | *Age*  60+ | i) Degenerative Disorders ii) Digestive and Respiratory iii) Cardiometabolic |
| 102 | Multimorbidity associated with functional independence among community-dwelling older people: a cross-sectional study in Southern China | *Age*  60+ | i) Cardiometabolic  ii) Bones and Pain   iii) Gastroenteritis, CKD, PVD  iv) Neuropsychiatric and Stroke - Dementia  v) Lung and Cancer |
| 111 | Multimorbidity in the elderly in China based on the China Health and Retirement Longitudinal Study | *Age*  60+ | i) Asthma, Chronic Lung Disease ii) Asthma, Arthritis, Rheumatism, Chronic Lung Disease iii) Dyslipidemia, Hypertension, Arthritis and Rheumatism, MI |
| 113 | Multimorbidity Patterns and 6-Year Risk of Institutionalization in Older Persons: The Role of Social Formal and Informal Care | *Age*  60+ | i) Unspecific ii) MSK, Respiratory, and GI iii) Sensory Impairments and Cancer iv) Metabolic and Sleep Disorders v) CVS, Anemia, and Dementia |
| 114 | Multimorbidity patterns and associated factors in older Chinese: results from the China health and retirement longitudinal study | *Age*  60+ | i) Relatively Healthy ii) Vascular  iii) Respiratory iv) Stomach-Arthritis  v) Multisystem Morbidity |
| 118 | Multimorbidity Patterns and the Disablement Process among Public Long-Term Care Insurance Claimants in the City of Yiwu (Zhejiang Province, China) | *Age*  60+ | i) Other Diseases ii) Lower Limb Fractures  iii) Coronary Atherosclerotic Heart Disease |
| 130 | Patterns and impact of comorbidity and multimorbidity among community-resident American Indian elders | *Age*  60+ | i) Stroke, Heart Disease, Diabetes  ii) Vision Problems, Hearing Problems, Problems with Teeth and Gums  iii) Arthritis  iv) Depression |
| 145 | Prevalence and Patterns of Multimorbidity Among Rural Elderly: Findings of the AHSETS Study | *Age*  60+ | i) Arthritis ii) Hypertension and Peptic Acid Disease iii) All other conditions |
| 156 | Trajectories of multimorbidity and impacts on successful aging | *Age*  60+ | i) Low Risk ii) GI and CNS conditions  iii) Cardiovascular iv) Multiple Risks |
| 14 | Multimorbidity Patterns in Elderly Primary Health Care Patients in a South Mediterranean European Region: A Cluster Analysis | *Age* 64+ | i) Hypertensive diseases, Metabolic disorders  ic) Atrial fibrillation, Heart disease ii) Dorsopathies, Soft tissue disorders, Joint  iii) Heart disease, Mood disorders, Thyroid, Intestines, Lens  iv) Oral cavity, Salivary glands, Ear, URTI, Urinary diseases, |
| 5 | Soft clustering using real-world data for the identification of multimorbidity patterns in an elderly population: Cross-sectional study in a Mediterranean population | *Age* 65+ | i) Non-specified  ii) Genitourinary, Mental, Musculoskeletal  iii) Nervous, Musculoskeletal, Circulatory  iv) Mental, Digestive, Blood  v) Mental, Nervous, Digestive |
| 19 | Patterns of multimorbidity and differences in healthcare utilization and complexity among acutely hospitalized medical patients (>=65 years) - a latent class approach | *Age*  65+ | i) Minimal chronic conditions  ii) Cardiovascular disorders  iii) Neurological, Vascular  iv) Degenerative, Pulmonary disorders, Osteoporosis, COPD v) Metabolic and cardiovascular disorders, CVS, Diabetes |
| 38 | Multimorbidity Patterns, Frailty, and Survival in Community-Dwelling Older Adults | *Age*  65+ | i) Cardiovascular disease, Diabetes, Hypertension ii) Osteoarticular disease, Arthritis, Osteoporosis, Cancer  iii) Minimal disease  iv) High multisystem morbidity  v) Neuropsychiatric disease, Stroke, Psychiatric disorders, Dementia |
| 45 | Comparison of Disease Clusters in Two Elderly Populations Hospitalized in 2008 and 2010 | *Age*  65+ | FIRST WAVE  i) Liver cirrhosis, Malignancy  ii) COPD, Prostate hypertrophy  iii) Diabetes, CHD  iv) Dementia, Arthritis  v) Thyroid dysfunction, Anxiety    SECOND WAVE  i) Liver cirrhosis, Malignancy  ii) Diabetes, CHD, Dyslipidemia  iii) Hypertension, Thyroid dysfunction, AF  iv) CVD, Dementia, Arthritis  v) Anemia, Gastric diseases, Gut diseases, Anxiety   ** does not show prevalence ** |
| 56 | Multimorbidity patterns of chronic conditions and geriatric syndromes in older patients from the MoPIM multicentre cohort study | *Age*  65+ | i) Cardiorespiratory - COPD, Heart failure, Cardiac arrythmia ii) Minor chronic disease: Hypertension, Dyslipidemia, Anemia, Gout, Non ischemic heart disease iii) Psychogeriatric - Pressure ulcers, Immobility, Malnutrition, Cognitive impairment iv) Osteoarticular, Osteoporosis, Fractures, Inflammatory disease, Neuropathy |
| 58 | Differences in function and recovery profiles between patterns of multimorbidity among older medical patients the first year after an acute admission-An exploratory latent class analysis | *Age*  65+ | i) Minimal chronic disease  ii) Dementia, Disability, Brain infarction, Hypertension, CVS disease iii) Diabetes, Hypertension, COPD, Asthma, CVS disease iv) Degenerative, Lifestyle, Mental disorders, COPD/Asthma, Osteoporosis |
| 60 | Age, sex, residence, and region-specific differences in prevalence and patterns of multimorbidity among older Chinese: evidence from Chinese Longitudinal Healthy Longevity Survey | *Age*  65+ | i) Hypertension, Rheumatoid Arthritis, Vision Impairment ii) Hypertension, Vision Impairment, Rheumatoid Arthritis iii) Dyslipidemia, Cardiovascular Diseases iv) Hypertension, Respiratory Diseases, Cardiovascular Diseases v) Diabetes, Hypertension, Cardiovascular Diseases |
| 67 | Chronic condition clusters and associated disability over time | *Age*  65+ | i) Minimal Disease ii) Cognitive/Affective iii) Multiple Morbidity iv) Osteoporosis v) Vascular |
| 69 | Chronic disease and falls in community-dwelling Canadians over 65 years old: a population-based study exploring associations with number and pattern of chronic conditions | *Age*  65+ | i) Low Chronic Disease ii) Hypertension and Arthritis iii) Visual Impairment  iv) Hypertension v) COPD |
| 89 | Identifying Patterns of Multimorbidity in Older Americans: Application of Latent Class Analysis | *Age*  65+ | i) Minimal Disease  ii) Non-Vascular, - Cancer, Osteoporosis, Arthritis  iii) Vascular - Hypertension, Diabetes, Stroke iv) Cardiovascular, Stroke, Cancer, Arrythmia v) Major Neurologic Disease - Alzheimer's, Parkinsons, Psychiatric |
| 93 | Measuring the prevalence of 60 health conditions in older Australians in residential aged care with electronic health records: a retrospective dynamic cohort study | *Age*  65+ | i) Heart Disease, Cerebrovascular Disease, Arrythmias  ii) Constipation, Arthritis, CORD and Depression  iii) Hypertension, Heart Disease, Dyslipidemia and Diabetes iv) Dementia and Cognitive impairment, Urinary Incontinence, Osteoporosis  v) Arrythmias, Heart Failure, Breast Cancer |
| 98 | Multimorbidity and functional status in community-dwelling older adults | *Age*  65+ | i) Cardiometabolic  ii) Sensory and Bone iii) Cancer, Lung and GI  iv) Neuropsychiatric |
| 110 | Multi-Morbidity in Hospitalised Older Patients: Who Are the Complex Elderly? | *Age*  65+ | i) Cancer and Metastases ii) COPD, Lung Disease, Rheumatism and Osteoporosis iii) Heart Failure, Cerebrovascular, Diabetes, Hypertension, MI |
| 112 | Multimorbidity measures differentially predicted mortality among older Chinese adults | *Age*  65+ | i) Cardiometabolic ii) Mental-Sensory iii) Inflammatory-Digestive  iv) Other |
| 122 | Multimorbidity patterns in high-need, high-cost elderly patients | *Age*  65+ | i) Cardiac - Congestive Heart Failure, Fibrillation ii) Cardiovascular - Ischemic Heart Disease, Diabetes, Hypertension, Hyperlipidemia  iii) Neurological and Mental - Alzheimer's, Cerebrovascular, Depression iv) Cancer  v) Respiratory - Asthma and COPD |
| 123 | Multimorbidity Patterns in Hospitalized Older Patients: Associations among Chronic Diseases and Geriatric Syndromes | *Age*  65+ | I) Cardiovascular ii) Induced Dependency iii) Falls iv) Osteoarticular |
| 138 | Patterns of Multimorbidity in the Aged Population. Results from the KORA-Age Study | *Age*  65+ | i) Cardiometabolic ii) Joint, Liver, Lung, Eye iii) Mental and Neurological iv) GI diseases, Cancer |
| 147 | Prevalence and patterns of multimorbidity in chronic diseases in Guangzhou, China: a data mining study in the residents' health records system among 31 708 community-dwelling elderly people | *Age*  65+ | i) Hypertension and Diabetes ii) Hypertension and Coronary Heart Disease iii) Diabetes and Coronary Heart Disease iv) Diabetes, Coronary Heart Disease and Hypertension |
| 153 | Survival in relation to multimorbidity patterns in older adults in primary care in Barcelona, Spain (2010-2014): a longitudinal study based on electronic health records | *Age*  65+ | i) Musculoskeletal  ii) Endocrine and Metabolic iii) Neurological iv) Cardiovascular v) Digestive, Respiratory |
| 15 | Multimorbidity patterns in the elderly: a prospective cohort study with cluster analysis | *Age* 65+ | i) Nonspecific  ii) Musculoskeletal iii) Endocrine-metabolic iv) Digestive/Digestive-respiratory v) Neuropsychiatric |
| 73 | Deconstructing Complex Multimorbidity in the Very Old: Findings from the Newcastle 85+ Study | *Age*  75+ | i) Hypertension, Renal, Cerebrovascular . ii) Urinary Incontinence, Hearing impairment, Visual impairment  iii) Hearing impairment, Ischemic Heart Disease, Osteoporosis  iv) Osteoarthritis, Urinary Incontinence, Falls  v) Hearing Impairment, Visual Impairment, COPD |
| 72 | Comorbidity patterns in patients with chronic diseases in general practice | age  80+ | 80 years+ i) Congestive Heart Disease, Cardiac Arrythmia, Dementia, Chronic Ulcer |
| 133 | Patterns of multi-morbidity and prediction of hospitalisation and all-cause mortality in advanced age | *Age*  80+ *race* | *stratified between Māori and non maori*  Māori ii) CVD, Respiratory, Mental Health ii) Diabetes iii) "Well" iv) Chronic Heart Failure, AF v) Arthritis  Non-Māori  i) Respiratory and Diabetes ii) Stroke iii) "Well" iv) Depression and Arthritis v) Chronic Heart Failure, AF |
| 132 | Patterns of comorbidity and multimorbidity in the oldest old: the Octabaix study | *Age*  85+ | i) AF, Heart Failure, Visual Impairment, CKD, Stroke ii) COPD and Malignancy iii) Dementia, Parkinson's, Dyslipidemia, Peripheral Arterial Disease, Anemia iv) Auditory Impairment |
| 16 | Multimorbidity patterns and their relationship to mortality in the US older adult population | *Age   50+* | i) Healthy ii) Age-associated chronic conditions iii) Respiratory condition iv) Cognitively impaired  v) Complex cardiometabolic |
| 99 | Multimorbidity and functional status in older people: a cluster analysis | *Age* and frailty  70+ | *Stratifies by frailty*   Robust Group: i) Relatively Healthy  ii) Cataract and Eye Diseases, Colitis, Dorsopathies ...  iii) CKD, Diabetes, Heart Failure, Hypertension   Frail Group  i) Relatively Healthy  ii) COPD, Emphysema, Chronic Bronchitis, Dyslipidemia, ENT diseases iii) Bradycardia and Conduction Diseases, Cardiac Valve Diseases, Diabetes  iv) Anemia, Cataract and lens disease, CKD |
| 106 | Multimorbidity disease clusters in Aboriginal and non-Aboriginal Caucasian populations in Canada | age and race  18-54 55+ | First Nations 18-54 Years  i) High-Item Response ii) High Blood Pressure  iii) Back Problems iv) Ulcers   Non-First Nations 18-54 Years  i) Asthma ii) Hypertension  iii) Back Pain and Migraine   First Nations 55+ Years  i) High-Item Response ii) Arthritis, Rheumatism, and Hypertension iii) Arthritis, Rheumatism, Back Problems  iv) Back Problems, Cataracts, Emphysema, Ulcers  Non-First Nations 55+ Years  i) High-Item Response ii) Arthritis, Rheumatism, Back Problems, High Blood Pressure |
| 125 | Multimorbidity Patterns in the General Population: Results from the EpiChron Cohort Study | age and sex  15-29 30-44 45-49 60-74 75-89 90+ | 15-29 years: FEMALE: i) Allergic and Growth Development ii) Endocrine, Metabolic  iii) Mental Health  MALE: i) Allergic, Growth Development ii) Metabolic iii) Mental Health   30-44 years: FEMALE: i) Neuromuscular and Depressive  ii) Metabolic iii) Endocrine  MALE: i) Metabolic ii) Mental Health iii) Allergic iv) Sensory Processing   45-59 years:  FEMALE: i) Metabolic ii) Neuromuscular and Depressive iii) Peripheral, Vascular  MALE: i) Metabolic ii) Neuromuscular, Depresssive iii) Cardiovascular iv) Mental Health   60-74 years:  FEMALE: i) Metabolic ii) Neuromuscular Depressive iii) Peripheral Vascular iv) Cardiorespiratory   MALE: i) Metabolic  ii) Neuromuscular, Depressive iii) Cardiorespiratory  iv) Peripheral vascular   75-89 years:  FEMALE: i) Neuromuscular, Degenerative ii) Metabolic iii) Cardiorespiratory iv) Neurodegenerative, Vascular  MALE: i) Metabolic ii) Cardiorespiratory iii) Neuromuscular, Depressive iv) Neurodegenerative, Vascular  90+ years:  FEMALE: i) Neuromuscular, Degenerative ii) Metabolic iii) Cardiorespiratory iv) Neurodegenerative, Vascular  MALE: i) Neuromuscular, Degenerative ii) Cardiorespiratory iii) Neurodegenerative, Vascular |
| 152 | Similar multimorbidity patterns in primary care patients from two European regions: results of a factor analysis | age and sex  15-44 45-64 65+ | 15-44 years: FEMALE: i) Cardiometabolic  ii) Mechanical  MALE: i) Cardiometabolic - Hypertension, Hyperlipidemia, Obesity  ii) Psychiatric, Substance Abuse   45 - 64 years:  FEMALE: i) Cardiometabolic ii) Mechanical iii) Depressive  MALE: i) Cardiometabolic ii) Mechanical  65+ years:  FEMALE: i) Cardiometabolic ii) Psycho-geriatric iii) Mechanical iv) Depressive  MALE:  i) Cardiometabolic ii) Psycho-Geriatric iii) Mechanical |
| 65 | Changes in Multimorbidity and Polypharmacy Patterns in Young and Adult Population over a 4-Year Period: A 2011-2015 Comparison Using Real-World Data | age and sex  15-44 45-65 | 15-44 years: FEMALE: i) Mechanical Pain  ii) Respiratory  iii) Mental Health iv) Endocrinological   MALE: i) Mental Health ii) Mechanical Pain iii) Respiratory   45 - 65 years:  FEMALE: i) Mental Health ii) Respiratory iii) Cardiometabolic iv) Osteo-metabolic   MALE: i) Mental Health ii) Cardiometabolic iii) Respiratory |
| 12 | Characteristics, service use and mortality of clusters of multimorbid patients in England: A population-based study | age and sex  18-44 45-64 65-84 85+ | Female 18-44 years i) IBS, Depression, Hearing Loss ii) Depression, Anxiety, Pain iii) Asthma, IBS, Depression  Female 45-64 years i) Depression, Pain, Anxiety ii) IBS, Hearing loss, Pain iii) Asthma, Pain, COPD  Female 65-84 years i) Depression, Pain, Anxiety  ii) Hypertension, Diabetes, Pain iii) Pain, CHD, Depression  Female 85+ years i) Depression, Pain, Constipation ii) Hypertension, Hearing Loss, Diabetes iii) CHD, Atrial fibrillation, Heart failure |
| 33 | The patterns of Non-communicable disease Multimorbidity in Iran: A Multilevel Analysis | *age and* sex  20-70 | Male: i) Low risk of diseases ii) Musculoskeletal diseases - back/knee/osteoporosis  iii) Diabetes  iv) Asthma and Wheezing   Female: i) Low risk of diseases  ii) Pre-skeletal diseases  iii) Musculoskeletal diseases  iv) Asthma, Wheezing |
| 11 | Multimorbidity patterns with K-means non-hierarchical cluster analysis | *age and* sex  45-64 | FEMALE: i) Metabolic disorders, Hypertensive diseases, Mental/behavioural disorders (psychoactive substance use), Dorsopathies, Soft tissue disorder ii) Arthrosis, Disorders of bone density and structure, Nerve root plexus disorders, Spondylopathies, Deforming dorsopathies  iii) Benign neoplasms, Dermatitis, Eczema, Mycoses, Nutritional anemias  MALE: i) Metabolic disorders, Hypertensive diseases, Mental/behavioural disorders (psychoactive substance use), Dorsopathies, Soft tissue disorder ii) Mental/behavioural disorders, Chronic lower respiratory diseases, Mood disorders, Viral hepatitis, Liver disease  iii) Gi diseases, Hernia, Genital diseases, Veins/lymph, Benign neoplasms |
| 10 | Comparative analysis of methods for identifying multimorbidity patterns: A study of 'real-world' data | *age and* sex  45-64  clustering techniques: HCA/EFA | Men:  i) Metabolic disorders, Hypertensive diseases, Obesity and other hyperalimentation ii) Hypertensive diseases, Other forms of heart disease, Ischaemic heart diseases  iii) Mental and behavioural disorders due to psychoactive substance use , Diseases of liver, Viral hepatitis iv) Other dorsopathies, Arthrosis, Spondylopathies  v) Dermatitis and eczema, Mycoses, Visual disturbances   Combinations of diseases consistent across statistical methods (cluster and factor analysis)*  Women  i) Hypertensive diseases ii) Arthrosis, other dorsopathies, other soft-tissue disorders iii) Dermatitis and eczema, mycoses  iv) Spondylopathies, deforming dorsopathies v) Diseases of oesophagus, stomach and duodenum, hernia   Men i) Hypertensive diseases, obesity and other hyperalimentation, metabolic disorders (MC1; MF1), ischaemic heart diseases, cerebrovascular diseases iii) Influenza and pneumonia, other acute lower respiratory infections, other viral diseases, intestinal infectious diseases  iv) Disorders of conjunctiva, visual disturbances and blindness, other disorders of the skin and subcutaneous tissue  v) Spondylopathies, deforming dorsopathies |
| 120 | Multimorbidity patterns are differentially associated with functional ability and decline in a longitudinal cohort of older women | *age and* sex  45+   females only | FEMALE: i) Musculoskeletal ii) Neurological and Mental Health  iii) Cardiovascular |
| 40 | Investigation of multimorbidity and prevalent disease combinations in older Irish adults using network analysis and association rules | *age and* sex  50+ | MALE: i) Cardiovascular, Cataracts, Anemia  ii) Vision iii) other   FEMALE: i) Cardiovascular, Vision ii) Other |
| 119 | Multimorbidity patterns and their related characteristics in European older adults: A longitudinal perspective | *age and* sex  50+ | Males: i) Healthy ii) Metabolic iii) Articular, COPD, Ulcer iv) Severely Impaired  Females: i) Health ii) Metabolic iii) Osteoarticular  iv) Severely Impaired |
| 87 | Identifying multimorbidity clusters among Brazilian older adults using network analysis: Findings and perspectives | *age and* sex  60+ | MALE:  i) Cardiometabolic - Hypertension, Hypercholesterolemia, Obesity, Diabetes, Heart Disease ii) Respiratory - COPD, Asthma iii) Musculoskeletal-Renal - Low Back Pain, Arthritis, Rheumatism, Work Related MSK diseases, Kidney Disease iv) Mixed Mental Illness + Other - Depression, Mental Illness, Other Chronic Diseases v) Cancer   FEMALE:  i) Cardiometabolic - Hypertension, Hypercholesterolemia, Obesity, Diabetes, Heart Disease, Stroke, Kidney Disease, Depression  ii) Respiratory and Cancer - COPD, Asthma, Cancer iii) Musculoskeletal - Arthritis, Rheumatism, Work Related MSK diseases, Low Back Pain, iv) Mixed Mental Illness + Other - Depression, Mental Illness, Other Chronic Diseases v) Cancer |
| 14 | Multimorbidity Patterns in Elderly Primary Health Care Patients in a South Mediterranean European Region: A Cluster Analysis | age and sex  65-79 80+ | 65-79 years: FEMALE: i) Hypertensive diseases, Metabolic disorders ii) Musculoskeletal - Joint disorders, Soft tissue disorders, Bone density disorders  iii) Heart disease, Mood disorders, Thyroid gland disorders, Intestines diseases, Disorders of lens iv) Oral cavity diseases, Ear diseases, URT diseases, Dermatitis, Eczema,   MALE: i) Hypertensive diseases, Metabolic disorders, Male genital diseases, Diabetes, Obesity ii) Dorsopthies, Arthrosis, Soft tissue disorders, Oral cavity diseases, Joint disorders iii) Chronic lower respiratory diseases, Mental disorders due to psychoactive substance use iv) Heart disease, Ischemic heart disease  80+ years:  FEMALE: i) Hypertension, Metabolic, Arthrosis, Heart disease ii) Veins/lymph diseases, Lens disorders, bone density disorder, Dorsopathies, Joint iii) Diabetes, Obesity  iv) Obesity, GI diseases, Hernia   MALE: i) Hypertensive, Male genital, Metabolic, Heart, Arthrosis ii) Lens disorders, Dorsopathies, GI diseases, Hernia  iii) Ischemic heart disease, Renal failure Arterial diseases, Anemia iv) Obesity, Glaucoma, Inflammatory poly-arthropathies, External ear diseases |
| 15 | Multimorbidity patterns in the elderly: a prospective cohort study with cluster analysis | age and sex  65-79 80+ | 65-79 years: FEMALE: i) Unspecific ii) Musculoskeletal iii) Endocrine-Metabolic iv) Digestive v) Neuropsychiatric   MALE: i) Unspecific ii) Endocrine-Metabolic iii) Musculoskeletal iv) Digestive-Respiratory  v) Neuropsychiatric   80+ years:  FEMALE: i) Unspecific ii) Musculoskeletal iii) Neuropsychiatric iv) Endocrine-metabolic v) Cardiovascular  MALE: i) Unspecific ii) Endocrine-Metabolic iii) Musculoskeletal  iv) Digestive-Respiratory v) Neuropsychiatric |
| 137 | Patterns of multimorbidity in older medical patients (>= 65 years): And how they relate to mobility the first year after an acute admission | *age and* sex  65+ | FEMALE: i) Minimal Chronic Conditions ii) Cardiovascular  iii) Neurological and Vascular iv) Degenerative and Pulmonary v) Degenerative and Mental   MALE:  i) Minimal Chronic Conditions ii) Cardiovascular iii) Neurological and Vascular iv) Dementia and Parkinson's v) Mental Disorders |
| 61 | A Health Profile of Senior-Aged Women Veterans: A Latent Class Analysis of Condition Clusters | *age and sex*  65+  Females only | FEMALE only:  i) CVS  ii) Healthy  iii) Musculoskeletal  iv) Ophthalmologic v) Multimorbid |
| 18 | Multimorbidity patterns in the elderly: a new approach of disease clustering identifies complex interrelations between chronic conditions | *age and* sex  65+ | FEMALE: i) Cardiovascular, Metabolic  ii) Anxiety, Depression, Somatoform, Pain  iii) Neuropsychiatric   MALE: i) Anxiety, Depression, Somatoform, Pain  ii) Cardiovascular, Metabolic  iii) Neuropsychiatric |
| 19 | Patterns of multimorbidity and differences in healthcare utilization and complexity among acutely hospitalized medical patients (>=65 years) - a latent class approach | *Age and* sex  65+ | FEMALE: i) Minimal chronic conditions ii) Cardiovascular disorders  iii) Neurological, Vascular  iv) Degenerative, Pulmonary disorders, Osteoporosis, COPD v) Metabolic and cardiovascular disorders, CVS, Diabetes   MALE:  i) Minimal chronic conditions ii) Cardiovascular disorders  iii) Neurological, Vascular  iv) Degenerative, Pulmonary disorders  v) Metabolic and cardiovascular disorders |
| 52 | Association Rules Analysis of Comorbidity and Multimorbidity: The Concord Health and Aging in Men Project | *age and* sex  70+  males only | i) Vascular  ii) Metabolic  iii) Neurodegenerative  iv) Mental health and other v) Musculoskeletal and other |
| 126 | Multimorbidity patterns of and use of health services by Swedish 85-year-olds: an exploratory study | *age and* sex  85+ | MALES: i) Vascular ii) Cardiopulmonary iii) Somatic-Mental iv) Cardiac v) Malignancy and Osteoarthritis   FEMALES:  i) Vascular ii) Urinary Incontinence and Osteoarthritis  iii) Cardiopulmonary  iv) Malignancy and Thyroid  v) Mental |
| 2 | Multimorbidity among two million adults in China | age and sex   45-59 60+ | Association Rules Mining: MALE 45-59 years: i) Ischemic Heart Disease, Cerebrovascular Disease, Osteoarthritis  ii) Ischemic Heart Disease, Cerebrovascular Disease, Osteoarthritis iii) Ischemic Heart Disease, Osteoarthritis, Hypertension  iv) Ischemic Heart Disease, COPD, Osteoarthritis  v) Ischemic Heart Disease, Diabetes, Osteoarthritis  MALE 60+ years:  i) Cataract, Ischemic Heart Disease, Osteoarthritis  ii) Cataract, Ischemic Heart Disease, Glaucoma iii) Cataract, Hypertension, Glaucoma iv) Cataract, Osteoarthritis, Glaucoma v) Cataract, Glaucoma   FEMALE 45-59 years:  i) Ischemic Heart Disease, Cerebrovascular Disease, Osteoarthritis  ii) Ischemic Heart Disease, Cerebrovascular Disease, COPD iii) Ischemic Heart Disease, Cerebrovascular Disease, Osteoarthritis  iv) Ischemic Heart Disease, Cerebrovascular Disease, Osteoarthritis v) Ischemic Heart Disease, Cerebrovascular Disease, Hypertension   FEMALE 60+ years:  i) Cataract, Glaucoma ii) COPD, Osteoarthritis, Peptic Ulcer Disease ... (4 conditions total) iii) Ischemic Heart Disease, Ischemic Heart Disease, Depression ... (5 conditions total) iv) Ischemic Heart Disease, COPD, Depression v) COPD, Ischemic Heart Disease, CKD   HCA:  MALE: 45-59 years: i) Depression, Heart Failure, Cerebrovascular Disease  ii) Malignancy, Peptic Ulcer Disease, Ischemic Heart Disease  iii) Diabetes, CKD, Cataract   MALE 60+ years:  i) Depression, Malignancy ii) Peptic Ulcer Disease, COPD, Ischemic Heart Disease  iii) CKD, Diabetes iv) Cataract, Glaucoma   FEMALE4 5-59 years:  i) Malignancy, Depression, Cerebrovascular Disease  ii) Heart Failure, CKD, Diabetes  iii) Cataract, Glaucoma    FEMALE 60+ years:  i) Malignancy, Depression, Cerebrovascular Disease  ii) Heart Failure, CKD  iii) Cataract, Glaucoma |
| 123 | Multimorbidity Patterns in Hospitalized Older Patients: Associations among Chronic Diseases and Geriatric Syndromes | *age* and sex   65+ | MALE: I) Cardiovascular, COPD and prostate cancer  ii) Induced Dependency, Hearing loss  iii) Anemia and Falls iv) Osteoarticular, Depression and Constipation   FEMALE:  I) Cardiovascular ii) Induced Dependency iii) Falls, Depression, Pain, and Constipation  iv) Osteoarticular, Dysphagia and Pressure Ulcers |
| 72 | Comorbidity patterns in patients with chronic diseases in general practice | sex   14+ | MALE:  i) Benign Prostatic Hyperplasia, COPD  FEMALE:  i) Osteoporosis, Degenerative Joint Disease |
| 12 | Characteristics, service use and mortality of clusters of multimorbid patients in England: A population-based study | socioeconomic status and age  18-44 45-64 65-84 | *Multimorbidity overall was highest in the most deprived (31.4%), compared to the least deprived (27.1%*  Greatest Deprivation 18-44 years i) Alcohol, Depression  ii) Depression, Anxiety, Pain iii) Hearing loss, Depression, IBS  Greatest Deprivation 45-64 years i) Alcohol, Psychoactive Substance Misuse, Pain (57%) ii) Depression, Pain, Anxiety iii) Hypertension, Diabetes, Pain  Greatest Deprivation 65-84 years i) Pain, CHD, Depression (43%0 ii) COPD, Asthma, Pain |
| 24 | Multimorbidity patterns in low-middle and high income regions: a multiregion latent class analysis using ATHLOS harmonised cohorts | socioeconomic status and *age  50+* | Data is stratified by country, and each country belonged to a group of HICs or LMICs. They are in figure 1 and figure 2 of the paper, respectively. MM is common worldwide, especially in HIC's and Russia.   LMIC's such as China and India:  i) Respiratory-mental-articular |
| 29 | Prevalence and Patterns of Multimorbidity in a Nationally Representative Sample of Older Chinese: Results From the China Health and Retirement Longitudinal Study | socioeconomic status and sex and *age  50+* | *HCA Data Stratified By Sex and Rural vs Urban Setting*   Rural Women  i) Vision Impairment, Cancer, Hip Fracture, Arthritis  ii) Chornic Lung Disease, Liver Disease, Kidney Disease  iii) Hypertension, Stroke, Dyslipidemia   Urban Women i) Chronic Lung Disease, Liver Disease, Psychiatric  ii) Hypertension, Stroke, Dementia   Rural Men i) Hypertension, Stroke, Dyslipidemia  ii) Chronic Lung Disease, Vision Impairment, Liver Disease   Urban Men i) Cancer, Liver Disease, Kidney Disease  ii) Stroke, Psychiatric Condition, Memory Related Disease iii) Hypertension, Dyslipidemia, Diabetes  iv) Chronic Lung Disease, Hip Fracture, Stomach Disease |
| 72 | Comorbidity patterns in patients with chronic diseases in general practice | whole population   14+ | i) Cardiac Arrythmias, Hyperlipidemia, Hypertension, Diabetes ii) Ischemic Heart Disease, Cerebrovascular Diseases, Chronic Renal Failure, Congestive Heart Failure iii) Asthma, Thyroid, Anxiety and Depression, Schizophrenia  iv) Other |
| 46 | Social determinants of multimorbidity in Jamaica: application of latent class analysis in a cross-sectional study | whole population   15+ | i) Relatively Healthy class (With a single or no morbidity) ii) Metabolic - Obesity, Hypertension  iii) Vascular-inflammatory - Hypertension, Obesity, Diabetes, Hypercholesterolemia, Arthritis  iv) Respiratory - Asthma, COPD |
| 54 | Patterns of chronic multimorbidity in the elderly population | whole population   15+ | i) Circulatory - Hypertension, Heart failure, Chronic atrial fibrillation, CVD  ii) Cardiopulmonary- CVD, COPD iii) Mental diseases, Musculoskeletal disorder - Dementia, Depression, Hip fracture  iv) Diabetes mellitus, Visual impairment  v) Malignancy, Anemia |
| 152 | Similar multimorbidity patterns in primary care patients from two European regions: results of a factor analysis | whole population   15+ | i) Cardiometabolic ii) Mechanical iii) Psychiatric and Substance Abuse  iv) Depressive v) Psychogeriatric |
| 127 | Multimorbidity states associated with higher mortality rates in organ dysfunction and sepsis: a data-driven analysis in critical care | whole population   16+ | i) Cardiopulmonary ii) Uncomplicated Diabetes iii) Young iv) Hepatic v) Complicated Diabetes |
| 35 | Multimorbidity and health-related quality of life (HRQoL) in a nationally representative population sample: Implications of count versus cluster method for defining multimorbidity on HRQoL | whole population   16+ | i) CVD, Arthritis ii) Major depressive disorder, Anxiety |
| 17 | A Latent class analysis of multimorbidity and the relationship to socio-demographic factors and health-related quality of life. A national population-based study of 162,283 Danish Adults | whole population   16+ | i) Relatively Healthy with Allergies ii) Diabetes, Hypertension, Arthritis  iii) Musculoskeletal Disorders, Slipped discs, Hypertension, Osteoporosis  iv) Headache (migraine), Mental disorders v) Asthma-Allergy |
| 1 | Patterns of multimorbidity and pharmacotherapy: a total population cross-sectional study | whole population   18+ | i) Anxiety, Depression, Alcohol problems ii) Hypertension iii) Hypertension, Diabetes  iv) Cancer  v) Thyroid disorders |
| 6 | Identifying longitudinal clusters of multimorbidity in an urban setting: A population-based cross-sectional study (WC) | whole population   18+ | i) Mental Health, Anxiety, Depression ii) CVS - Heart failure, Atrial fibrillation, Chronic kidney disease  iii) Osteoarthritis, Cancer, Chronic pain, Hypertension  iv) Liver disease, Viral hepatitis  v) Alcohol/substance dependence, HIV |
| 26 | Lifestyle factors and high body mass index are associated with different multimorbidity clusters in the Brazilian population | whole population   18+ | i) Cardiometabolic diseases - Hypertension, Stroke, Hypercholesterolemia, Diabetes ii) Musculoskeletal diseases, Spinal diseases, Arthritis  iii) Cancer, Mental/occupational diseases  iv) Respiratory diseases - Asthma, COPD |
| 49 | Prevalence of multimorbidity in the adult population attending primary care in Portugal: A cross-sectional study | whole population   18+ | i) Overweight ii) Anxiety, Depression iii) Varicose veins, Back syndrome (without radiating pain) iv) Obesity, Non-insulin dependent diabetes, Uncomplicated hypertension v) Osteoarthrosis, Osteoporosis, Goitre, Back syndrome (with radiating pain)  ** does not show prevalence ** |
| 55 | Patterns of chronic physical multimorbidity in psychiatric and general population | whole population   18+ | i) Relatively healthy  ii) Musculoskeletal iii) Hypertension and obesity  iv) Complex multimorbidity |
| 68 | Chronic condition patterns in the US population and their association with health related quality of life | whole population   18+ | i) Healthy ii) Vascular Risk iii) Anxiety  iv) Heart Disease v) Severely Impaired |
| 70 | Clustering and Healthcare Costs With Multiple Chronic Conditions in a US Study | whole population   18+ | i) Metabolic Syndrome - Hypertension, Hypercholesterolemia, Diabetes ii) Age-related Disease - Osteoarthritis, Hypertension, High Cholesterol iii) Renal Failure - Renal Disease iv) Respiratory - Allergy, COPD v) Cardiovascular disease - Hypertension, CAD Cardiomyopathy, Hypercholesterolemia |
| 71 | Clustering Diagnoses From 58 Million Patient Visits in Finland Between 2015 and 2018 | whole population   18+ | i) Pregnancy  ii) Immune System and Blood-Forming Organs iii) Mixed - Mental Disorders, Malformations, and Ear/Oral Cavity Diseases  iv) Tumours  v) Lower Respiratory System |
| 80 | Epidemiology of multimorbidity within the Brazilian adult general population: Evidence from the 2013 National Health Survey (PNS 2013) | whole population   18+ | i) Cardiometabolic ii) Musculoskeletal-Mental iii) Respiratory |
| 101 | Multimorbidity and patterns of chronic conditions in a primary care population in Switzerland: a cross-sectional study | whole population   18+ | i) Cardiovascular Disease and Risk Factors - Cerebrovascular Disease, Cardiovascular DIsease, Ischemic Heart Disease  ii) General Age-Related Conditions, Metabolic Syndrome, Hearing problems, Osteoarthrosis, Hypertension  iii) Tobacco and Alcohol related dependencies and COPD  iv) General Pain, Musculoskeletal and Psychological - Asthma, Osteoporosis, Rheumatoid Arthritis |
| 103 | Multimorbidity clustering of the emergency department patient flow: Impact analysis of new unscheduled care clinics | whole population   18+ | i) Digestive, Pregnancy, mensturation ii) General Symptoms and Mental  iii) Infectious Diseases iv) General Chronic Disease v) Mental disorders and At-Risk Behaviours |
| 129 | Patterns and Consequences of Multimorbidity in the General Population: There is No Chronic Disease Management Without Rheumatic Disease Management | whole population   18+ | i) Chronic Noncommunicable Diseases - mixed bag ii) Cardiometabolic  iii) Respiratory - COPD, Asthma, Cancer iv) Depression and Rheumatic and Musculoskeletal |
| 131 | Patterns of chronic conditions and their association with visual impairment | whole population   18+ | I) Healthy ii) Hypertensive iii) Heart Disease iv) Severely Impaired |
| 142 | Patterns of patients with multiple chronic conditions in primary care: A cross-sectional study | whole population   18+ | i) Cardiometabolic, Ischemic Heart Disease, Kidney Disease, Anemia ii) Cardiometabolic, Ischemic Heart Disease iii) Cardiometabolic, Obesity iv) Cardiometabolic, Arthritis  v) Cardiometabolic, Arthritis |
| 154 | The association between clusters of chronic conditions and psychological well-being in younger and older people-A cross-sectional, population-based study from the Lolland-Falster Health Study, Denmark | whole population   18+ | i) Cardiovascular, Endocrine, Kidney, Musculoskeletal, Cancer ii) Mental, Lung, Neurological, GI, Sensory |
| 158 | Use of Latent Class Analysis and k-Means Clustering to Identify Complex Patient Profiles | whole population   18+ | LCA:  i) Less Engaged ii) Older with CVD iii) Frail Elderly iv) Chronic Pain v) Psychiatric Illness  K-Means Clustering:  i) Older with CVD ii) Psychiatric iii) Highest Acuity in Frail Elderly iv) Least Engaged v) Pain Management |
| 159 | Using item response theory with health system data to identify latent groups of patients with multiple health conditions | whole population   18+ | i) Complex Diabetes ii) Complex Mental heatlh  iii) Substance Use  iv) Cancer and Cardiac v) Liver Disease |
| 108 | Multimorbidity in adults from a southern Brazilian city: occurrence and patterns | whole population   20+ | i) Cardiometabolic and Joint Problems and Osteoporosis  ii) Respiratory Diseases - Asthma, Bronchitis, Emphysema |
| 148 | Prevalence and Patterns of Multi-Morbidity in Serbian Adults: A Cross-Sectional Study | whole population   20+ | i) Non-Communicable ii) Cardiometabolic iii) Respiratory iv) Cardiovascular v) Aggregate |
| 160 | Prevalence, patterns of multimorbidity and associations with health care utilization among middle-aged and older people in China | Age   45+ | Age 45+ i) Relatively healthy  ii) Respiratory iii) Stomach-arthritis iv) Vascular |
| 161 | Multimorbidity patterns in the German general population aged 40 years and over | Age  43-92 | Age 43+ i) healthy  ii) high morbidity iii) arthrosis/inflammatory/mental illness iv) hypertension-metabolic  v) cardiovascular/cancer |
| 162 | Multimorbidity patterns in South Africa: A latent class analysis | Whole population   15+ | i) HIV, Hypertension and Anemia ii) Anemia and Hypertension  iii) Cardiovascular-related  iv) Diabetes and Hypertension |
| 163 | Multimorbidity patterns and the association with health status of the oldest-old in long-term care facilities in China: a two-step analysis | age   60+ | ‘Younger’-old: i) Cardiovascular-neurological  ii) Metabolic-endocrine-neurological-cardiovascular iii) Orthopedic-cardiovascular-metabolic-endocrine-neurological iv) Cardiovascular-metabolic-endocrine   ‘Oldest’-old: i) Cardiovascular-respiratory-digestive-urogenital ii) Neurological-cardiovascular-metabolic-endocrine iii) Orthopedic-cardiovascular-metabolic-endocrine iv) Cardiovascular-metabolic-endocrine |
| 164 | Multimorbidity Clusters in the Oldest Old: Results from the EpiChron Cohort | age   80+ | FEMALE 80+  i) Endocrine–metabolic ii) Cardiovascular iii) Osteoarthritis iv) Delirium and dementia   MALE 80+ i) Cardiometabolic ii) Genitourinary, dementia, ictus, chronic skin ulcers iii) Cancer and COPD  FEMALE 90+  i) Delirium-dementia ii) Hypertension  iii) Ictus without delirium  iv) Chronic skin ulcers v) Genitourinary disorders  MALE 90+ i) Cardiovascular ii) Endocrine-metabolic iii) Genitourinary, delirium, ictus iv) Cancer-COPD  FEMALE 100+ i) Ulcers  iii) Delirium, endocrine, diabetes and hypertension iii) Osteoarthritis and visual deficits  iv) Congestive heart failure  v) Genitourinary and ictus   MALE 100+ i) Genitourinary and chronic skin ulcers ii) Cardiovascular, thyroid, delirium and dementia iii) Hypertension |
| 165 | Gender and Socioeconomic Differences in the Prevalence and Patterns of Multimorbidity among Middle-Aged and Older Adults in China | Age 45+ | i) Relatively healthy class ii) Respiratory class iii) Stomach-arthritis class iv) Vascular class |
| 166 | Chronic Condition Patterns in the US Population and Their Association with Health Related Quality of Life | whole population   18+ | i) Healthy ii) Vascular risk iii) Anxiety iv) heart disease v) severely-impaired |
| 167 | Clustering long-term health conditions among 67728 people with multimorbidity using electronic health records in Scotland | age   25+ | i) Alcohol misuse ii) Cancer iii) Obesity iv) Renal failure v) Heart failure |
| 167 | Clustering long-term health conditions among 67728 people with multimorbidity using electronic health records in Scotland | age | 44-49 years  i) Alcohol misuse ii) Cancer iii) Obesity  50-59 years i) Alcohol misuse ii) Cancer iii) Obesity iv) Renal failure v) Heart failure  60-69 years  i) Alcohol misuse ii) Cancer iii) Obesity iv) Renal failure v) Heart failure  70-79 years  i) Alcohol misuse ii) Cancer iii) Obesity iv) Renal failure v) Heart failure vi) Other   80+  i) Alcohol misuse ii) Cancer iii) Renal failure iv) Other |
| 167 | Clustering long-term health conditions among 67728 people with multimorbidity using electronic health records in Scotland | age and sex  25+ | FEMALES: i) Alcohol misuse ii) Cancer iii) Obesity iv) Renal failure v) Heart failure  MALES: i) Alcohol misuse ii) Cancer  iii) Obesity iv) Renal failure v) Heart failure |
| 167 | Clustering long-term health conditions among 67728 people with multimorbidity using electronic health records in Scotland | age and socioeconomic status   25+  most and least deprived | Most deprived  i) Alcohol misuse ii) Cancer iii) Obesity iv) Renal failure v) Heart failure  Least deprived  i) Alcohol misuse ii) Cancer iii) Obesity iv) Renal failure v) Heart failure |

*Footnote: Clustering Technique Abbreviations*

*LCA - Latent class analysis; HCA - Hierarchical cluster analysis, MCA - Multiple correspondence analysis; EFA - Exploratory factor analysis; IBS – Irritable Bowel Syndrome; COPD – Chronic obstructive lung disease; CHD – Coronary Heart Disease; CKD – Chronic Kidney Disease; MI – Myocardial Infarction; CVS – Cardiovascular system; MM – Multimorbidity; MSK – Musculoskeletal; CNS – Central Nervous System; GI – Gastrointestinal; AF – Atrial fibrillation; HIC – High-income countries; LMIC – Low and medium-income countries*
