# Supplementary material for: Identifying clusters of multimorbid disease and differences by age, sex, and socioeconomic status: A systematic review
Source: PLoS One. 2025 Aug 22;20(8):e0329794. doi: 10.1371/journal.pone.0329794 (PMC12373218; doi:10.1371/journal.pone.0329794)
Supplement: S4 Table — (DOCX) [file pone.0329794.s008.docx]

### **Supplementary Table 4. Broad Cluster Groupings identified across papers and stratified by Age and Sex.**

| **18+** | |
| --- | --- |
| Cluster title (and paper #) | Count of clusters reported in papers |
| **Cardiometabolic**  Cardiometabolic diseases (26) Cardiovascular Disease, Hypertension, CAD Cardiomyopathy, Hypercholesterolemia (70) Cardiometabolic (80, 129) Cardiometabolic, Ischemic Heart Disease, Kidney Disease, Anaemia (142) Cardiometabolic, Ischemic Heart Disease (142) Cardiometabolic, Obesity (142) Cardiometabolic, Arthritis (142, 142) | 9 |
| **Diabetes- Hypertension** Hypertension (1, 131) Hypertension, Diabetes (1) Obesity, Non-insulin dependent diabetes, Uncomplicated hypertension (49) Hypertension and obesity (55) Metabolic Syndrome (70) Complex Diabetes (159) | 7 |
| **Mental Health - Anxiety, Depression** Anxiety, Depression, Alcohol problems (1) Mental Health (6) Anxiety, Depression (49) Anxiety (103) General Symptoms and Mental (159) Complex Mental health (166) | 7 |
| **Cardiovascular**  CVS Heart Disease (6) *Cardiovascular Disease and Risk Factors (68) Heart Disease (101. 166) Older with CVD (158) | 7 |
| **Musculoskeletal-Neurological-Mental** Musculoskeletal-Mental (80) General Pain, Musculoskeletal and Psychological (101) Depression and Rheumatic and Musculoskeletal (129) Mental, Lung, Neurological, GI, Sensory(154) Chronic Pain (158) | 5 |
| **Age-related - Degenerative**  Age-related Disease , Osteoarthritis, Hypertension, High Cholesterol (70) General Age-Related Conditions and Metabolic Syndrome (101) Frail Elderly (158) Highest Acuity in Frail Elderly (158) | 4 |
| **Respiratory - Asthma, COPD etc.** Respiratory diseases (26, 80)  Lower Respiratory System (71) Respiratory - COPD, Asthma, Cancer (129) | 4 |
| **Dependence - Substance abuse, HIV** Dependence - Alcohol/substance dependence, HIV (6) Tobacco and Alcohol related dependencies and COPD (101) Mental disorders and At-Risk Behaviours (103) Substance Use (159) | 4 |
| **Musculoskeletal** Musculoskeletal diseases (26, 55) Osteoarthrosis, Osteoporosis, Goitre, Back syndrome (49) | 3 |
| **Cancer**  Cancer (1) Cancer, Mental/occupational diseases (26) Tumours (71) | 3 |
| Psychiatric (158, 158) | 2 |
| **Pregnancy**  Pregnancy (71) Digestive, Pregnancy, menstruation (103) | 2 |
| Liver (6, 159) | 2 |
| Overweight (49) | 1 |
| Relatively healthy (55) | 1 |
| Healthy (68) | 1 |
| Chronic Noncommunicable Diseases (129) | 1 |
| Healthy (131) | 1 |
| Cardiovascular, Endocrine, Kidney, Musculoskeletal, Cancer (154) | 1 |
| Less Engaged (158) | 1 |
| Vascular Risk (68, 166) | 2 |
| Immune System and Blood-Forming Organs (71) | 1 |
| Osteoarthritis, Cancer, Chronic pain, Hypertension (6) | 1 |
| Varicose veins, Back syndrome (without radiating pain) (49) | 1 |
| **Renal Disease** Renal Failure (70) | 1 |
| Mixed (71) | 1 |
| Infectious Diseases (103) | 1 |
| Complex multimorbidity (55) | 1 |
| **Asthma-Allergy-Hypersensitivity**  Asthma, COPD, Eczema, IBS, Allergies - Respiratory (70) | 1 |
| General Chronic Disease (103) | 1 |
| Severely Impaired (131, 166, 68) | 3 |
| Least Engaged (158) | 1 |
| Cancer and Cardiac (159) | 1 |
| **Endocrine**  Thyroid disorders (1) | 1 |
| Pain Management (158) | 1 |
| Healthy (166) | 1 |

| **45+** | |
| --- | --- |
| Cluster title (and paper #) | count |
| **Cardiometabolic - CVS disease, Diabetes, Hypertension, CKD** Diabetes, Parkinsons, Heart Failure, 5 conditions total) (28) Chronic pain, Heart Failure, Hypertension (28) Heart Failure, Hypertension, Chronic Pain (28) Atrial Fibrillation, Chronic Kidney Disease, Chronic Pain ... (4 conditions total) (28) Atrial Fibrillation, Chronic Kidney Disease, MI .. (4 conditions total) (28) Cardiometabolic - CVS disease, Diabetes, Impaired Glucose Tolerance (91) Complex Cardiometabolic Disorders (28) | 7 |
| **Metabolic- Diabetes, Hypertension, High cholesterol, Obesity**  Concordant - Hypertension, Diabetes (50) Metabolic Disorders - Hypertension, Diabetes, Hypertension (88) Hypertension (88) | 3 |
| **Asthma-Allergy-Hypersensitivity - Asthma, COPD, Eczema, IBS, Allergies** Pulmonary, Digestive, Rheumatic (63) Respiratory - Asthma, Bronchitis, Emphysema, Breathing Difficulties (91) Chronic Pulmonary Disease, IBS, Peripheral Vascular Disease ... (6 conditions total) (28) | 3 |
| **Cancer (2+ cancers)** Lymphoma, Metastatic Cancer, Cirrhosis ... (9 conditions total) (28) Cancer (91) | 2 |
| **Neuroendocrine and Cardio:**  Hyperthyroidism, Epilepsy, Hypertension (28)  Cardiovascular, Endocrine, Neuro (63) | 2 |
| **Musculoskeletal-Mental - Musculoskeletal (Arthritis), Neurological, Mental, Vascular/circulatory, Cardiovascular, Functional and Sensory disorders**  Musculoskeletal - Arthritis, Joint pain, Back pain (91) GI Disorders, Hypertension, Musculoskeletal Disorders (88) | 2 |
| Cancer, Cirrhosis, Diabetes ... (9 conditions total) (28) | 1 |
| Minimal MM risk (50) | 1 |
| Discordant - Angina, Asthma, Chronic lung disease, Arthritis, Depression (50) | 1 |
| Low Morbidity (63) | 1 |
| High Morbidity (63) | 1 |
| **Neuropsychiatric / psychosomatic** Psychosomatic - Anxiety, Depression, Somatic symptoms (IBS, Headache, Migraine, Severe Fatigue) (91) | 1 |
| Relatively Healthy (88, 160) | 2 |
| Cancer, Peripheral Vascular Disease, Heart Failure ... (4 conditions total) (28) | 1 |
| Healthy (165) | 1 |
| Respiratory (160, 165) | 2 |
| Stomach-arthritis (160, 165) | 2 |
| Vascular (160, 165) | 2 |

| **50+** | |
| --- | --- |
| Cluster title (and paper #) | count |
| **Cardiometabolic** Cardio-metabolic (24, 36, 81, 157) Cardiovascular, Renal, Metabolic(78) Heart Disease, Stroke, Hypertension, Diabetes (97) Cardiometabolic Diseases, Cancer, and Others (8 conditions total) (116) Hypertension, Angina, Stroke, Diabetes, Cataracts, Arthritis, Edentulism (121) Complex cardiometabolic (16) | 9 |
| **Musculoskeletal-Neurological-Mental** Respiratory-mental-articular (24) Mental-articular (3 countries) (27) Cardiorespiratory, Mental, Arthritis (41) Musculoskeletal and Mental (81) Cardiorespiratory, Mental, Arthritis (90) Arthritis, Anxiety, Depression (121) | 6 |
| **Metabolic - Hypertension-Diabetes** Metabolic (27) Hypertension and diabetes (32, 97)  Diabetes, Glaucoma, Retinopathy and Macular Degeneration (116) | 4 |
| **Respiratory - Asthma, COPD** Respiratory (16, 27, 32) Respiratory - Asthma and COPD (116) | 4 |
| **Cardiorespiratory**  Cardio-respiratory (27) Cardiorespiratory - Angina, Asthma, Chronic Lung Disease (121) | 2 |
| **Asthma-Degenerative**  Asthma, Bronchitis, Depression, Arthritis, Osteoporosis (97) Asthma, Bronchitis, Arthritis, Osteoporosis, Depression (97) | 2 |
| **Vascular-Metabolic** Metabolic, Stroke (41, 90) | 2 |
| **Asthma-Allergy**  Arthritis, Asthma, Allergic rhinitis, Depression, Thyroid (36) Respiratory and Dermal (78) | 2 |
| **Mental Health**  Cognitive-emotional (29) Cognitively impaired (16) | 2 |
| **Musculoskeletal**  Musculoskeletal (81) Musculoskeletal - Spinal problems, Arthritis, Rheumatism, Osteoporosis (116) | 2 |
| **Digestive disease and Arthritis**  Stomach-arthritis (29) Skeletal, Articular, and Digestive (78) | 2 |
| Vascular-metabolic (29) | 1 |
| Healthy (16, 24, 32, 90) | 4 |
| Relatively healthy (36, 81, 157) | 3 |
| Minimum disease (41) | 1 |
| **Psychiatric**  Neuropsychic (78) | 1 |
| Age-associated chronic conditions (16) | 1 |
| Cancer (97) | 1 |
| Cancer, Heart Disease, Stroke (97) | 1 |
| Arthritis-Cataract (157) | 1 |
| Hepatorenal (29) | 1 |
| Complex and multisystem (32) | 1 |
| Neurodegenerative - Parkinsons and Alzheimer (116) | 1 |
| Multimorbidity Group (157) | 1 |
| Malignant, Digestive, Urologic (78) | 1 |
| Complex Multimorbidity (81) | 1 |

| **60+** | |
| --- | --- |
| Cluster title (and paper #) | count |
| **Cardiometabolic**  Cardiac, Respiratory, Hypertension (22, 53, 87, 100, 102, 37) Cardiometabolic (39) Cardiovascular diseases (dyslipidaemia, hypertension, coronary heart disease), Metabolic diseases (diabetes), Kidney disease (47) Cardiovascular diseases - Hypertension, Coronary heart disease (37) Coronary Atherosclerotic Heart Disease (118) Stroke, Heart Disease, Diabetes ... (6 conditions total) (130) | 10 |
| **Respiratory - Asthma-COPD** Lung and Asthma (39) Respiratory (53, 114) Asthma, Chronic Lung Disease (111) | 4 |
| **Musculoskeletal**  Musculoskeletal (87) Lower Limb Fractures (188) Arthritis (130, 145) | 4 |
| **Musculoskeletal/Degenerative and GI** Vascular, Upper gastrointestinal, Musculoskeletal (22) Degenerative, Digestive diseases, Arthritis, Hearing loss, Osteoporosis (37) Stomach, Arthritis (39, 114) | 4 |
| **Musculoskeletal- Neurological- Mental** Musculoskeletal, Mental, Functional disorders (53) Bones and Pain (102) | 2 |
| **Respiratory and GI** Digestive and Respiratory (100) GI and CNSLD (156) | 2 |
| Psychological, Neurological (22) Neuropsychiatric and Stroke (102) | 2 |
| Respiratory and Cancer (87) Lung and Cancer (102) | 2 |
| **Cancer**  Neoplasia (22) sensory Impairments and Cancer (113) | 2 |
| Mixed Mental Illness + Other (87) Depression (130) | 2 |
| Hypertension, Arthritis (39) | 1 |
| Cerebrovascular, Metabolic (51) | 1 |
| **Degenerative (100)** | 1 |
| Unspecific (113) | 1 |
| Relatively Healthy (114) | 1 |
| Other Diseases (118) | 1 |
| Low Risk (156) | 1 |
| Degenerative diseases (hearing disorder, cataract, joint disease), Cancer (47) | 1 |
| Cardiopulmonary disorders, Mental, Degenerative disorders (51) | 1 |
| Asthma, Arthritis, Rheumatism, Chronic Lung Disease (111) | 1 |
| MSK, Respiratory, and GI (113) | 1 |
| Vascular (114) | 1 |
| Vision Problems, Hearing Problems, Problems with Teeth and Gums (13) | 1 |
| Hypertension and Peptic Acid Disease (145) | 1 |
| Endocrine, Renal ( 22) | 1 |
| **Cardiovascular (156)** | 1 |
| **Diabetes-Hypertension (37)** | 1 |
| Hepatorenal/ Multisystem (39) | 1 |
| Liver disease, Lung disease, Gastrointestinal disease (47) | 1 |
| Dyslipidaemia, Hypertension, Arthritis and Rheumatism, MI (111) | 1 |
| All other conditions (145) | 1 |
| Metabolic and Sleep Disorders (113) | 1 |
| Multiple Risks (156) | 1 |
| CVS, Anaemia, and Dementia (113) | 1 |
| *Multisystem Morbidity (114)* | 1 |

| **65+** | |
| --- | --- |
| Cluster title (and paper #) | count |
| **Cardiometabolic** Heart Disease, Hypertension (62) Metabolic and cardiovascular disorders (19) Cardiovascular disease, Diabetes, Hypertension (38) Diabetes, CHD, Dyslipidaemia (45) Dyslipidaemia, Cardiovascular Diseases (60) Diabetes, Hypertension, Cardiovascular Diseases (60) Hypertension, Heart Disease, Dyslipidaemia and Diabetes ... (5 conditions total)(93) Cardiometabolic (98, 112, 138) Heart Failure, Cerebrovascular, Diabetes, Hypertension, MI (110) Cardiovascular - Ischemic Heart Disease, Diabetes, Hypertension, Hyperlipidaemia (112) Hypertension and Coronary Heart Disease (147) Diabetes, Coronary Heart Disease and Hypertension (147) | 14 |
| **Musculoskeletal-Neurological-Mental**  Genitourinary, Mental, Musculoskeletal (5) Major Neurologic Disease - Alzheimer's, Parkinsons, Psychiatric (89) Sensory and Bone (98) Mental-Sensory (112) Neurological and Mental - Alzheimer’s, Cerebrovascular, Depression (122) Mental and Neurological (138) | 6 |
| **Diabetes-Hypertension** Diabetes, High cholesterol (9) Dyslipidaemia, Hypertension (62) Diabetes, Hypertension (62, 147) Hypertension (69) Diabetes and Coronary Heart Disease (147) | 6 |
| **Cardiovascular** Heart disease (9) Cardiovascular disorders (19) Cardiac - Congestive Heart Failure, Fibrillation (122) Cardiovascular (123, 153) | 5 |
| **Musculoskeletal**  Osteoarticular (56, 123) Osteoporosis (67) Musculoskeletal (153, 15) | 5 |
| **Neuropsychiatric** Neurological, Functional, Sensory disorders (74) Cognitive/Affective (67) Neuropsychiatric (98) Neurological (153) Neuropsychiatric (15) | 5 |
| **Hypertension and Arthritis**  Arthritis, Hypertension (62, 69) Hypertension, Rheumatoid Arthritis, Vision Impairment (60, 60) | 4 |
| **Endocrine and Metabolic**  Hypertension, Thyroid dysfunction, AF (45) Endocrine and Metabolic (153, 15) | 3 |
| **Mental and GI**  Mental, Digestive, Blood (5) Mental, Nervous, Digestive (5) Constipation, Arthritis, CORD and Depression ... (22 conditions total) (93) | 3 |
| **Degenerative - Age-Related**  Degenerative, Lifestyle, Mental disorders (58) Dementia and Cognitive impairment, Urinary Incontinence, Osteoporosis (4 conditions total)(93) Falls (123) | 3 |
| **Cancer** Cancer and Metastases (110) Cancer (112) | 2 |
| **Respiratory-Musculoskeletal**  Back disease, Asthma, Allergy (9) COPD, Lung Disease, Rheumatism and Osteoporosis (110) | 2 |
| **Metabolic, Pulmonary and CVS** Metabolic, Pulmonary, Cardiovascular (58) Hypertension, Respiratory Diseases, Cardiovascular Diseases (60) | 2 |
| Digestive, Respiratory (153, 15) | 2 |
| **Vascular** Vascular (67) Vascular - Hypertension, Diabetes, Stroke (89) | 2 |
| **Respiratory - Asthma and COPD** COPD and asthma (69, 122) | 2 |
| Minimal chronic disease (9. 19, 38, 58, 67, 89) | 6 |
| Stroke, Hypertension (62) | 1 |
| Non-specified (5, 15) | 2 |
| Liver cirrhosis, Malignancy (45) | 1 |
| Cardiorespiratory, COPD, Heart failure, Cardiac arrythmia 56) | 1 |
| Low Chronic Disease (69) | 1 |
| Heart Disease, Cerebrovascular Disease, Arrythmias ... (6 conditions total) (93) | 1 |
| Osteoarticular disease (38) | 1 |
| Minor chronic disease (58) | 1 |
| Non-Vascular - Cancer, Osteoporosis, Arthritis ... (6 conditions total) (89) | 1 |
| Cardiovascular, Stroke, Cancer - Chronic Heart Failure, Coronary Heart Disease, Arrythmia, Stroke (89) | 1 |
| Induced Dependency (123) | 1 |
| Joint, Liver, Lung, Eye (138) | 1 |
| Nervous, Musculoskeletal, Circulatory (female dominant) (5) | 1 |
| Neurological, Vascular (19) | 1 |
| Psychogeriatric (56) | 1 |
| Multiple Morbidity (67) | 1 |
| Visual Impairment (69) | 1 |
| Cancer, Lung and GI (98) | 1 |
| Inflammatory-Digestive (112) | 1 |
| Degenerative, Pulmonary disorders (19) | 1 |
| High multisystem morbidity (38) | 1 |
| CVD, Dementia, Arthritis (45) | 1 |
| Other (112) | 1 |
| GI diseases, Cancer (138) | 1 |
| Many diseases (9) | 1 |
| Neuropsychiatric disease (38) | 1 |
| Anaemia, Gastric diseases, Gut diseases, Anxiety (45) | 1 |
| Arrythmias, Heart Failure, Breast Cancer ... (5 conditions total) (93) | 1 |

| **Female 45-64** | |
| --- | --- |
| Cluster title (and paper #) | count |
| **Cardiometabolic**  Cardiometabolic (148, 152) Hypertensive diseases, Diabetes mellitus, Other forms of heart disease... (7 conditions total) (10) | 3 |
| **Musculoskeletal** Mechanical (152) Arthrosis, Disorders of bone density and structure, Nerve root plexus disorders, Spondylopathies, Deforming dorsopathies (11) Other dorsopathies, Other soft tissue disorders, Arthrosis ... (4 conditions total) (10) | 3 |
| **Diabetes-Hypertension** Metabolic disorders, Hypertensive diseases, Obesity and other hyperalimentation (10) Hypertensive diseases, Obesity and other hyperalimentation, Diabetes mellitus (10) | 2 |
| Depression, Pain, Anxiety (12) | 1 |
| Metabolic disorders, Hypertensive diseases, Mental/behavioural disorders (psychoactive substance use), Dorsopathies, Soft tissue disorder (11) | 1 |
| Other dorsopathies, Neurotic, stress-related and somatoform disorders, Other soft tissue disorders ... **(10)** | 1 |
| Respiratory and Depression (148) | 1 |
| IBS, Hearing loss, Pain (12) | 1 |
| Asthma, Pain, COPD (148) | 1 |
| Cardiovascular and Depression (148) | 1 |
| Benign neoplasms, Dermatitis, Eczema, Mycoses, Nutritional anaemias, Breast disorders, Visual blindness, Skin appendages, Viral infections, Ear, Eye (11) | 1 |
| Mental and behavioural disorders due to psychoactive substance use, Disorders of thyroid gland, Mood [affective] disorders ... (4 conditions total)(10) | 1 |
| Acute upper respiratory infections, Diseases of oral cavity, salivary glands and jaws, Dermatitis (10) | 1 |
| Acute upper respiratory infections, Other diseases of urinary system, Diseases of external ear ... (9 conditions total)(10) | 1 |
| Diseases of oesophagus, stomach and duodenum, Episodic and paroxysmal disorders, Other diseases of intestines ... (4 conditions total) (10) | 1 |
| Dermatitis and eczema, Mycoses, Disorders of skin appendages ... (7 conditions total) (10) | 1 |

| **Male 45-64** | |
| --- | --- |
| Cluster title (and paper #) | count |
| **Cardiovascular** Cardiovascular - MI, Coronary Heart Disease, Hyperlipidaemia (148)  I30 -I52:Other forms of heart disease 6.9 43.7 I45 Other conduction disorders 0.77 (0.73 -0.82) I20 -I25:Ischaemic heart diseases 5.0 31.0 I25 Chronic ischaemic heart disease I70 -I79:Diseases of arteries, arterioles and capillaries ... (6 conditions total) (10) Hypertensive diseases, Other forms of heart disease, Ischaemic heart diseases ... (6 conditions total) (10) | 3 |
| **Diabetes- Hypertension**  Metabolic disorders, Hypertensive diseases, Mental/behavioural disorders (psychoactive substance use), Dorsopathies, Soft tissue disorder(11) Metabolic disorders, Hypertensive diseases, Obesity and other hyperalimentation ... (4 conditions total) (10) | 2 |
| **Cardiometabolic** Cardiometabolic - Hypertension, Obesity, Diabetes, Hyperlipidaemia (148) Cardiometabolic - Hypertension, Hyperlipidaemia, Obesity (152) | 2 |
| **Substance abuse** Metabolic disorders, Mental and behavioural disorders due to psychoactive substance use, Hypertensive diseases ... (6 conditions total) (10) Mental and behavioural disorders due to psychoactive substance use , Diseases of liver, Viral hepatitis ... (4 conditions total) (10) | 2 |
| **Musculoskeletal**  Mechanical (152) Other dorsopathies, Arthrosis, Spondylopathies ... (4 conditions total) (10) | 2 |
| Mental/behavioural disorders, Chronic lower respiratory diseases, Mood disorders, Viral hepatitis, Liver disease (11) | 1 |
| M70-M79:Other soft tissue disorders 16.9 29.3 M75 Shoulder lesions 0.87 (0.84-0.90) N40-N51:Diseases of male genital organs 12.1 21.0 N40 Hyperplasia of prostate M20-M25:Other joint disorders ... (10) | 1 |
| Respiratory - Asthma, COPD, Bronchitis, (148) | 1 |
| Aggregate - Joint disease, Depression, CKD, Stroke, Malignancy (148) | 1 |
| iGi diseases, Hernia, Genital diseases, Veins/lymph, Benign neoplasms, Upper respiratory tract, Urolithiasis (11) | 1 |
| F40 -F48:Neurotic, stress - related and somatoform disorders 13.5 24.9 F41 Nutritional marasmus 0.79 (0.74 -0.84) K00 -K14:Diseases of oral cavity, salivary glands and jaws 12.0 22.3 K02 Dental caries J40 -J47:Chronic lower respiratory diseases ... (10 conditions total) (10) | 1 |
| H90 -H95:Other disorders of ear 7.7 30.6 H91 Disorders of sclera and cornea in diseases classified elsewhere 0.87 (0.83 -0.91) H53 -H54:Visual disturbances and blindness 3.9 15.5 H54 Blindness and low vision B00 -B09:Viral infections characterized by skin and 3.5 13.9 B07 Viral warts mucous membrane lesions ... (9 conditions total) (10) | 1 |
| Dermatitis and eczema, Mycoses, Visual disturbances ... (6 conditions total) 2.5% (10) | 1 |

| **Female 65+** | |
| --- | --- |
| Cluster title (and paper #) | count |
| **Cardiovascular** MI, Coronary Heart Disease, Stroke, CKD (148)  Cardiovascular diseases (137, 61, 19, 123) | 5 |
| **Cardiometabolic** Cardiometabolic (148, 152, 18, 19) | 4 |
| **Degenerative, Frailty and Mental**  Degenerative and Mental (137) Falls - Falls and Depression, Pain, and Constipation (123) Psycho-geriatric (152) | 3 |
| **Musculoskeletal** Mechanical (152) Musculoskeletal (61) | 2 |
| **Neurological-Vascular**  Neurological and Vascular (137) Neurological, Vascular (19) | 2 |
| Degenerative and Pulmonary (137) Degenerative, Pulmonary disorders (19) | 2 |
| Respiratory **(**148) | 1 |
| Minimal Chronic Conditions (19, 137) | 2 |
| Healthy (61) | 1 |
| Anxiety, Depression, Somatoform, Pain (18) | 1 |
| Induced Dependency (123) | 1 |
| Neuropsychiatric (18) | 1 |
| Mechanical, Mental, Metabolic (148) | 1 |
| Depressive (152) | 1 |
| Ophthalmologic (61) | 1 |
| Osteoarticular - Dysphagia and Pressure Ulcers included (123) | 1 |
| Multimorbid (61) | 1 |

| **Male 65+** | |
| --- | --- |
| Cluster title (and paper #) | count |
| **Cardiovascular**   Cardiovascular - Hyperlipidaemia, MI, Coronary Heart Disease, Malignancy, Stroke (148) Cardiovascular (137, 19) Cardiovascular, Metabolic (18) Cardiovascular - COPD and prostate cancer included (123) | 5 |
| **Cardiometabolic** Cardiometabolic and Coronary Heart Disease (148) Cardiometabolic (152) Metabolic and cardiovascular disorders (19) | 3 |
| **Geriatric - Degenerative and Cognitive impairment**  Psycho-Geriatric (152) Dementia and Parkinson's (137) | 2 |
| Neurological and Vascular (137, 19) | 2 |
| Mechanical, Mental and Metabolic (148) Osteoarticular - Depression and Constipation included (123) | 2 |
| Respiratory - Chronic Bronchitis, COPD, Emphysema, Asthma (148) | 1 |
| Minimal Chronic Conditions (137) | 1 |
| Anxiety, Depression, Somatoform, Pain (18) | 1 |
| Minimal chronic conditions (19) | 1 |
| Induced Dependency - Hearing loss included (123) | 1 |
| Mechanical (152) | 1 |
| Neuropsychiatric (18) | 1 |
| Falls - Anaemia and Falls (123) | 1 |
| Degenerative, Pulmonary disorders (19) | 1 |
| **Psychiatric** Disorders (1) | 1 |
