# Supplementary material for: Identifying clusters of multimorbid disease and differences by age, sex, and socioeconomic status: A systematic review
Source: PLoS One. 2025 Aug 22;20(8):e0329794. doi: 10.1371/journal.pone.0329794 (PMC12373218; doi:10.1371/journal.pone.0329794)
Supplement: S5 Table — (DOCX) [file pone.0329794.s009.docx]

### **Supplementary Table 5. Bias Screening.**

| **Title** | 1) Representativeness of the exposed cohort | 2) Selection of the non exposed cohort | 3) Ascertainment of exposure | 4) Demonstration that outcome of interest was not present at start of study | 1) Comparability of cohorts on the basis of the design or analysis | 1) Assessment of outcome | 2) Was follow-up long enough for outcomes to occur | 3) Adequacy of follow up of cohorts | Total | Quality (>7☆ =high) |
| --- | --- | --- | --- | --- | --- | --- | --- | --- | --- | --- |
| Forslund et al. 2021 ([42](#_ENREF_42)) | a ☆ | a ☆ | a ☆ | a ☆ | n/a | a ☆ | b | d | 5 | low |
| Wang et al. 2020 ([43](#_ENREF_43)) | a ☆ | a ☆ | a ☆ | a ☆ | a ☆ | a ☆ | b | d | 6 | low |
| Violan et al. 2019  ([44](#_ENREF_44)) | a ☆ | a ☆ | a ☆ | a ☆ | n/a | a ☆ | b | d | 5 | low |
| Bisquera et al. 2021 (daisy-chained) ([11](#_ENREF_11)) | a ☆ | a ☆ | a ☆ | a ☆ | n/a | a ☆ | a ☆ | c | 6 | low |
| Moller et al. 2020 ([45](#_ENREF_45)) | a ☆ | a ☆ | a ☆ | a ☆ | n/a | a ☆ | b | d | 5 | low |
| Roso-Llorach et al. 2018 ([46](#_ENREF_46)) | a ☆ | a ☆ | a ☆ | a ☆ | n/a | a ☆ | b | d | 5 | low |
| Violan et al. 2018 ([47](#_ENREF_47)) | a ☆ | a ☆ | a ☆ | a ☆ | n/a | a ☆ | b | d | 5 | low |
| Zhu et al. 2019 ([48](#_ENREF_48)) | a ☆ | a ☆ | a ☆ | a ☆ | a ☆, b ☆ | a ☆ | b | d | 7 | high |
| Foguet-Boreu et al. 2019 ([49](#_ENREF_49)) | a ☆ | a ☆ | a ☆ | a ☆ | a ☆ | a ☆ | b | d | 6 | low |
| Guisado-Clavero et al. 2018 ([50](#_ENREF_50)) | a ☆ | a ☆ | a ☆ | a ☆ | a ☆ | a ☆ | b | d | 6 | low |
| Zheng et al. 2021 ([51](#_ENREF_51)) | b ☆ | a ☆ | c | a ☆ | n/a | b | b | d | 3 | low |
| Larsen et al. 2017 ([52](#_ENREF_52)) | b ☆ | a ☆ | c | a ☆ | n/a | b | b | d | 3 | low |
| Schafer et al. 2010 ([53](#_ENREF_53)) | c | a ☆ | a ☆ | a ☆ | n/a | a ☆ | b | d | 4 | low |
| Juul-Larsen et al. 2020 ([53](#_ENREF_53)) | a ☆ | a ☆ | a ☆ | a ☆ | n/a | a ☆ | b | d | 5 | low |
| Holden et al. 2011  ([54](#_ENREF_54)) | c | a ☆ | c | a ☆ | n/a | b | b | d | 2 | low |
| Mino-Leon et al. 2017 ([54](#_ENREF_54)) | a ☆ | a ☆ | a ☆ | a ☆ | n/a | a ☆ | b | d | 5 | low |
| Matesanz- ([55](#_ENREF_55)) | a ☆ | a ☆ | a ☆ | a ☆ | n/a | a ☆ | b | d | 5 | low |
| Bayes-Marin et al. 2020 ([56](#_ENREF_56)) | b ☆ | a ☆ | c | a ☆ | a ☆ | b | b | d | 4 | low |
| Hernandez et al. 2021 ([57](#_ENREF_57)) | c | a ☆ | c | a ☆ | n/a | b | b | d | 2 | low |
| De Carvalho et al. 2018 ([58](#_ENREF_58)) | b ☆ | a ☆ | c | a ☆ | n/a | b | b | d | 3 | low |
| Garin 2016 (Daisy-Chained) ([12](#_ENREF_12)) | b ☆ | a ☆ | c | a ☆ | n/a | b | b | d | 3 | low |
| Lai et al. 2021 ([59](#_ENREF_59)) | a ☆ | a ☆ | a ☆ | a ☆ | n/a | a ☆ | b | d | 5 | low |
| Yao et al. 2020 ([60](#_ENREF_60)) | b ☆ | a ☆ | c | a ☆ | a ☆ | b | a ☆ | d | 5 | low |
| Quinones et al. 2021 ([61](#_ENREF_61)) | b ☆ | a ☆ | c | a ☆ | n/a | b | a ☆ | b ☆ (<12% loss) | 5 | low |
| Ronaldson et al. ([62](#_ENREF_62)) | b ☆ | a ☆ | c | a ☆ | n/a | b | a ☆ | d | 4 | low |
| Khorrami et al. 2019 ([63](#_ENREF_63)) | b ☆ | a ☆ | c | a ☆ | n/a | b | b | d | 3 | low |
| Wang et al. 2017 ([64](#_ENREF_64)) | b ☆ | a ☆ | c | a ☆ | n/a | b | b | d | 3 | low |
| Park et a. 2019 ([65](#_ENREF_65)) | b ☆ | a ☆ | c | a ☆ | n/a | b | b | d | 3 | low |
| Lu et al. 2021([66](#_ENREF_66)) | c | a ☆ | b ☆ | a ☆ | n/a | a ☆ | b | d | 4 | low |
| Nguen et al. 2019 ([67](#_ENREF_67)) | c | a ☆ | c | a ☆ | n/a | b | a ☆ | c (loss 32%) | 3 | low |
| Liu et al. 2021([68](#_ENREF_68)) | b ☆ | a ☆ | c | a ☆ | n/a | b | a ☆ | a ☆ | 5 | low |
| Hernandez et al. 2019 ([69](#_ENREF_69)) | b ☆ | a ☆ | c | a ☆ | n/a | b | b | d | 3 | low |
| Olaya et al. 2017 ([70](#_ENREF_70)) | b ☆ | a ☆ | b ☆ | a ☆ | n/a | a ☆ | a ☆ | c (loss over 50%) | 6 | low |
| Marengioni et al., 2013 ([71](#_ENREF_71)) | a ☆ | a ☆ | a ☆ | a ☆ | n/a | a ☆ | b | d | 5 | low |
| Craig et al. 2021 ([72](#_ENREF_72)) | b ☆ | a ☆ | c | a ☆ | n/a | b | b | d | 3 | low |
| Gu et al. 2017 ([73](#_ENREF_73)) | b ☆ | a ☆ | b ☆ | a ☆ | n/a | a ☆ | b | d | 5 | low |
| Prazeres et al. 2015 ([74](#_ENREF_74)) | a ☆ | a ☆ | a ☆ | a ☆ | n/a | a ☆ | b | d | 5 | low |
| Chidumwa et al. 2021 ([75](#_ENREF_75)) | a ☆ | a ☆ | a ☆ | a ☆ | n/a | a ☆ | b | d | 5 | low |
| Tan et al. 2015 ([76](#_ENREF_76)) | a ☆ | a ☆ | a ☆ | a ☆ | n/a | a ☆ | b | d | 5 | low |
| Held et al. 2016  ([77](#_ENREF_77)) | b ☆ | a ☆ | c | a ☆ | n/a | b | b | d | 3 | low |
| Costa et al. 2018 ([78](#_ENREF_78)) | b ☆ | a ☆ | c | a ☆ | n/a | b | b | d | 3 | low |
| Marengoni et al. 2009 ([79](#_ENREF_79)) | a ☆ | a ☆ | a ☆ | a ☆ | n/a | a ☆ | a ☆ | c (loss over 40%) | 6 | low |
| Filipcic et al. 2018 ([80](#_ENREF_80)) | b ☆ | a ☆ | c | a ☆ | n/a | b | b | d | 3 | low |
| Bare et al. 2021 ([81](#_ENREF_81)) | c | a ☆ | a ☆ | a ☆ | n/a | a ☆ | b | d | 4 | low |
| Juul-Larsen et al. 2020 ([82](#_ENREF_82)) | c | a ☆ | a ☆ | a ☆ | n/a | a ☆ | a ☆ | a ☆ | 6 | low |
| Han et al. 2022 ([83](#_ENREF_83)) | b ☆ | a ☆ | b ☆ | a ☆ | n/a | a ☆ | b | d | 5 | low |
| Gonsoulin et al. 2017 ([84](#_ENREF_84)) | c | a ☆ | a ☆ | a ☆ | n/a | a ☆ | b | d | 4 | low |
| Roh et al. 2022 ([85](#_ENREF_85)) | b ☆ | a ☆ | c | a ☆ | n/a | b | b | d | 3 | low |
| Zhou et al. 2022 ([86](#_ENREF_86)) | a ☆ | a ☆ | a ☆ | a ☆ | n/a | a ☆ | a ☆ | d | 6 | low |
| Mucherino et al. 2021 ([87](#_ENREF_87)) | a ☆ | a ☆ | a ☆ | a ☆ | a ☆ | a ☆ | b | d | 6 | low |
| Zhu et al. 2019 ([48](#_ENREF_48)) | a ☆ | a ☆ | a ☆ | a ☆ | n/a | a ☆ | b | d | 5 | low |
| Klinedinst et al. 2022 ([88](#_ENREF_88)) | b ☆ | a ☆ | c | a ☆ | n/a | b | a ☆ | d | 4 | low |
| Zheng et al. ([89](#_ENREF_89)) | b ☆ | a ☆ | c | a ☆ | n/a | b | b | d | 3 | low |
| Sibley et al. 2014 ([90](#_ENREF_90)) | b ☆ | a ☆ | c | a ☆ | n/a | b | b | d | 3 | low |
| Hajat et al. 2020 ([91](#_ENREF_91)) | c | a ☆ | a ☆ | a ☆ | n/a | a ☆ | b | d | 4 | low |
| Franti et al. 2022 ([18](#_ENREF_18)) | a ☆ | a ☆ | a ☆ | a ☆ | n/a | a ☆ | b | d | 5 | low |
| Garcia-Olmos et al. 2012 ([92](#_ENREF_92)) | a ☆ | a ☆ | a ☆ | a ☆ | a ☆ | a ☆ | b | d | 6 | low |
| Collerton et al. 2016 ([93](#_ENREF_93)) | a ☆ | a ☆ | a ☆ | a ☆ | n/a | a ☆ | b | d | 5 | low |
| Dorenkamp et al. 2016 ([94](#_ENREF_94)) | b ☆ | a ☆ | c | a ☆ | n/a | b | b | d | 3 | low |
| Aoki et al. 2021 ([95](#_ENREF_95)) | b ☆ | a ☆ | c | a ☆ | n/a | b | b | d | 3 | low |
| Pati et al. 2022 ([96](#_ENREF_96)) | b ☆ | a ☆ | c | a ☆ | n/a | b | b | d | 3 | low |
| Rzewuska et al. 2017 (PNS 2013) ([97](#_ENREF_97)) | b ☆ | a ☆ | c | a ☆ | n/a | b | b | d | 3 | low |
| Carretero-Bravo et al. 2022 ([98](#_ENREF_98)) | b ☆ | a ☆ | c | a ☆ | n/a | b | b | d | 3 | low |
| Batista et al. 2022 ([99](#_ENREF_99)) | b ☆ | a ☆ | c | a ☆ | n/a | b | b | d | 3 | low |
| Puri et al. 2022 ([100](#_ENREF_100)) | b ☆ | a ☆ | c | a ☆ | n/a | b | b | d | 3 | low |
| Whiston et al. 2016 ([101](#_ENREF_101)) | c | a ☆ | c | a ☆ | n/a | b | b | d | 2 | low |
| Cigolle et al. 2012 ([102](#_ENREF_102)) | b ☆ | a ☆ | c | a ☆ | n/a | b | b | d | 3 | low |
| Jackson et al. 2016 ([103](#_ENREF_103)) | c | a ☆ | c | a ☆ | n/a | b | b | d | 2 | low |
| Eyowas et al. 2022 ([104](#_ENREF_104)) | a ☆ | a ☆ | a ☆ | a ☆ | n/a | a ☆ | a ☆ | b ☆ (<20% loss) | 7 | high |
| Lind et al. 2020 ([105](#_ENREF_105)) | a ☆ | a ☆ | a ☆ | a ☆ | n/a | a ☆ | b | d | 5 | low |
| Islam et al. 2014 ([106](#_ENREF_106)) | b ☆ | a ☆ | c | a ☆ | n/a | b | b | d | 3 | low |
| Marventano et al. 2014 ([107](#_ENREF_107)) | b ☆ | a ☆ | b ☆ | a ☆ | n/a | a ☆ | b | d | 5 | low |
| Machon et al. 2020 ([108](#_ENREF_108)) | a ☆ | a ☆ | a ☆ | a ☆ | n/a | a ☆ | a ☆ | d | 6 | low |
| Gu et al. 2018 ([109](#_ENREF_109)) | b ☆ | a ☆ | b ☆ | a ☆ | n/a | a ☆ | a ☆ | a ☆ | 7 | high |
| Deruaz-Luyet et al. 2017  ([110](#_ENREF_110)) | a ☆ | a ☆ | a ☆ | a ☆ | n/a | a ☆ | a ☆ | d | 6 | low |
| Wang et al. 2017 ([111](#_ENREF_111)) | b ☆ | a ☆ | c | a ☆ | n/a | b | b | d | 3 | low |
| Wartelle et al. 2022 ([112](#_ENREF_112)) | b ☆ | a ☆ | a ☆ | a ☆ | n/a | a ☆ | b | d | 5 | low |
| Kuwornu et al. 2014 ([113](#_ENREF_113)) | a ☆ | a ☆ | c | a ☆ | n/a | b | b | d | 3 | low |
| Nunes et al. 2016 ([114](#_ENREF_114)) | b ☆ | a ☆ | b ☆ | a ☆ | n/a | a ☆ | b | d | 5 | low |
| Ruiz et al. 2015 ([115](#_ENREF_115)) | a ☆ | a ☆ | a ☆ | a ☆ | n/a | a ☆ | b | d | 5 | low |
| Guo et al. 2021 ([116](#_ENREF_116)) | b ☆ | a ☆ | c | a ☆ | n/a | b | b | d | 3 | low |
| Yao et al. 2022 ([117](#_ENREF_117)) | b ☆ | a ☆ | c | a ☆ | n/a | b | a ☆ | a ☆ | 5 | low |
| Marengoni et al. 2021 ([118](#_ENREF_118)) | b ☆ | a ☆ | c | a ☆ | n/a | b | a ☆ | b ☆ (<15% loss) | 4 | low |
| Zhang et al. 2022 ([119](#_ENREF_119)) | b ☆ | a ☆ | c | a ☆ | n/a | b | a ☆ | b ☆ (<15% loss) | 4 | low |
| Fan et al. 2022 ([119](#_ENREF_119)) | b ☆ | a ☆ | c | a ☆ | n/a | b | a ☆ | a ☆ | 5 | low |
| Rodreigues et al. 2022 ([120](#_ENREF_120)) | a ☆ | a ☆ | c | a ☆ | n/a | b | b | d | 3 | low |
| Liu et al. 2022 ([121](#_ENREF_121)) | b ☆ | a ☆ | a ☆ | a ☆ | n/a | a ☆ | b | d | 5 | low |
| Zacarias-Pons et al. 2021 ([122](#_ENREF_122)) | b ☆ | a ☆ | c | a ☆ | n/a | b | a ☆ | d | 4 | low |
| Jackson et al. 2015  ([123](#_ENREF_123)) | b ☆ | a ☆ | c | a ☆ | n/a | b | a ☆ | b ☆ (<9% loss) | 4 | low |
| Garin et al. 2014 ([124](#_ENREF_124)) | b ☆ | a ☆ | b ☆ | a ☆ | n/a | a ☆ | b | d | 5 | low |
| Buja et al. 2018 ([125](#_ENREF_125)) | b ☆ | a ☆ | a ☆ | a ☆ | n/a | a ☆ | b | d | 5 | low |
| Clerencia-Sierra et al. 2015  ([126](#_ENREF_126)) | b ☆ | a ☆ | a ☆ | a ☆ | n/a | a ☆ | b | d | 5 | low |
| Aoki et al. 2018 ([127](#_ENREF_127)) | b ☆ | a ☆ | c | a ☆ | n/a | b | b | d | 3 | low |
| Ioakeim-Skoufa et al. 2020 ([128](#_ENREF_128)) | a ☆ | a ☆ | a ☆ | a ☆ | n/a | a ☆ | b | d | 5 | low |
| Dong et al. 2013 ([129](#_ENREF_129)) | a ☆ | a ☆ | a ☆ | a ☆ | n/a | a ☆ | b | d | 5 | low |
| Zador et al. 2019 ([130](#_ENREF_130)) | b ☆ | a ☆ | a ☆ | a ☆ | n/a | a ☆ | b | d | 5 | low |
| Simones et al. 2016  ([131](#_ENREF_131)) | b ☆ | a ☆ | c | a ☆ | n/a | b | b | d | 3 | low |
| John et al. 2003  ([132](#_ENREF_132)) | b ☆ | a ☆ | c | a ☆ | n/a | b | b | d | 3 | low |
| Zheng et al. 2019 ([133](#_ENREF_133)) | b ☆ | a ☆ | b ☆ | a ☆ | n/a | a ☆ | b | d | 5 | low |
| Formiga et al. 2013 ([17](#_ENREF_17)) | b ☆ | a ☆ | b ☆ | a ☆ | n/a | a ☆ | b | d | 5 | low |
| Teh et al. 2018  ([134](#_ENREF_134)) | a ☆ | a ☆ | a ☆ | a ☆ | n/a | a ☆ | a ☆ | d | 6 | low |
| Piotrowicz et al. 2021 ([135](#_ENREF_135)) | b ☆ | a ☆ | c | a ☆ | n/a | b | b | d | 3 | low |
| Juul-Larsen et al. 2018 ([136](#_ENREF_136)) | a ☆ | a ☆ | a ☆ | a ☆ | n/a | a ☆ | b | d | 5 | low |
| Kirchberger et al. 2012 ([137](#_ENREF_137)) | b ☆ | a ☆ | b ☆ | a ☆ | n/a | a ☆ | a ☆ | c (loss 29%) | 6 | low |
| Tan et al. 2020 ([138](#_ENREF_138)) | a ☆ | a ☆ | a ☆ | a ☆ | n/a | a ☆ | b | d | 5 | low |
| Kshatri et al. 2020 ([139](#_ENREF_139)) | b ☆ | a ☆ | a ☆ | a ☆ | n/a | a ☆ | b | d | 5 | low |
| Hunter et al. 2021 ([140](#_ENREF_140)) | a ☆ | a ☆ | c | a ☆ | n/a | b | b | d | 3 | low |
| Lin et al. 2022 ([141](#_ENREF_141)) | a ☆ | a ☆ | a ☆ | a ☆ | n/a | a ☆ | b | d | 5 | low |
| Drajovic et al. 2016 ([142](#_ENREF_142)) | b ☆ | a ☆ | c | a ☆ | a ☆ | b | b | d | 4 | low |
| Craig et al. 2020 ([143](#_ENREF_143)) | b ☆ | a ☆ | c | a ☆ | n/a | b | b | d | 3 | low |
| Poblador-Plou et al. 2014 ([144](#_ENREF_144)) | a ☆ | a ☆ | a ☆ | a ☆ | a ☆ | a ☆ | b | d | 6 | low |
| Ibarra-Castillo et al. 2018 ([145](#_ENREF_145)) | a ☆ | a ☆ | a ☆ | a ☆ | n/a | a ☆ | a ☆ | a ☆ | 7 | low |
| Tang et al. 2020 ([146](#_ENREF_146)) | b ☆ | a ☆ | c | a ☆ | n/a | b | b | d | 3 | low |
| Hsu et al. 2015 ([147](#_ENREF_147)) | b ☆ | a ☆ | b ☆ | a ☆ | n/a | a ☆ | a ☆ | a ☆ | 7 | high |
| Ho et al. 2022 ([148](#_ENREF_148)) | b ☆ | a ☆ | c | a ☆ | n/a | b | a ☆ | b ☆ (<3% loss) | 5 | low |
| Grant et al. 2020 ([149](#_ENREF_149)) | a ☆ | a ☆ | a ☆ | a ☆ | n/a | a ☆ | a ☆ | a ☆ | 7 | high |
| Prenovost et al. 2018 ([150](#_ENREF_150)) | a ☆ | a ☆ | a ☆ | a ☆ | n/a | a ☆ | b | d | 5 | low |
| Zhong et al. 2023 ([151](#_ENREF_151)) | a ☆ | a ☆ | c | a ☆ | n/a | b | b | d | 3 | low |
| Amirzada et al. 2023 ([152](#_ENREF_152)) | a ☆ | a ☆ | b ☆ | a ☆ | n/a | a ☆ | b | d | 5 | low |
| Roomaney et al. 2022 ([153](#_ENREF_153)) | a ☆ | a ☆ | c | a ☆ | n/a | b | b | d | 3 | low |
| Chen et al. 2023 ([154](#_ENREF_154)) | a ☆ | a ☆ | a ☆ | a ☆ | n/a | a ☆ | b | d | 5 | low |
| Ioakeim-Skoufa et al. 2022 ([155](#_ENREF_155)) | a ☆ | a ☆ | a ☆ | a ☆ | a ☆ | a ☆ | b | d | 6 | low |
| Zhong et al. 2022 ([156](#_ENREF_156)) | a ☆ | a ☆ | a ☆ | a ☆ | n/a | a ☆ | b | d | 5 | low |
| Zheng et al. 2020 ([89](#_ENREF_89)) | a ☆ | a ☆ | a ☆ | a ☆ | n/a | a ☆ | b | d | 5 | low |
| Fagbamigbe et al. 2023 ([157](#_ENREF_157)) | a ☆ | a ☆ | a ☆ | a ☆ | a ☆, b ☆ | a ☆ | b | d | 7 | high |
