# Supplementary material for: Identifying clusters of multimorbid disease and differences by age, sex, and socioeconomic status: A systematic review
Source: PLoS One. 2025 Aug 22;20(8):e0329794. doi: 10.1371/journal.pone.0329794 (PMC12373218; doi:10.1371/journal.pone.0329794)
Supplement: S6 Table — (DOCX) [file pone.0329794.s010.docx]

### **Supplementary Table 6. Previous Systematic Reviews.**

| **Title** | **Year Published** | **Databases Used** | **# of Studies Included** | **Population Studied** | **Cluster Technique info** | **Top Clusters Found** | **Study reports on clusters by Age, Sex, Socioeconomic**  **Status** |
| --- | --- | --- | --- | --- | --- | --- | --- |
| **Do replicable profiles of multimorbidity exist? Systematic review and synthesis (**[**29**](#_ENREF_29)**)** | 2019 | 1. Medline  2. EMBASE  3. PsycINFO  4. CINAHL  5. Scopus 6. Web of Science | 51 | General Population - Adults  Majority or studies carried out in Europe or north America | 1. Exploratory factor analysis 2. Cluster analysis of diseases 3. Cluster analysis of people 4. Latent class analysis. | 1. Mental health  2. Cardiometabolic 3. Asthma and COPD  4. Falls, fracture, sensory deficits  5. Parkinson’s disease and cognitive decline | No |
| **Patterns of multimorbid health conditions: a systematic review of analytical methods and comparison analysis (**[**158**](#_ENREF_158)**)** | 2018 | 1. Pubmed 2. Embase | 41 | General Population - Adults | 1. Factor-analysis method (21 studies; 51%) 2. Hierarchical- clustering algorithm (16 studies; 39%) 3. Unified- clustering algorithm 4. Multiple correspondence  5. Network and cluster analyses | i)  Cardiometabolic  diseases ii) Mental health problems iii) Allergic diseases | No |
| **A Systematic Review of the Patterns of Associative Multimorbidity in Asia (**[**159**](#_ENREF_159)**)** | 2021 | 1. MEDLINE 2. EMBASE 3. Cumulative Index to Nursing and Allied Health Literature (CINAHL)  4. Web of Science (Clarivate Analytics) 5. Scopus | 8 | Adults in  Asia | 1. Exploratory factor analysis 2. Observed/expected ratio  3. Logistic regression 4. Hierarchical cluster analysis | i)  Cardiometabolic  ii) Mental health problems iii) Degenerative diseases  iv) Pulmonary diseases v) Cancer diseases | No |
| **Multimorbidity patterns: a systematic review (**[**160**](#_ENREF_160)**)** | 2014 | 1. Medline 2. Embase | 14 | General Population (Adults 15+) | 1. Cluster analysis techniques [21], [24], [25], [26], [29], [30]  2. factor analysis [20], [22], [23], [31], 3. the observed-to- expected ratio [16], [27], [32],  4. multiple correspondence analyses [28] | i) Cardiometabolic diseases, ii) Mental health problems iii) Musculoskeletal disorders. | No |
| **Multimorbidity and quality of life at mid-life: A systematic review of general population studies (**[**33**](#_ENREF_33)**)** | 2018 | 1. PubMed 2. Web of Science 3. Embase 4. APA PsycNET | 8 | Middle aged - adults aged 40–65 years | No | 1. Mental health conditions 2. Cardiovascular disease (CVD) | Reported on Gender if possible |
| **Social determinants of multimorbidity patterns: A systematic review (**[**28**](#_ENREF_28)**)** | 2023 | 1. PubMed,  2. Embase,  3. Scopus,  4. Web of Science,  5. Ovid MEDLINE,  6. CINAHL Complete,  7. PsycINFO 8. Google Scholar | 97 | General population | (1) latent class analysis (42–78);  (2) cluster analysis techniques (79–100);  (3) factor analysis (101–119); ( 4) machine learning methods (120–129);  (5) based on expert knowledge (130–137) | 1. Cardiometabolic,  2. musculoskeletal,  3. mental,  4. respiratory | yes, looks at the social determinents but does not include common denominators: Cardiometabolic multimorbidity profiles were common among men with low socioeconomic status, while musculoskeletal, mental and complex patterns were found to be more prevalent among women. |
